# Late Replication Domains in Polytene and Non-Polytene Cells of *Drosophila melanogaster*

**DOI:** 10.1371/journal.pone.0030035

**Published:** 2012-01-10

**Authors:** Elena S. Belyaeva, Fedor P. Goncharov, Olga V. Demakova, Tatyana D. Kolesnikova, Lidiya V. Boldyreva, Valeriy F. Semeshin, Igor F. Zhimulev

**Affiliations:** Institute of Molecular and Cellular Biology of the Siberian Branch of the Russian Academy of Sciences, Novosibirsk, Russia; Florida State University, United States of America

## Abstract

In *D. melanogaster* polytene chromosomes, intercalary heterochromatin (IH) appears as large dense bands scattered in euchromatin and comprises clusters of repressed genes. IH displays distinctly low gene density, indicative of their particular regulation. Genes embedded in IH replicate late in the S phase and become underreplicated. We asked whether localization and organization of these late-replicating domains is conserved in a distinct cell type. Using published comprehensive genome-wide chromatin annotation datasets (modENCODE and others), we compared IH organization in salivary gland cells and in a Kc cell line. We first established the borders of 60 IH regions on a molecular map, these regions containing underreplicated material and encompassing ∼12% of *Drosophila* genome. We showed that in Kc cells repressed chromatin constituted 97% of the sequences that corresponded to IH bands. This chromatin is depleted for ORC-2 binding and largely replicates late. Differences in replication timing between the cell types analyzed are local and affect only sub-regions but never whole IH bands. As a rule such differentially replicating sub-regions display open chromatin organization, which apparently results from cell-type specific gene expression of underlying genes. We conclude that repressed chromatin organization of IH is generally conserved in polytene and non-polytene cells. Yet, IH domains do not function as transcription- and replication-regulatory units, because differences in transcription and replication between cell types are not domain-wide, rather they are restricted to small “islands” embedded in these domains. IH regions can thus be defined as a special class of domains with low gene density, which have narrow temporal expression patterns, and so displaying relatively conserved organization.

## Introduction

The problem of intercalary heterochromatin (IH) has a history of over 70 years. IH was defined as regions scattered in euchromatic arms of polytene chromosomes and showing a number of features similar to “classic” pericentric heterochromatin (PH) [1]. IH appears as massive dense bands that frequently form ectopic contacts with each other and with PH [2]. In salivary gland polytene chromosomes, IH is transcriptionally inert and completes replication late in the S phase. Eventually IH becomes underreplicated as endocycles that ultimately form polytene chromosomes proceed [3]–[6]. It is underreplication that results in chromosome breaks, originally called “weak spots” by Bridges [7]. Ectopic contacts are likely formed by repair-mediated end-joining of DNA molecules following their underreplication [8], [9].

Underreplication of IH and ectopic pairing are absent from the chromosomes of *SuUR^ES^* (*Suppressor of Underreplication*) mutants. SUUR protein is known to localize to late-replicating regions. Additional doses of *SuUR* gene result in stronger underreplication, higher frequency of chromosome breaks and ectopic pairing [10]–[12].

In polytene tissues, underreplicated regions can be molecularly defined as DNA sequences with decreased copy number [4], [13]. The first experiments using whole-genome transcriptome microarrays allowed identification and molecular mapping of 52 underreplicated regions, thereby providing the first important glimpse into genetic composition of IH. Underreplicated regions were found to be fairly large (100–600 kb) and to contain unique genes (6 to 41) [14]. One of the prominent features of underreplicated regions was that they encompassed small-sized genes with large intergenic regions, i.e. they displayed lower than genome average gene density [15].

IH can be considered as composed of clusters of silent genes that tend to replicate late and so becoming underreplicated. Could such clusters represent basic units of replication regulation? Domain-wide control of replication in eukaryotes is one of the most mysterious and poorly studied phenomena in chromatin biology. Efforts from many groups showed that “units of coordinate replication are stably inherited through multiple cell cycles” ([16] and references therein), yet the mechanisms orchestrating replication timing are still unclear.

Data obtained on mammalian cells suggest that in different cell types replication timing can be quite dynamic, consistent with distinct underlying chromatin states [17]–[20]. It was found that about half of the genome would display altered replication timing at some point in development (reviewed in [21]). Similar comparative analysis in Drosophila, which was performed on cell lines of embryonic (Kc) or neuronal (Cl8) origin also showed significant differences in replication timing, affecting at least 20% of autosomal DNA [22].

It is well-established that replication timing correlates with the state of underlying chromatin. As a rule, late replication is characteristic of repressed chromatin, whereas early replication correlates with open chromatin regions ([22]–[26], [16], [27] for review). Changes in replication status of a large chromosomal domain were speculated to depend on the number of active genes within such domain: integration of the transcriptional activity over large regions appears to mediate early replication timing [27], [28].

In this respect, regions of late replication in Drosophila genome which can be visualized in polytene chromosomes and accurately mapped on a physical map can serve as a convenient model to study the problem of replication regulation at the level of individual domains.

In the present work, we set out to perform detailed analysis of IH domains. To do so, we used the latest genome-wide mapping data available for various protein and chromatin features in Drosophila cell lines [29]–[34]. By integrative analysis of genome-wide binding maps of 53 broadly selected chromatin components in *Drosophila* cells it was shown that the genome can be segmented into five principal chromatin types that are defined by unique combinations of proteins and form specific domains. Each of these chromatin types was conditionally assigned a color: BLUE and BLACK – repressive chromatins, RED and YELLOW – transcriptionally active chromatins, GREEN – heterochromatic domain (see [32] for details and protein compositions of each of the domains). In another work, the analysis of genome-wide chromatin landscape based mainly on 18 histone modifications and several non-histone chromatin proteins, permitted to describe up to 30 combinatorial patterns or states. The simplified model gave 9 states [30].

In this work we aimed to compare the chromatin organization in Kc cell line to that of specific morphological structures found in polytene chromosomes and appearing as IH bands. We wanted to address the following questions: Are there IH-like domains in chromosomes of Kc cells? If so, are they conserved in terms of their transcriptional and replication status? When distinct, are those changes domain-wide or local? We found that in both polytene and Kc embryonic culture cells, IH regions are generally composed of late-replicating chromatin. Differences in transcription and replication patterns are minor and affect only sub-fragments of individual IH bands.

## Results

### Molecular borders of IH bands

IH bands in polytene chromosomes are more than merely underreplicated material. In the absence of underreplication in *SuUR^ES^* mutant, IH bands do become larger [35]. When stronger underreplication is induced with *SuUR^+^* extra-doses, IH bands nevertheless do not disappear and are still quite well-recognizable at the level of cytology. Consistently, for the classic IH region at 75C1-2, both underreplicated and fully replicated zones were experimentally shown to reside within this IH band [36]. Clearly then, even though mapping of IH bands based solely on the positions of underreplication zones is useful in terms that it allows establishing their approximate locations on the physical map [14], accurate mapping of IH band borders requires alternative approaches.

**Figure 1:**
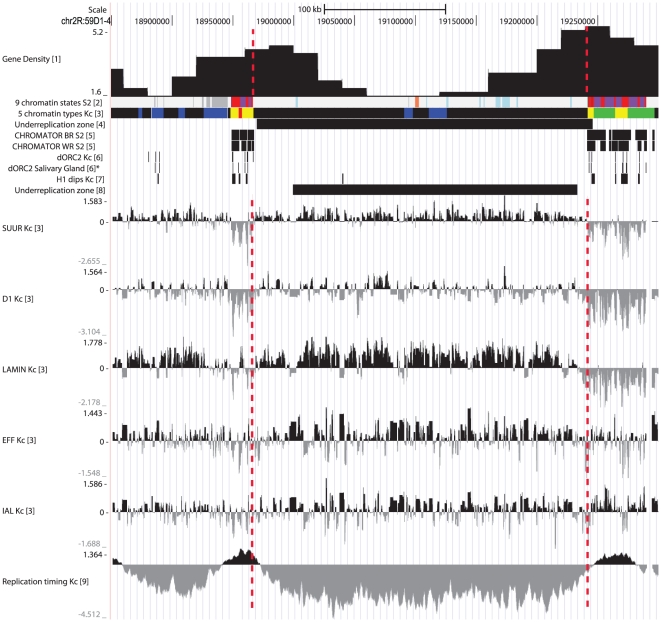
Physical map and molecular features of the band 59D1-2. Vertical lines delimit the borders of this IH band. Data on protein profiling and replication timing are from: (1) – Belyakin et al., 2005 [14]; (2) – Kharchenko et al., 2011 [30]; (3) –Filion et al., 2010 [32]; (4) – Belyakin et al., 2010 [15]; (5) – Kharchenko et al., 2011 [30]; (6) – MacAlpine et al., 2010 [29]; (6)* - Eaton et al., 2011 [37]; (7) – Nordman et al., 2011 [31]; [8] – Schwaiger et al., 2009 [22].

**Figure 2:**
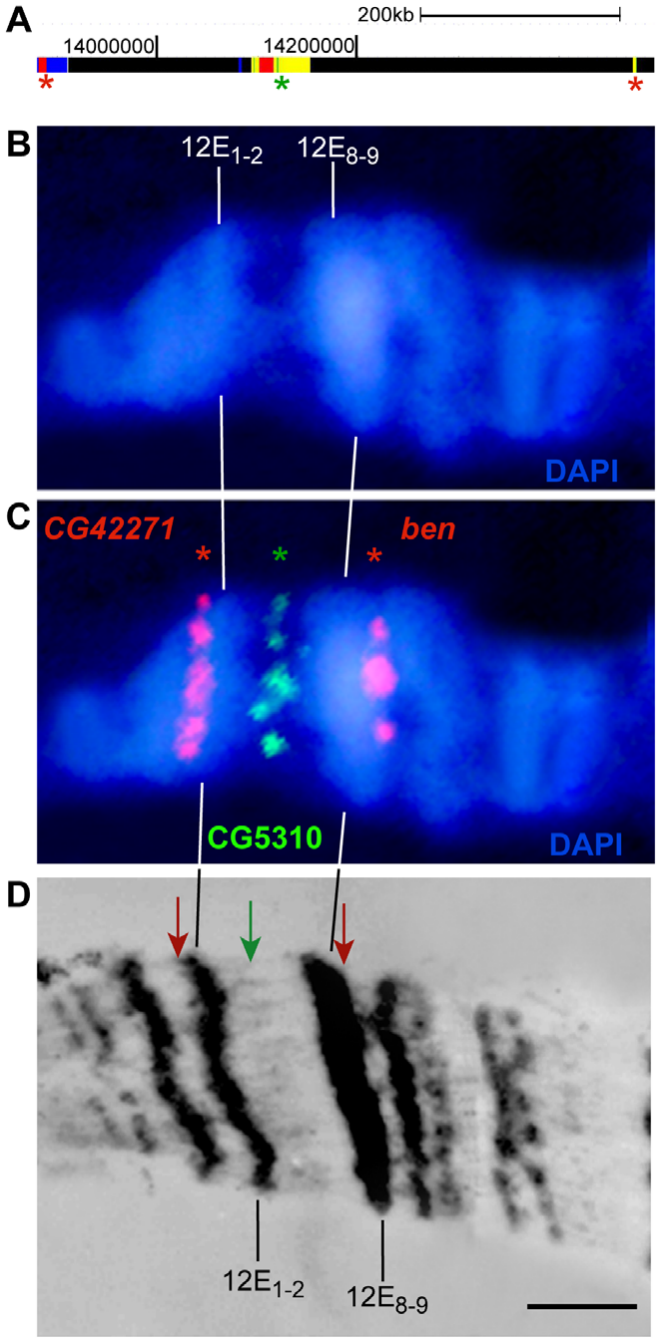
Localization of borders of IH bands with a common underreplication zone at 12E. **A** – molecular map of the region showing colored chromatin as in [32]; **B** – polytene chromosome region, DAPI-stained; **C** – FISH on polytene chromosomes with DNA probes (asterisk on the molecular map) from active “islands” (green) and from the edges (red) of the underreplication zone. Green signal maps to the decondensed regions of polytene chromosome. **D** – EM map of the region.

To achieve this goal, we used published data on chromatin profiling – 5 color types by Filion et al. [32] and 9-state model by Kharchenko et al. [30]. Having compared the color-coded chromatin types with underreplication regions, we observed the latter to mainly correspond to BLACK and BLUE chromatin, two “silent” chromatin types enriched with SUUR, D1 and LAM proteins. In Kc cell line, these chromatin domains are flanked by stretches of YELLOW and RED chromatin, both enriched with active chromatin marks (RNA polymerase II, active histone marks, ORC2) and interband-specific protein CHRIZ/CHRO (hereafter, CHRO) and both depleted for histone H1. Figure 1 illustrates typical chromatin organization around the IH-containing region 59D1-4. Importantly, in a recent study interband regions in polytene chromosomes were shown to display very similar organization in non-polytene chromosomes as well, i.e. interbands display conserved open chromatin organization, they are enriched with ORC-2, depleted for histone H1, typically overlap with YELLOW and RED chromatin regions and are specifically marked with CHRO [38]. Therefore, CHRO localization nearest to the underreplication zone served as the major criterion for interbands that immediately flank IH bands. Additional feature used for delimiting the borders of IH bands was a sharp dip in localization of repressive chromatin proteins SUUR, D1 and LAM (Fig. 1). As is typical of IH, low density of genes is found in these domains. Also, for several regions tested, the DNA probes from CHRO-positive regions adjacent to the repressed domains in Kc cells were shown to hybridize *in situ* to interbands flanking IH bands in salivary glands (example shown on Fig. 2). With this approach in hands, we were able to map the borders of bands corresponding to 50 underreplication zones [14] which have been previously mapped in euchromatin of polytene chromosome arms (underreplication regions at 39DE and 40AE were omitted from this analysis due to their repeated nature (histone gene cluster at 39DE) or proximity to PH and poor cytology of the region which hindered cytological mapping of the region 40AE).

**Figure 3:**
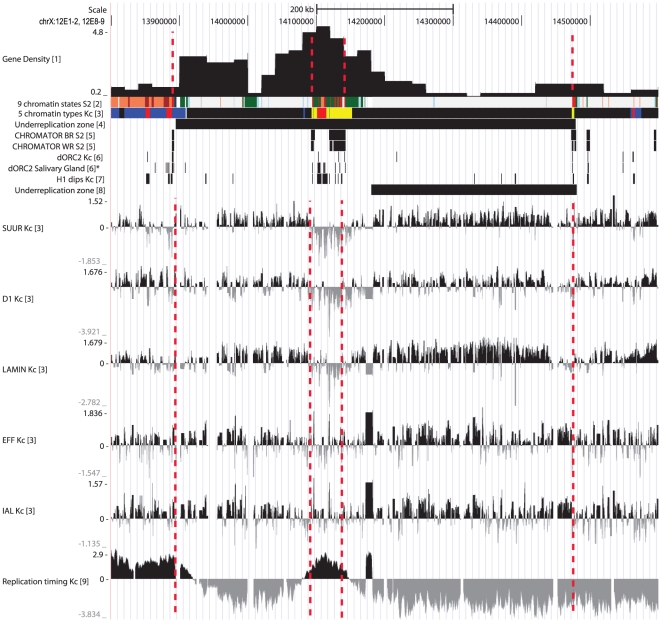
Physical map and molecular features of the region 12E. Legends are the same as on Figure 1. The region consists of two bands, 12E1-2 (left) and 12E8-9 (right).

We estimated that out of 50 underreplication regions, 40 were represented as single bands on the Bridges map [7]. For the remaining 10, we observed the underreplicated regions to have islands of “interband” material marked with CHRO, suggesting that such regions are composed of two separate bands in polytene chromosomes. To test this suggestion, we performed fluorescence *in situ* hybridization (FISH) on polytene chromosomes with DNA probes from such “interband”-like regions. Figures 2 and 3 show the example of such analysis for the region 12E, where hybridization signal clearly maps to the decondensed regions between the bands 12E1-2 and 12E8-9 in polytene chromosome. Thus, the island of open chromatin is present in both Kc cell line and in polytene chromosomes, indicative of the existence of two separate IH bands (12E1-2 and 12E8-9) both of which fall into the underreplication region mapped in [14]. Besides 12E, the list of regions with similar organization includes 19E, 35D, 56AB, 58A, 70A, 84D, 87D, 89A and 92DE (verified using FISH), thereby each of these regions consists of two adjacent bands (Fig. S1). Thus, our list of IH regions comprises 40 single bands and 10 regions with two bands, i.e. 60 bands in total. Table 1 shows their accurate nomenclature and span as established via analysis of colored chromatin maps, binding profiles for the marker proteins, and our FISH data. The total length of IH bands analyzed in the present work is 14772 kb, i.e. 12.4% of euchromatic portion of the genome. IH bands range from 68 to 640 kb, being ∼250 kb on average, and comprise about 7% of *Drosophila* genes. Passports for all 60 IH bands are given in Figure S1.

**Table 1:** Nomenclature, sizes of IH regions and overlapping with LADs.

Cytological position	Chrom. arm	Start	End	Size (bp)	Overlap with LADs (%)*	Number of LADs
4D1-2	X	4 602 210	4 798 095	195 885	90,04%	1
7B1-2	X	7 219 818	7 587 824	368 006	79,65%	1
9A3	X	9 764 004	9 902 414	138 410	100,00%	1
11A6-9	X	11 924 233	12 355 859	431 626	96,68%	1
11D1-2	X	12 805 796	12 980 471	174 675	84,89%	1
12E1-2	X	13 891 495	14 092 986	201 491	53,47%	1
12E8-9	X	14 142 573	14 473 303	330 730	88,26%	1
13B3-4	X	15 033 921	15 186 587	152 666	94,13%	1
19A1-4	X	19 760 353	20 002 484	242 131	83,63%	1
19E1-2	X	20 396 867	20 525 924	129 057	92,71%	1
19E3-4	X	20 530 295	20 898 146	367 851	96,69%	1
23A1-2	2L	2 586 752	2 729 811	143 059	60,82%	1
25A1-4	2L	4 465 899	4 794 750	328 851	93,70%	1
26C1-2	2L	6 129 581	6 323 263	193 682	0,00%	0
32A1-2	2L	10 529 678	10 727 544	197 866	89,59%	1
33A1-2	2L	11 518 408	11 788 020	269 612	96,95%	1
34A1-2	2L	12 723 201	12 973 660	250 459	95,95%	1
35B1-2	2L	14 363 195	15 003 691	640 496	86,70%	5
35D1-2	2L	15 276 150	15 497 849	221 699	91,32%	1
35D3-4	2L	15 500 517	15 745 052	244 535	80,36%	1
35E1-2	2L	15 913 979	16 250 562	336 583	96,26%	1
36C1-2	2L	16 911 777	17 367 919	456 142	51,96%	1
36D1-4	2L	17 503 330	18 137 736	634 406	95,76%	1
47A1-2	2R	6 198 857	6 304 091	105 234	97,07%	1
50C1-4	2R	9 482 096	9 692 212	210 116	73,53%	1
53C1-2	2R	12 236 248	12 458 042	221 794	63,05%	1
56A1-2	2R	14 741 665	14 857 648	115 983	88,95%	1
56B1-2	2R	14 865 557	15 008 584	143 027	82,01%	1
57A1-4	2R	16 216 605	16 438 659	222 054	93,49%	1
58A3-4	2R	17 608 824	17 857 069	248 245	94,02%	1
58B1-2	2R	17 862 128	17 947 421	85 293	89,37%	1
59D1-4	2R	18 967 254	19 241 608	274 354	96,66%	1
64C1-2	3L	4 627 831	4 823 782	195 951	68,72%	1
64C3-4	3L	4 827 301	5 125 950	298 649	76,35%	1
64D1-2	3L	5 362 810	5 552 370	189 560	0,00%	0
67D9-12	3L	9 967 967	10 217 044	249 077	95,58%	1
70A1-2	3L	13 039 472	13 221 452	181 980	97,62%	1
70A4-5	3L	13 227 736	13 379 716	151 980	100,00%	1
70C1-2	3L	13 507 892	13 852 887	344 995	94,09%	1
71C1-2	3L	15 227 917	15 490 824	262 907	95,92%	1
75C1-2	3L	18 108 214	18 610 726	502 512	83,16%	1
77E1-4	3L	20 535 141	20 761 092	225 951	92,56%	1
79E1-4	3L	22 282 975	22 708 149	425 174	81,28%	1
83E1-2	3R	1 836 823	2 169 729	332 906	88,13%	1
84A1-2	3R	2 284 961	2 470 765	185 804	84,91%	1
84D3-4	3R	3 076 774	3 297 717	220 943	90,16%	1
84D9-10	3R	3 367 789	3 634 554	266 765	0,00%	0
86D1-2	3R	6 720 694	6 953 927	233 233	0,00%	0
87B1-2	3R	7 844 557	7 912 503	67 946	0,00%	0
87B4-5	3R	7 916 875	8 043 542	126 667	58,95%	1
87D1-2	3R	8 544 139	8 786 732	242 593	88,42%	1
89A1-2	3R	11 374 360	11 475 824	101 464	100,00%	1
89A8-9	3R	11 504 224	11 611 210	106 986	50,76%	1
89E1-4	3R	12 482 908	12 811 745	328 837	0,00%	0
92D1-4	3R	15 885 860	16 078 103	192 243	82,71%	1
92E1-2	3R	16 156 729	16 374 812	218 083	92,71%	1
94A1-4	3R	17 868 784	18 181 159	312 375	43,50%	1
98C1-2	3R	23 533 481	23 740 483	207 002	85,06%	1
100A1-2	3R	26 428 777	26 590 043	161 266	95,07%	1
100B1-2	3R	26 715 776	26 877 988	162 212	100,00%	1

*% overlap was calculated as a ratio between the length of overlapping region and the length of IH band.

### Molecular characteristics of IH bands

Having established the molecular coordinates of IH bands' borders, we proceeded to describe the properties of IH chromatin and to compare the chromatin states in polytene cells and Kc cell line. In polytene chromosomes, IH is tightly packed and genetically silent [6]. Transcriptional silencing of genes in underreplicated regions of salivary glands has recently been directly demonstrated using RNA-seq analysis of RNA from larval salivary glands [39]. To analyze chromatin characteristics of IH regions in Kc cell line, we used modENCODE project [29]–[31] and Filion et al. [32] datasets. Each of the 60 IH bands analyzed was given a “passport” showing its most prominent features (Fig. 1, 3, S1).

**Figure 4:**
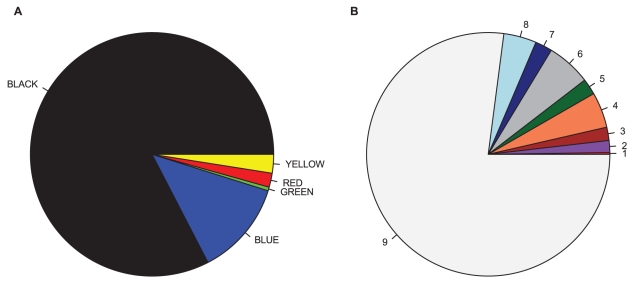
Proportion of various chromatin types in IH regions. **A** – 5 color chromatin types by [32]. **B** – 9 chromatin states as in [30] (states 6–9 correspond to repressed chromatin).

In Kc cell line, the common theme for most of the regions corresponding to IH of polytene chromosomes was their repressed state: 97% of the total length of 60 IH regions was composed of silent chromatin types: BLACK and BLUE (84 and 13%, respectively) (Fig. 4**A**). Whereas the mechanism underlying silenced state of BLUE chromatin is quite well explored and involves the action of PC-G proteins, little is known about how BLACK chromatin is repressed [32]. IH domains are heterogeneous in their chromatin types: 12 are fully BLACK (with 4 regions encompassing small islands of HP1-dependent GREEN chromatin), 1 region, 89E1-4 (BX-C), is entirely BLUE; 28 regions show alternating stretches of BLACK and BLUE chromatins; 19 regions have fragments of YELLOW and RED (“active”, yet, CHRO-negative) chromatin in largely BLACK and BLUE environment. The total length of such open chromatin fragments constitutes about 2% of the total length of IH bands, ranging from 1 to 15 kb. Positions of RED and YELLOW chromatin fragments as a rule coincide with localization of active regions in S2 cells (states 1–3) of a 9-state model from modENCODE (Fig. 4**B**).

It is interesting to note that as a rule, RED fragments in IH are flanked with BLUE chromatin (87%), with the remaining 13% being bordered by BLACK from one side. YELLOW chromatin fragments (15 in total) are always embedded in BLACK.

Figure 4 demonstrates general correspondence between chromatin states of IH domains in cell lines. Overall, ratios of active and inactive chromatins are similar between the two approaches [30], [32]: in both Kc and S2 cells IH is mostly represented by repressed chromatin totaling 97% and 89%, respectively.

As it could be expected from the principles of assigning the chromatin types their colors [32], IH bands are enriched with SUUR, D1 and LAM. Their distribution profiles have sharp borders, and typically these proteins are absent from the RED and YELLOW chromatin regions embedded within IH bands (Fig. 1, 3, S1). Two other “silent” chromatin proteins, - IAL and EFF, are weaker markers of IH. In contrast to SUUR, D1 and LAM which show very similar enrichment profiles and tend to co-localize, IAL and EFF display weaker correlation and are frequently found in RED and YELLOW chromatin (Table 2).

**Table 2:** Proportion of the DNA sequences covered by the corresponding proteins (%).

Type of sequences	SUUR	D1	LAMIN	EFF	IAL	ORC2
Total genome	69,47	51,93	44,83	13,26	17,46	1,09
IH bands	91,00	74,28	74,63	21,47	24,41	0,13
Interbands	32,77	17,57	11,62	4,36	6,08	6,14

Strong enrichment of LAM in IH is consistent with the localization of silent chromatin on the periphery of cell nucleus, and in particular with the observations that IH bands are frequently found associated with the nuclear lamina [40]. With the exception of one region (89E1-4), all IH regions that we analyzed using datasets from [32] display prominent LAM binding, which typically plummets at the IH domain borders and correlates well with the distribution of SUUR and D1 (Fig. S1). One could thus extrapolate that IH bands should by default correspond to Lamin-associated domains (LADs). Much like IH bands, LADs which were recently described in mammalian and fruitfly genomes, are composed of repressed chromatin [40], [41]. We compared published localization of LADs [41] and IH regions. Surprisingly, the overlap was far from complete (% overlap is indicated in Table 1, last column). As it turned out, 6 IH bands showed no overlap with any of the LADs (26C1-2, 64D1-2, 84D9-10, 86D1-2, 87B1-2, 89E1-4), and one IH band (35B1-2) encompassed five separate LADs. Complete overlap (100%) was only observed for 4 IH regions (9A3, 70A4-5, 89A1-2, 100B1-2). In the rest of the cases, IH bands and LADs displayed partial overlap ranging from 97 to 43%, with their borders frequently shifted away (up to 300 kb) from each other. Thus, the question of whether these two domain types are truly related needs further clarification.

### Replication timing in IH bands

All IH regions in salivary gland polytene chromosomes replicate late in the S-phase. Late replication and its extreme form, underreplication, are the major markers of IH. We analyzed the replication status of these regions in Kc cells using the data from [22]. As much as 80% (48 out of 60) of IH regions turned out to be entirely late-replicating in Kc cell line; the remaining 20% displayed local changes from late to early replication.

Thus, IH domains in salivary gland cells and Kc cell line display highly conserved replication timing, consistent with their highly similar, repressed chromatin state. The magnitude of changes in replication timing between the cell types is of the same order as between different cell lines (20–25%), according to [22].

IH bands are depleted for ORC-2, which can be considered as a marker of potential origins of replication. Using ORC-2 binding data obtained for salivary gland polytene chromosomes [37], we confirmed 34 IH bands as completely lacking ORC-2 binding, 19 bands showing 1–2 enrichment peaks, and only 7 bands displaying more than 2 binding regions.

In contrast, interband regions are enriched in ORC-2. We compared ORC-2 binding site density in IH bands and adjacent interbands and we estimated 1 Mb of interband DNA to comprise 220 ORC-2 binding sites, whereas IH bands displayed 7 sites per 1 Mb. Differences of the same order of magnitude are observed for the normalized length of ORC2-bound DNA in IH bands and in interbands (Table 2). Thus, repressed state and late replication in IH bands correlate with dramatic depletion for replication origins.

Hence, interband material replicates early: of 110 interbands flanking the IH bands analyzed, 99 can be classified as early-replicating, and only 11 regions predicted as interbands lack any markers of early replication (Fig. 1, 3, S1).

Overall, sequence of replication phases in *D. melanogaster* chromosomes is well-known. In early S phase, numerous active regions replicate (“continuous labeling” phase). At the subsequent phases of “discontinuous labeling”, silent regions of the genome including IH are replicated. Finally, in late S phase, replication is only observed in the pericentric heterochromatin [42]–[44]. When analyzing replication dynamics, we used these criteria originally established via ^3^H-thymidine incorporation.

**Figure 5:**
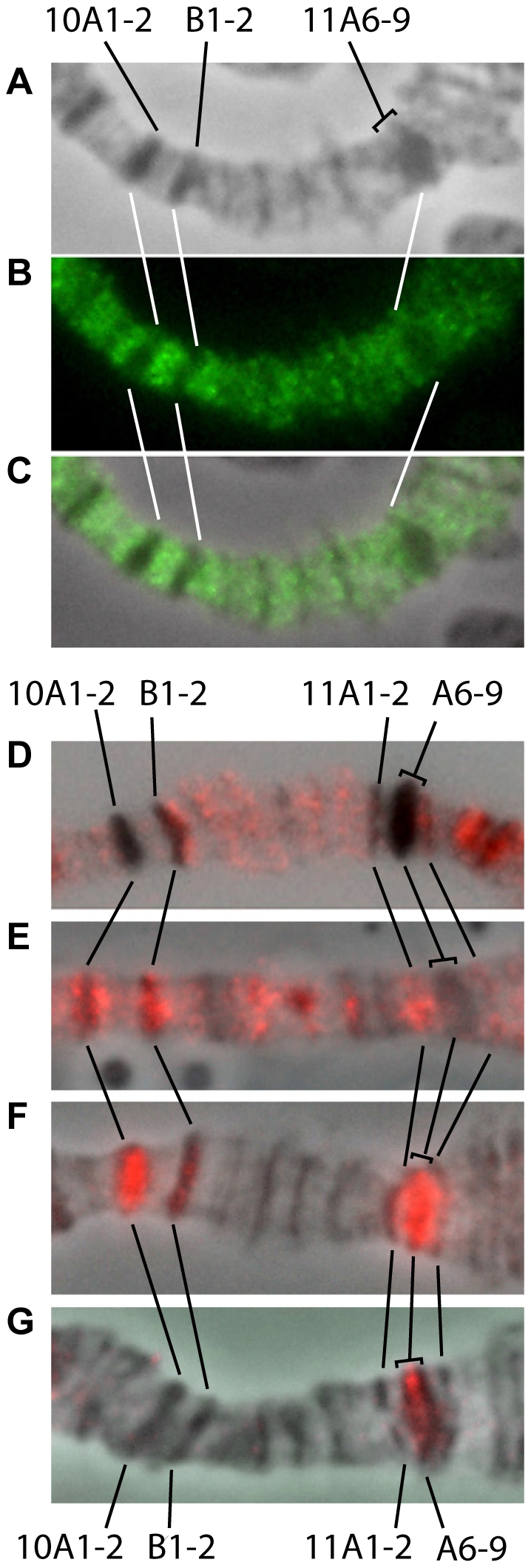
DNA replication in 10A-11A region of the polytene chromosome. **A–C** – Immunostaining for pre-replication complex component DUP/CDF1. Pre-replication complex is not detected in IH bands 10A1-2, 10B1-2, 11A6-9. **A** – phase contrast; **B** – immunolocalization; **C**- merge; **D–G** – immunostaining for PCNA at consecutive replication steps (further description in text).

Immunostaining allows for greater resolution of replication dynamics in different cytological structures. We used PCNA-specific antibodies (marker of replication) and DUP/CDT1 (hereafter, DUP, marker of pre-replication complexes) to conclude that IH bands not only complete replication later (which has been shown previously), but also start replication with a delay. This is illustrated by the X-chromosome region 10A-11A (Fig. 5). Pre-replication complexes are known to assemble in G1. Upon entering S-phase, sequential origin activation occurs, however no new pre-replication complexes are formed. Such origin licensing assures that all genomic sequences are replicated only once per cycle [45], [46]. Figure 5**A, B, C** shows that distribution of pre-replication complexes along the chromosome region has clear gaps that correspond to large bands 10A1-2, 10B1-2, 11A6-9. This reinforces the observation that there are very few if any origins of replication in IH regions [39]. Figure 5**D** shows that at an early replication step, PCNA is found in interbands and in faint partially decondensed bands; dense bands 10A1-2, 10B1-2 and 11A6-9 are PCNA-negative. At the next step, 10B1-2 enters replication, 10A1-2 shows labeling on the flanks, and 11A6-9 remains negative (Fig. 5**E**). Then, 11A6-9, 10A1-2 and 10B1-2 replicate whereas the rest of the structures in the region have already completed replication (Fig. 5**F**). Finally, PCNA signal is detected only in the center of 11A6-9, a typical underreplicated region (Fig. 5**G**). Thus, IH bands start replication with a delay, and replicate from the borders inwards, showing no “internal” origins of replication. These data were generated in *SuUR^ES^* mutant background, where underreplication is suppressed and so a finer analysis of S phase progression is possible. Despite the lack of underreplication in *SuUR^ES^* mutants, the sequence of replication completion remains the same as in the wild-type, i.e. IH bands remain late-replicating in *SuUR^ES^* mutants [12]. Also, *SuUR^ES^* mutation has no effect on the number of ORC-binding sites in underreplicated regions [39]. So, we believe that replication pattern described above corresponds to the wild-type situation (further details on replication dynamics in wild-type and *SuUR^ES^* mutants will be given elsewhere). So, *SuUR^ES^* background is very convenient in that DNA in IH bands is fully replicated. This makes possible reliable detection of a feature of interest (for instance, PCNA) in the center of the IH band, i.e. in a region that is strongly underreplicated in wild-type chromosomes.

We observe that the length of DNA in IH bands correlates with later completion of replication. Vast majority of bands that replicate the latest in the genome [12] are also the largest, spanning over 300 kb. The most prominent IH bands in this class are 35B1-2 and 36E1-4, both well-known “champions” of late replication, spanning over 600 kb each.

**Figure 6:**
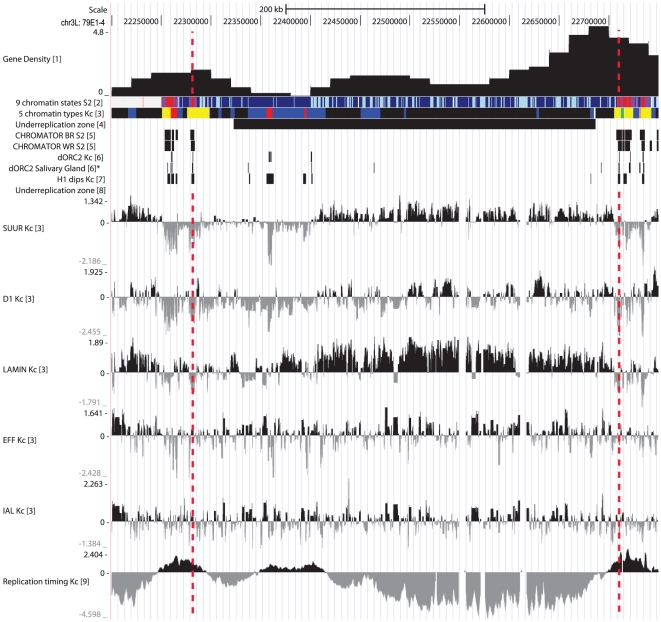
Physical map and molecular features of the region 79E1-4. Legends are the same as on Fig. 1. IH band has an early-replicating region, which corresponds to two active (RED) fragments.

As a rule, differences in replication timing between IH bands in salivary glands and in the respective regions of chromosomes in cell culture correlate with the presence of YELLOW and RED chromatin in these regions, i.e. with transcriptional activity of local sub-regions of these bands. Such differences (late replication in polytene cells, and early replication in diploid cell lines) were observed for 12 IH bands, with restricted, local effects. In 9 such bands, changes in replication timing were clearly linked to the presence of open chromatin types, as shown in Figure 6. In 3 IH bands (32A1-2, 58A3-4 and 100B1-2) the emerging early replication peaks are independent of chromatin changes and are found in the BLUE or BLACK chromatin context. Interestingly, replication timing as a rule switches from late to early in IH regions, where open chromatin is at least 5 kb long (Table 3). In contrast, in IH bands where open islands span less than 5 kb, replication timing remains late with one notable exception at IH band 36D1-4. Table 3 summarizes the data on how sizes of open chromatin fragments relate to the lengths of early replication areas within IH bands. It must be noted, that we only considered the regions where open chromatin fragments localized in the center of the bands. This allows to clearly differentiate two zones of early replication, in interbands and in inner parts of bands. Apparently there must exist a certain length threshold that defines early replication of “active” island, although there is a formal possibility that smaller regions that replicate early in otherwise late-replicating context are less likely to be reproducibly mapped on replication profiles.

**Table 3:** Correlation of sizes of open chromatin (RED and YELLOW) fragments with the length of early replication areas.

IH band	Total length of “open” chromatin fragments (kb)	Size of the zone with early replication (kb)
75C1-2	1.0	0
53C1-2	2.1	0
56A1-2	2.4	0
77E1-4	3.3	0
94A1-4	3.3	0
71C1-2	5.1	13.7
84D9-10	5.6	8.3
36D1-4	8.8	0
79E1-4	8.1	67.3
64C1-2	9.1	73.5
70A4-5	12.1	113.9
35B1-2	14.9	136.3
19A1-2	21.9	118.1
34A1-2	26.4	60.1
7B1-2	34.7	∼200

Thus, in most cases where in contrast to salivary gland polytene chromosomes, bands in Kc cell line show “active” chromatin embedded in silenced domains, this is accompanied with changes in replication timing.

Consistent with the late-to-early changes in replication timing, we observed concomitant loss of SUUR, D1 and LAM. Most clearly this was seen for the SUUR protein, whose enrichment profiles had particularly sharp borders. It must be noted that regions affected by the shift from late to early replication as a rule are several-fold larger than the total length of respective open chromatin fragments within the IH domain (Table 3).

## Discussion

The major focus of the present work was to compare organization of IH regions in polytene chromosomes and in the Kc cell line (of embryonic origin). In contrast to the general genome-wide replication studies, we chose to specifically analyze changes in replication timing in individual domains. These domains are of similar molecular and cytological make-up: in polytene chromosomes they comprise coordinately late-replicating clusters of silent genes. Overall they encompass ∼14 Mb. Thus, the 60 IH regions studied represent a significant fraction of repressed chromatin in Drosophila genome, and were previously mapped and characterized based on their underreplication in the S phase [14].

Underreplication is not restricted to polytene chromosomes from salivary glands. For instance, it is also found in polytene chromosomes from fat body endocycling cells [4], [47]. Existence of *SuUR*-dependent underreplication was also demonstrated for the polytene chromosomes from pseudonurse cells in *otu* mutants [48], [49]. Recently, tissue-specificity of underreplication was demonstrated via genome-wide profiling of three cell types, namely salivary gland, fat body and midgut cells [31]. The authors identified 24 underreplication zones, of which 20 were located in a euchromatic portion of the genome. Localization of underreplicated regions in salivary gland and midgut cells was quite similar, whereas fat body cells were distinct in that they had fewer underreplicated regions which were often found in alternative genomic locations.

Differences in numbers of underreplicated regions mapped in salivary glands by Belyakin et al. [14] and Nordman et al. [31] are first and foremost due to the fact that the former group used a stock with two extra-doses of *SuUR* gene, thereby displaying increased underreplication as compared to the wild-type background. This might explain why the number of underreplicated regions identified by Belyakin et al. [14] is much greater (52) than that found by Nordman et al. [31] in the wild type strain (15). *SuUR^+^* expression is known to result in stronger underreplication, even though it does not significantly change the borders of underreplicated regions [13]. Overall, both analyses reported very similar positions of underreplication zones in salivary glands (Fig. S1).

In general, tissue specificity of underreplication is consistent with the data about plasticity of replication domains. Over 20% of DNA sequences in the genome were found to show distinct replication timing in different cell types [18], [22].

In the present work, we used localization of underreplication zones to map IH regions to the genome, and to molecularly map the borders of individual IH bands. As a result, we analyzed 60 late-replicating IH bands, which showed highly similar organization in polytene and diploid cells.

Genes residing in IH tend to function in a narrow temporal patterns. Notably, many of such genes are male-specific, active in the male germline and are organized in clusters [50] that are found in 80% of IH bands [14], [15]. Consistently, analysis of gene expression in BLACK chromatin suggests that it is also enriched in genes with narrow developmental expression patterns [32], which could possibly be attributed to long intergenic regions in IH [15] and increased frequency of highly conserved non-coding elements [32].

The fact that only a fraction of genes in IH of Kc cells displays distinct expression patterns clearly argues that expression of such genes is independent of the rest of the genes within these domains. Also, many IH domains are composed of different types of repressed chromatin, i.e. besides PC-G-dependent silencing (BLUE chromatin), IH domains encompass many genes repressed by other, yet to be determined factors (BLACK chromatin). Taken together, these observations suggest that IH does not function to organize domain-wide expression. Furthermore, developmental changes in replication timing within an individual IH band only affect its sub-regions, and so it is unlikely that IH regions in Kc cells correspond to units of coordinated replication control.

Replication timing in IH regions of salivary glands is not only characterized by its late onset, it also continues longer, until the very end of S phase. What are the mechanisms underlying late completion of replication? One of such mechanisms involves inhibition of replication fork progression by SUUR protein [39]. Recently it has become clear that the prominent factor that actually defines replication status of the region is the density of replication origins. ORC-2 binding serves to mark origins of replication, and its binding is very low in silent and SUUR-enriched bands composed of BLACK and BLUE chromatin. Most of ORC-2 binding is concentrated in open chromatin, according to [29], [39]. We estimate that there is about 50-fold difference between IH bands and interbands in terms of ORC-2 density (Table 2), and many IH bands are completely devoid of ORC-2. This effect has been generally described as a correlation of inter-origin lengths with their later replication timing [29]. If IH band lacks internal origins of replication, it can be considered as a single DNA fragment between the origins located on the flanks. Clearly then, the larger the IH band is, the later its replication will end, and so the greater is the chance it eventually becomes underreplicated. This is supported by the analysis of replication dynamics in polytene chromosomes. According to our observations, IH bands start replication with a delay: replication begins in interbands, proceeds to the edges of condensed bands and ends in their centers. If replication fails to complete on time, underreplication zone is formed in the center of the band. Consistently, the largest bands are the last to complete replication. This conclusion is further supported by the comparison of IH band lengths (Table 1) with the timing of their replication completion [12]. Apparently, IH domains devoid of internal origins of replication correspond to those described in [22], as beginning to replicate in early- and mid- S phase and continuing until the late S phase.

Studies in mammalian cells have resulted in a concept that replication timing changes are regulated at the level of large domains, and that changes in replication timing could rapidly propagate a change in chromatin structure across hundreds of kilobases (reviewed in [21]) Irrespective of the species used, replication domains varied widely in size, whereas those domains that changed replication depending on the cell type, were typically 400–800 kb. Therefore, this number could serve as a size estimate for the minimal basic unit of replication-timing control [17]–[20].

Drosophila studies also demonstrated that replication timing changes can involve large chromatin domains, yet the figures for the minimal domain size have not been reported, since regional differences below 20 kb were excluded from the analysis [22]. Whatever were the case, such domains in drosophila, averaging 180 kb, are much smaller than megabase-sized replication domains in mammals [17], [51], [52].

According to our analysis, the differentially replicating sub-regions in IH domains can be rather short. Their size is dependent on the number and span of the active DNA fragments (Table 3), but is always smaller than the size of the IH domain. It is interesting to note that early replication in such cases is generally observed if active “islands” are greater than 8–10 kb, particularly if these “islands” are clustered together. If smaller “islands” of RED or YELLOW chromatin are found in the IH bands, as a rule this material is late-replicating. Possibly, for the timing of replication to be switched, the length of an open chromatin region should reach a certain threshold, as it was previously proposed [27], [28]. Thus, in Kc cell line, large domains of late replication sometimes break into smaller early- and late-replicating sub-domains, and so they can not be considered as permanent units of replication control. Despite this conclusion, we consider IH regions as a special class of genomic domains. These domains are distinguished by lower average gene density. This feature combined with large sizes of IH domains lies in the core of their conservative organization as a cluster of independently regulated genes with narrow temporal patterns of expression.

## Materials and Methods

### Use of modENCODE protein localization data

The data from Fly modENCODE (http://www.modencode.org) project were used. The data were accessed either on the corresponding pages in GEO (http://www.ncbi.nlm.nih.gov/geo/), or from the supplementary materials to the original papers. We used two types of data from modENCODE: smoothed M-value enrichment profiles and regions of significant enrichment. Protocols for data processing are described in the corresponding section of modMine (http://intermine.modencode.org).

For visualization of data we used UCSC Genome browser (http://genome.ucsc.edu). Custom scripts were used to convert the data to the UCSC format.

Positions of LADs [41] and IH bands are given in coordinates of *Drosophila melanogaster* genome sequence release 5.

### Immunofluorescence microscopy

Flies were raised on standard cornmeal-yeast-agar molasses medium at 22°. Stocks with *SuUR^ES^* [10] background, where underreplication is suppressed, were used.

Fluorescence *in situ* hybridization was performed as described in [53]. To obtain probes from the 12E region, genomic DNA was PCR-amplified using the following primers: CG42271 (5′- acgggcacggacaactcctc -3′ and 5′- cgacaaggagggcctgctca -3′, 716 bp), CG5310 (5′- gtgcctgggcacatccttaaatcc -3′ and 5′- tccatctacggcagggtgttgt -3′, 738 bp), ben (5′- cacccaaccctgcacacacg -3′ and 5′- atggcctccgcctcgttgac -3′, 783 bp). DNA probes were labeled with biotin-16-dUTP or digoxigenin-11-dUTP (Roche) in random-primed polymerase reaction using Klenow fragment.

Immunostaining was performed as described in [54]. Primary antibody dilutions used were as follows: mouse monoclonal anti-PCNA (PC10, Abcam, ab29) - 1∶500; guinea pig anti-DUP (kindly provided by Dr. Terry Orr-Weaver [55]) 1∶500. The slides were incubated with secondary Texas Red-labeled goat anti-mouse IgG specific conjugates (ab-6787, Abcam) - 1∶500 and Alexa Fluor 488 goat anti-guinea pig antibodies - 1∶500.

Chromosomes were examined using epifluorescence optics (Olympus BX50 microscope) and photographed with CCD Olympus DP50.

## Supporting Information

Figure S1 **Passports of the IH bands.** Vertical lines delimit the borders of this IH band. Data on protein profiling and replication timing are from: (1) – Belyakin et al., 2005 [14]; (2) – Kharchenko et al., 2011 [30]; (3) –Filion et al., 2010 [32]; (4) – Belyakin et al., 2010 [15]; (5) – Kharchenko et al., 2011 [30]; (6) – MacAlpine et al., 2010 [29]; (6)* - Eaton et al., 2011 [37]; (7) – Nordman et al., 2011 [31]; [8] – Schwaiger et al., 2009 [22].
(PDF)
