# Supplementary material for: Late Replication Domains in Polytene and Non-Polytene Cells of Drosophila melanogaster
Source: PLoS One. 2012 Jan 10;7(1):e30035. doi: 10.1371/journal.pone.0030035 (PMC3254639; doi:10.1371/journal.pone.0030035)
Supplement: Figure S1 — Passports of the IH bands. Vertical lines delimit the borders of this IH band. Data on protein profiling and replication timing are from: (1) – Belyakin et al., 2005 [14]; (2) – Kharchenko et al., 2011 [30]; (3) –Filion et al., 2010 [32]; (4) – Belyakin et al., 2010 [15]; (5) – Kharchenko et al., 2011 [30]; (6) – MacAlpine et al., 2010 [29]; (6)* - Eaton et al., 2011 [37]; (7) – Nordman et al., 2011 [31]; [8] – Schwaiger et al., 2009 [22]. (PDF) [file pone.0030035.s001.pdf]

4D1-2

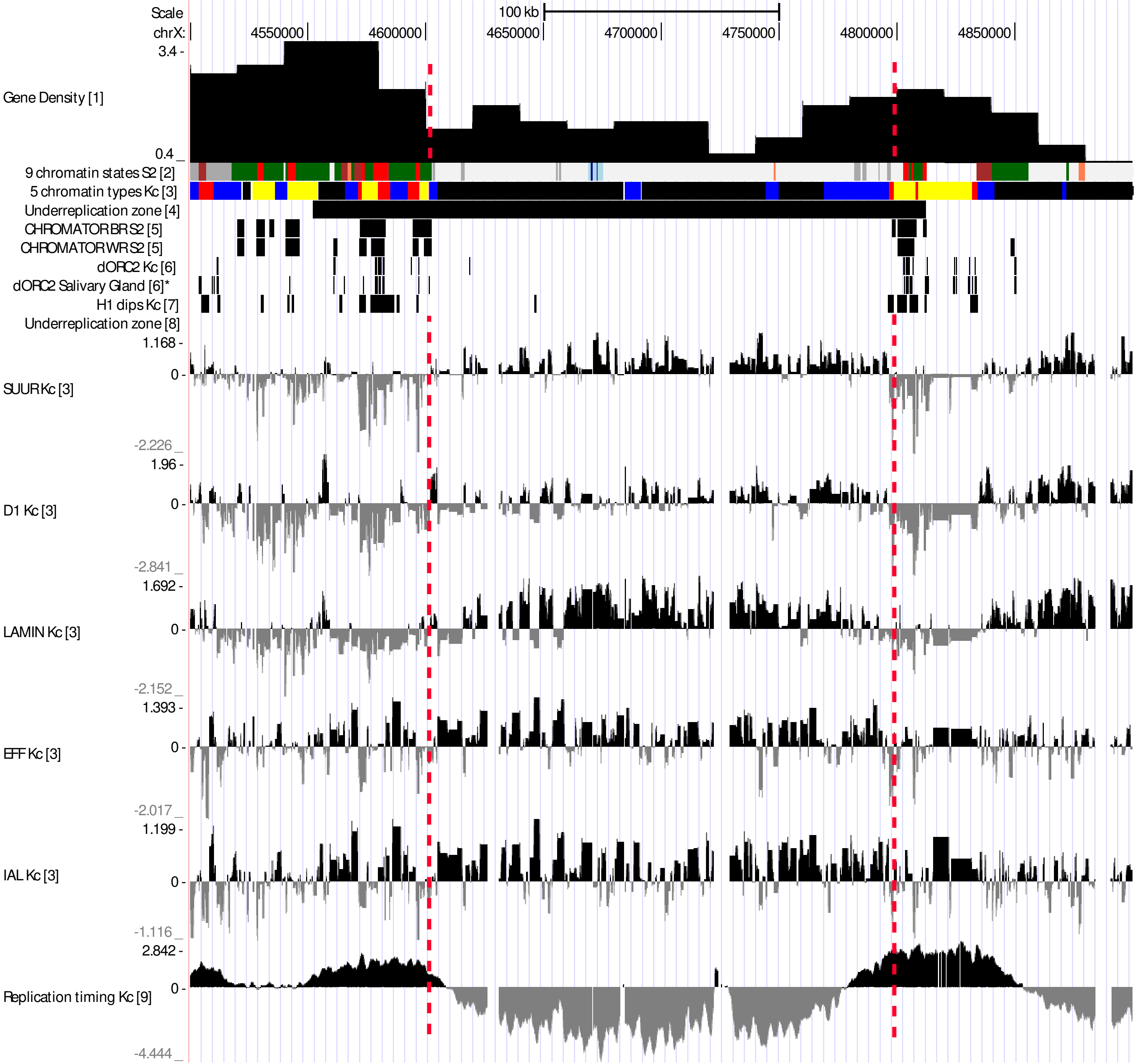
Scale
100 kb
chrX:
4550000
4600000
4650000
4700000
4750000
4800000
4850000
3.4 -
Gene Density [1]
0.4 _
9 chromatin states S2 [2]
5 chromatin types Kc [3]
Underreplication zone [4]
CHROMATOR BR S2 [5]
CHROMATOR WR S2 [5]
dORC2 Kc [6]
dORC2 Salivary Gland [6]*
H1 dips Kc [7]
Underreplication zone [8]
1.168 -
0 -
SUUR Kc [3]
-2.226 _
1.96 -
0 -
D1 Kc [3]
-2.841 _
1.692 -
0 -
LAMIN Kc [3]
-2.152 _
1.393 -
0 -
EFF Kc [3]
-2.017 _
1.199 -
0 -
IAL Kc [3]
-1.116 _
2.842 -
0 -
Replication timing Kc [9]
-4.444 _

7B1-2

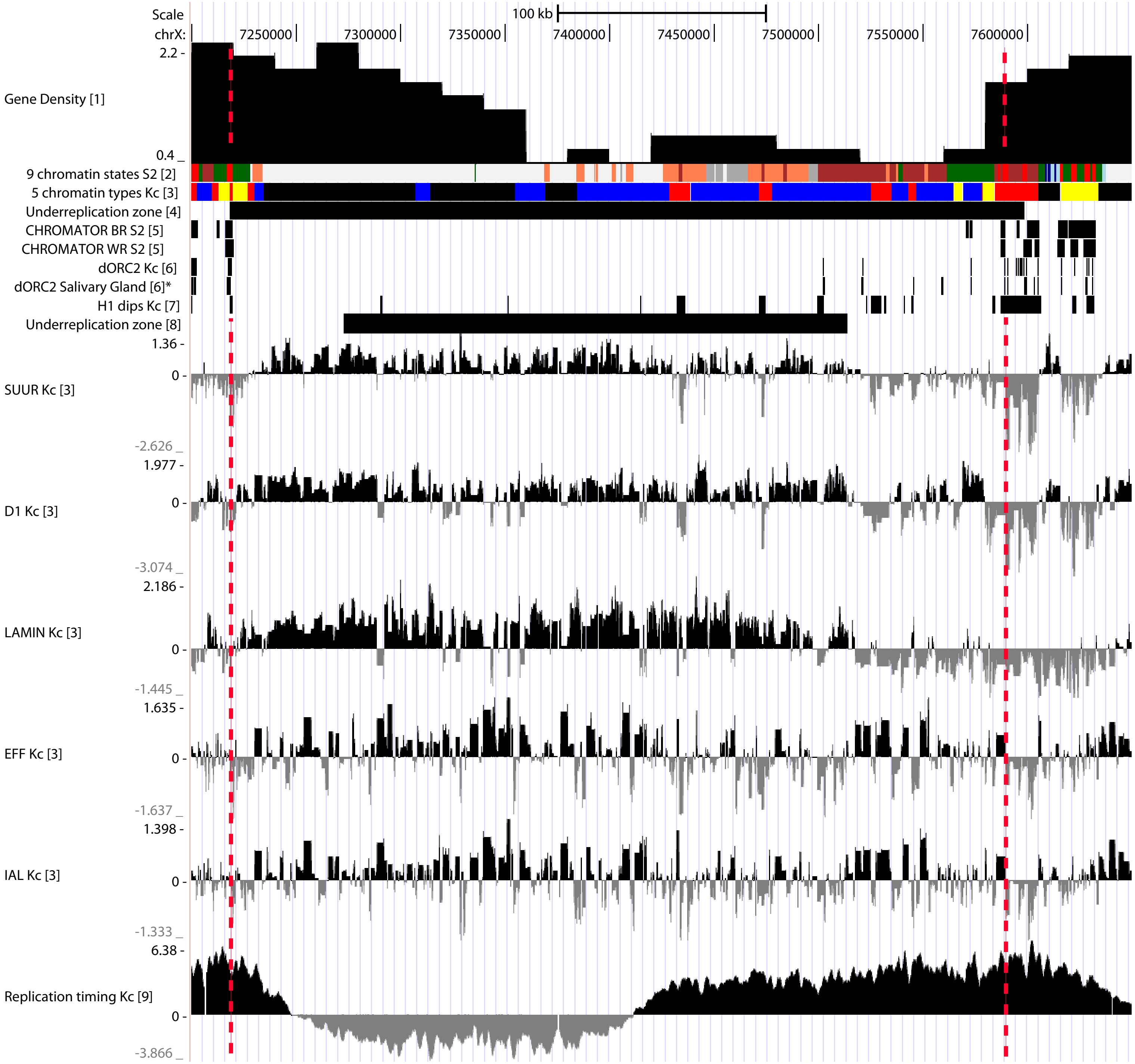
Scale
100 kb
chrX:
7250000
7300000
7350000
7400000
7450000
7500000
7550000
7600000
2.2 -
Gene Density [1]
0.4 _
9 chromatin states S2 [2]
5 chromatin types Kc [3]
Underreplication zone [4]
CHROMATOR BR S2 [5]
CHROMATOR WR S2 [5]
dORC2 Kc [6]
dORC2 Salivary Gland [6]*
H1 dips Kc [7]
Underreplication zone [8]
1.36 -
0 -
SUUR Kc [3]
-2.626 _
1.977 -
0 -
D1 Kc [3]
-3.074 _
2.186 -
LAMIN Kc [3]
0 -
-1.445 _
1.635 -
EFF Kc [3]
0 -
-1.637 _
1.398 -
IAL Kc [3]
0 -
-1.333 _
6.38 -
Replication timing Kc [9]
0 -
-3.866 _

9A3

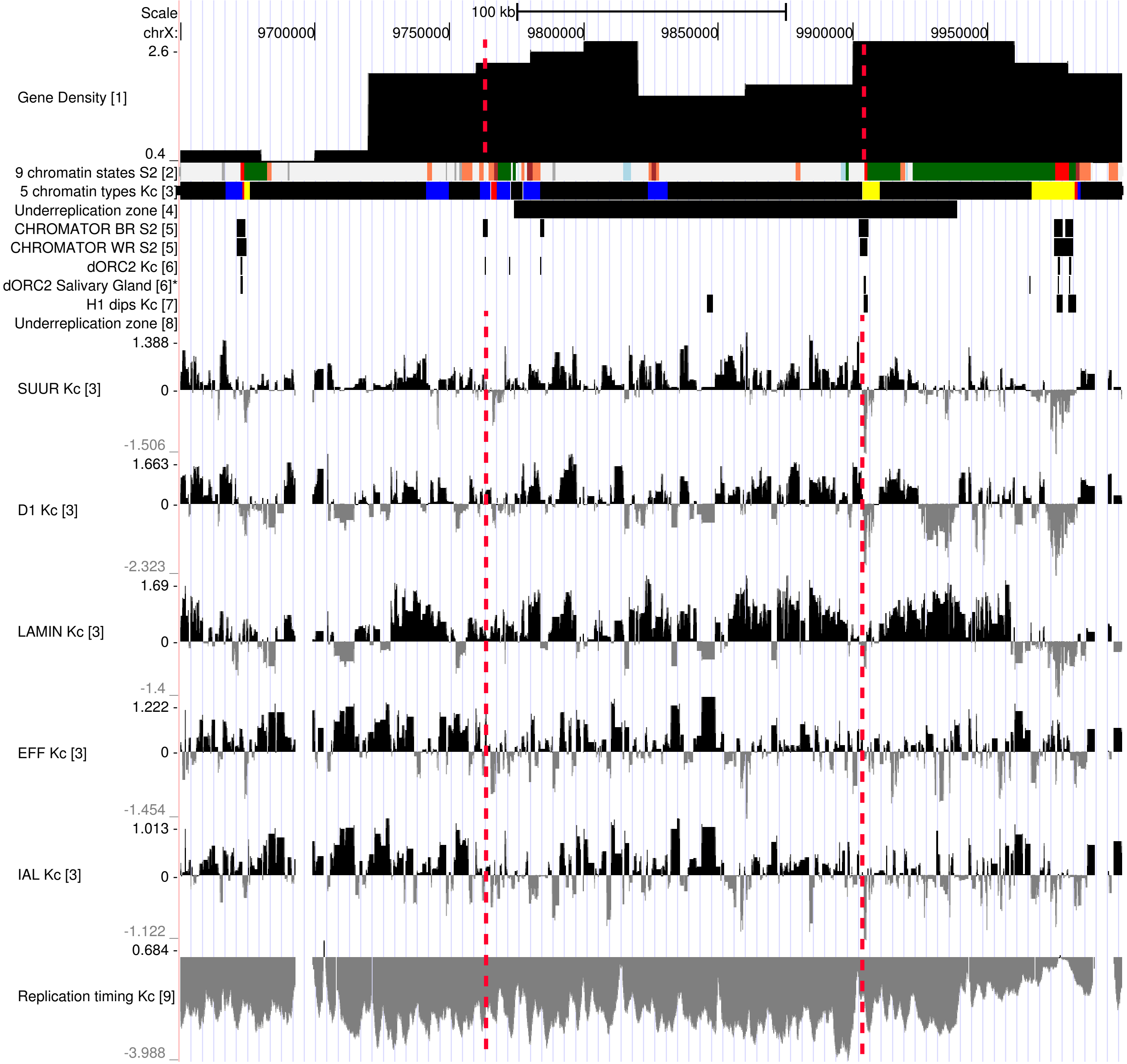
Scale
100 kb
chrX:
9700000
9750000
9800000
9850000
9900000
9950000
2.6 -
Gene Density [1]
0.4 _
9 chromatin states S2 [2]
5 chromatin types Kc [3]
Underreplication zone [4]
CHROMATOR BR S2 [5]
CHROMATOR WR S2 [5]
dORC2 Kc [6]
dORC2 Salivary Gland [6]*
H1 dips Kc [7]
Underreplication zone [8]
1.388 -
SUUR Kc [3]
0 -
-1.506 _
1.663 -
D1 Kc [3]
0 -
-2.323 _
1.69 -
LAMIN Kc [3]
0 -
-1.4 _
1.222 -
EFF Kc [3]
0 -
-1.454 _
1.013 -
IAL Kc [3]
0 -
-1.122 _
0.684 -
Replication timing Kc [9]
-3.988 _

11A6-9

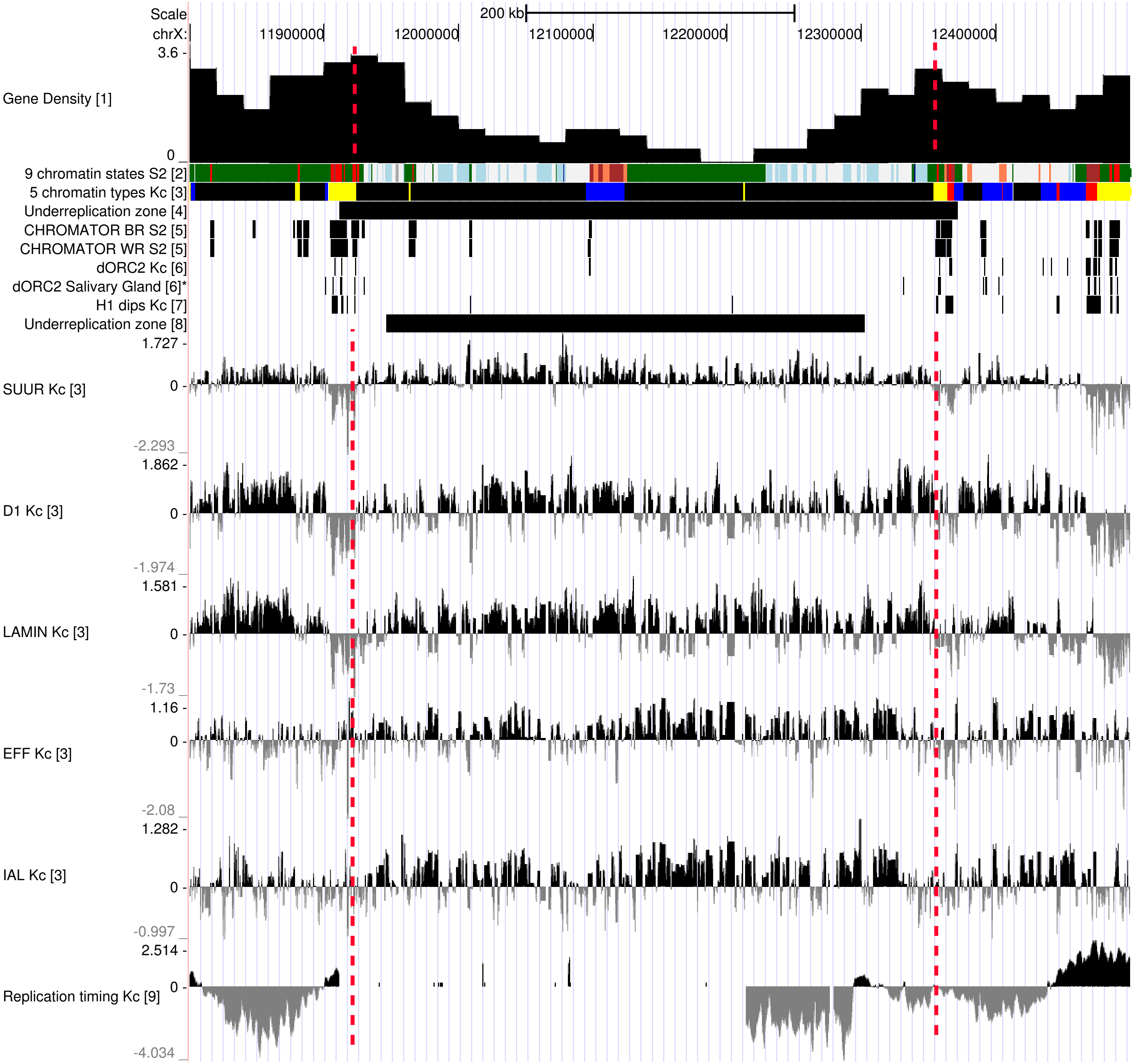

Scale
200 kb
chrX:
11900000
12000000
12100000
12200000
12300000
12400000
3.6
Gene Density [1]
0
9 chromatin states S2 [2]
5 chromatin types Kc [3]
Underreplication zone [4]
CHROMATOR BR S2 [5]
CHROMATOR WR S2 [5]
dORC2 Kc [6]
dORC2 Salivary Gland [6]*
H1 dips Kc [7]
Underreplication zone [8]
1.727
SUUR Kc [3]
0
-2.293
1.862
D1 Kc [3]
0
-1.974
1.581
LAMIN Kc [3]
0
-1.73
1.16
EFF Kc [3]
0
-2.08
1.282
IAL Kc [3]
0
-0.997
2.514
Replication timing Kc [9]
0
-4.034

11D1-2

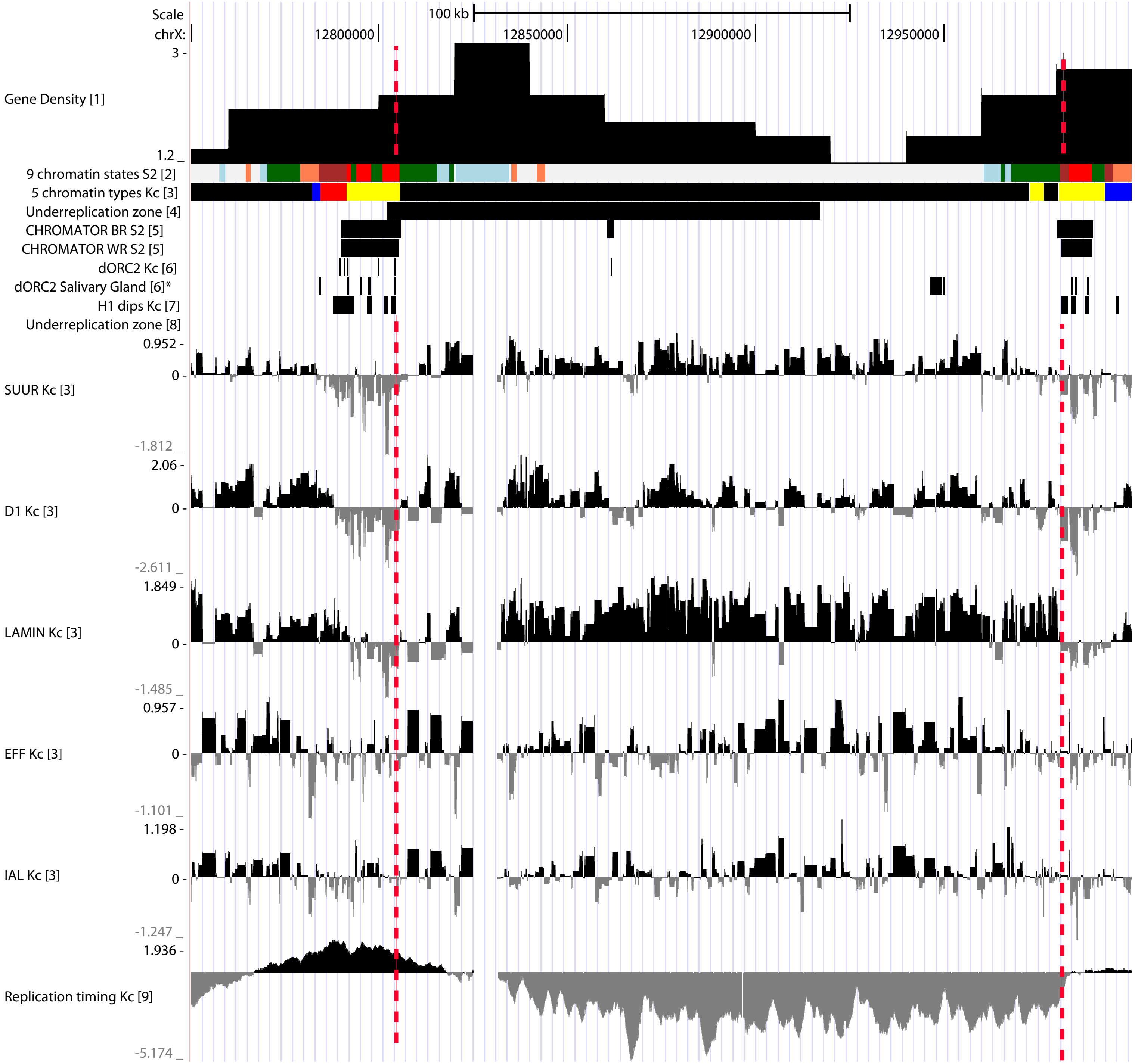
Scale
100 kb
chrX:
12800000
12850000
12900000
12950000
3 -
Gene Density [1]
1.2 _
9 chromatin states S2 [2]
5 chromatin types Kc [3]
Underreplication zone [4]
CHROMATOR BR S2 [5]
CHROMATOR WR S2 [5]
dORC2 Kc [6]
dORC2 Salivary Gland [6]*
H1 dips Kc [7]
Underreplication zone [8]
0.952 -
0 -
SUUR Kc [3]
-1.812 _
2.06 -
0 -
D1 Kc [3]
-2.611 _
1.849 -
LAMIN Kc [3]
0 -
-1.485 _
0.957 -
EFF Kc [3]
0 -
-1.101 _
1.198 -
IAL Kc [3]
0 -
-1.247 _
1.936 -
Replication timing Kc [9]
-5.174 _

12E1-2 and 12E8-9

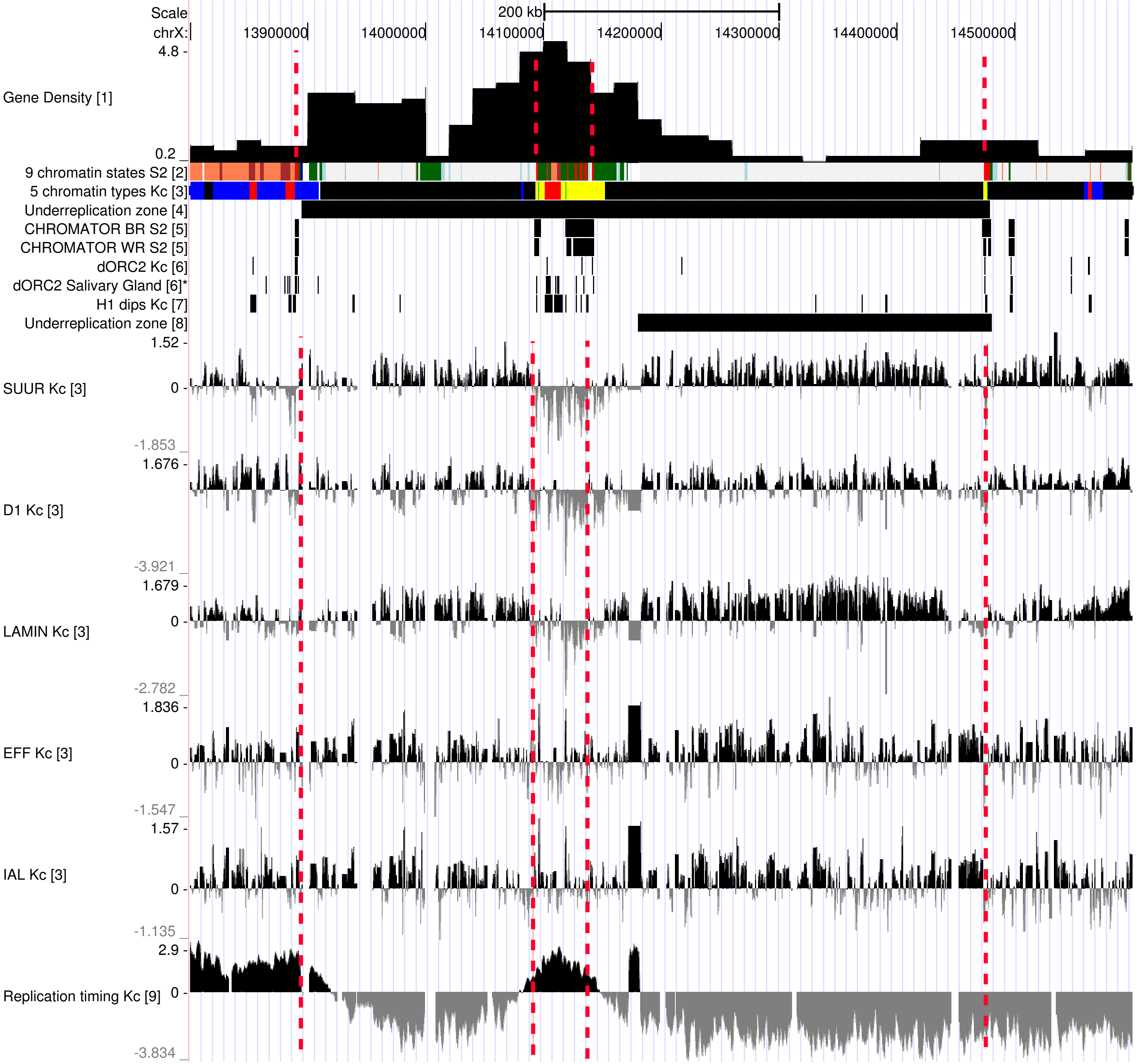

13B3-4

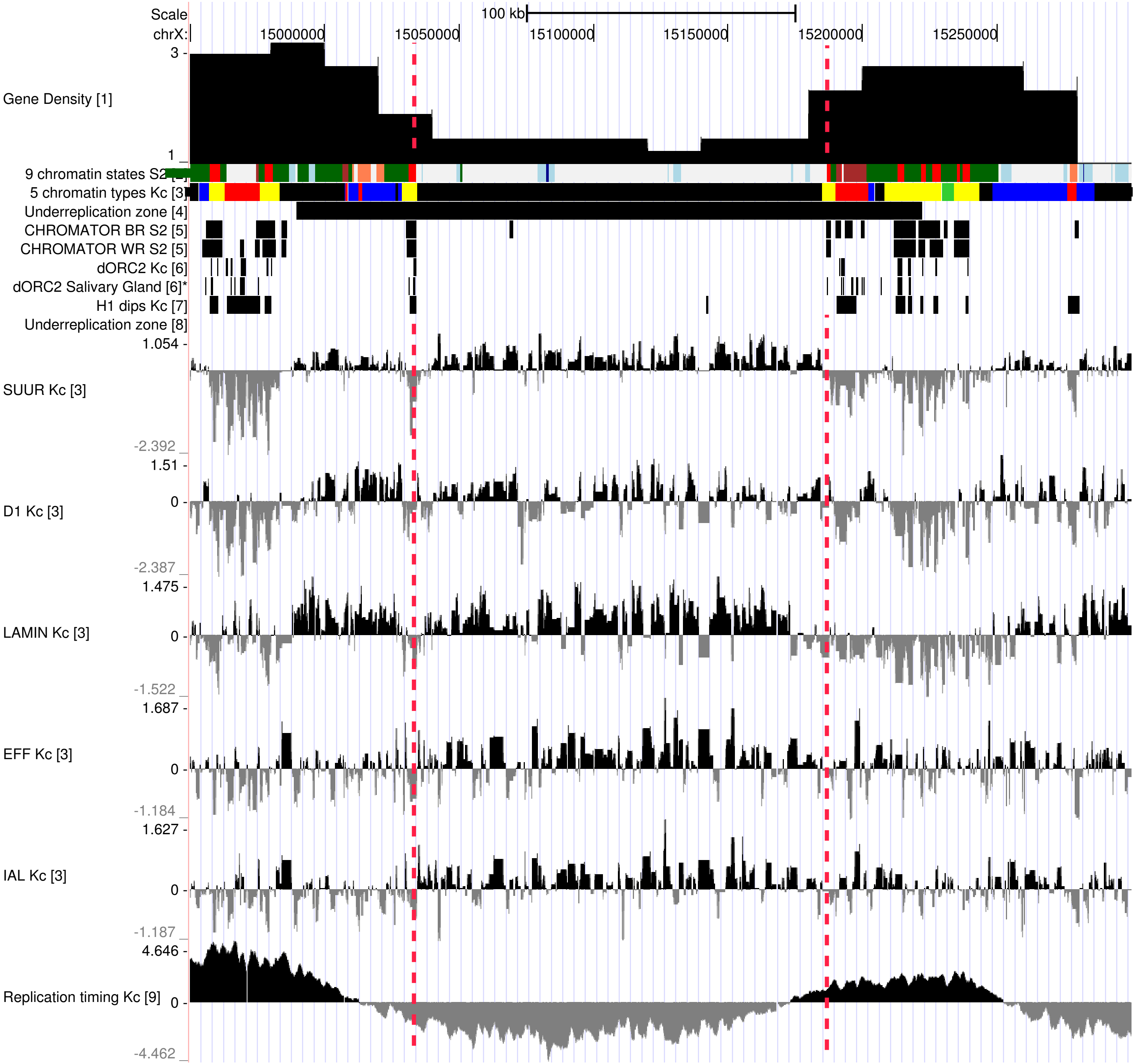
Scale
100 kb
chrX:
15000000
15050000
15100000
15150000
15200000
15250000
3 -
Gene Density [1]
1 _
9 chromatin states S2 [2]
5 chromatin types Kc [3]
Underreplication zone [4]
CHROMATOR BR S2 [5]
CHROMATOR WR S2 [5]
dORC2 Kc [6]
dORC2 Salivary Gland [6]*
H1 dips Kc [7]
Underreplication zone [8]
1.054 -
SUUR Kc [3]
-2.392 _
1.51 -
D1 Kc [3]
0 -
-2.387 _
1.475 -
LAMIN Kc [3]
0 -
-1.522 _
1.687 -
EFF Kc [3]
0 -
-1.184 _
1.627 -
IAL Kc [3]
0 -
-1.187 _
4.646 -
Replication timing Kc [9]
0 -
-4.462 _

19A1-4

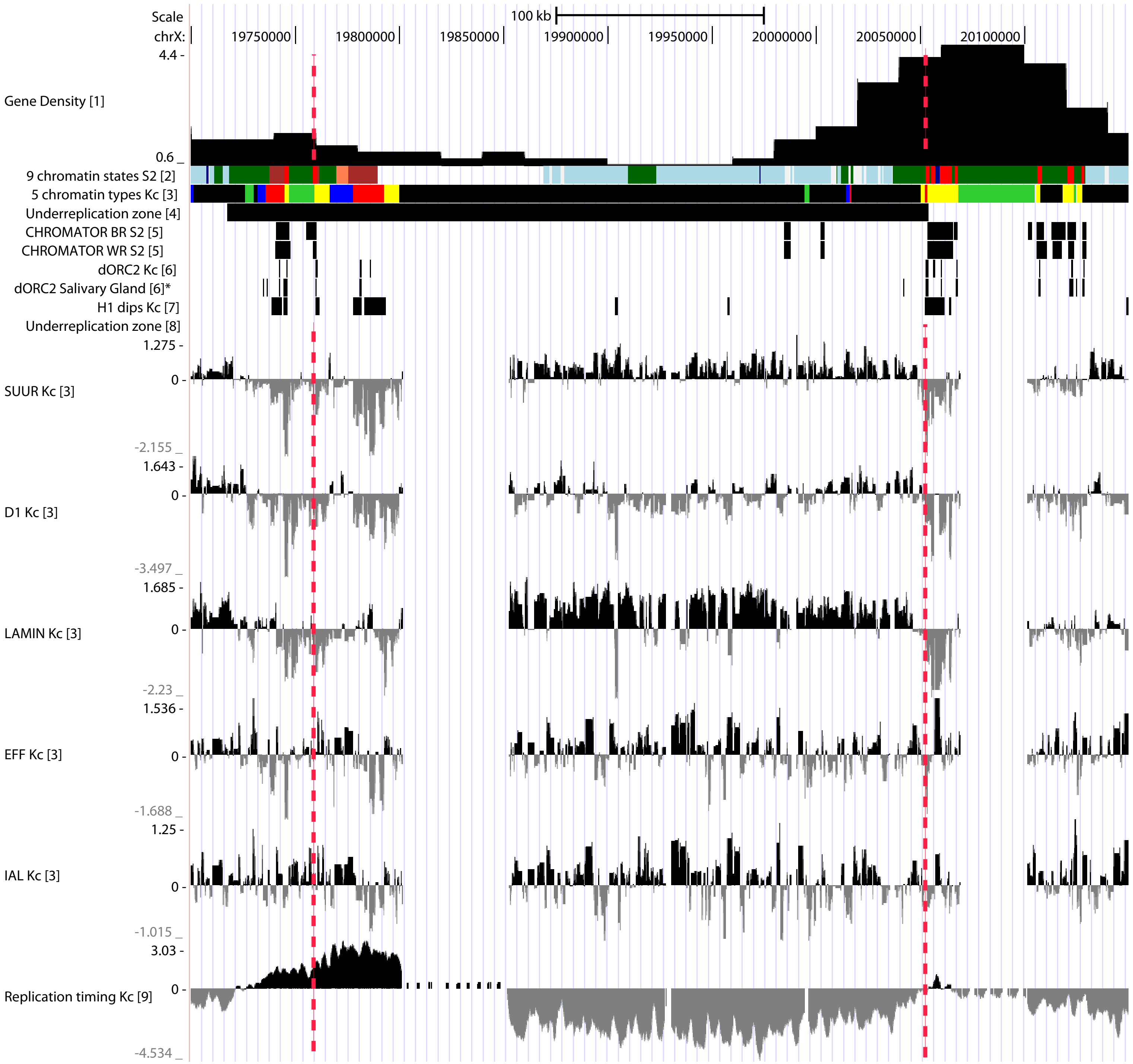
Scale
100 kb
chrX:
19750000
19800000
19850000
19900000
19950000
20000000
20050000
20100000
4.4 -
Gene Density [1]
0.6 _
9 chromatin states S2 [2]
5 chromatin types Kc [3]
Underreplication zone [4]
CHROMATOR BR S2 [5]
CHROMATOR WR S2 [5]
dORC2 Kc [6]
dORC2 Salivary Gland [6]*
H1 dips Kc [7]
Underreplication zone [8]
1.275 -
0 -
SUUR Kc [3]
-2.155 _
1.643 -
0 -
D1 Kc [3]
-3.497 _
1.685 -
0 -
LAMIN Kc [3]
-2.23 _
1.536 -
0 -
EFF Kc [3]
-1.688 _
1.25 -
0 -
IAL Kc [3]
-1.015 _
3.03 -
0 -
Replication timing Kc [9]
-4.534 _

19E1-2 and 19E3-4

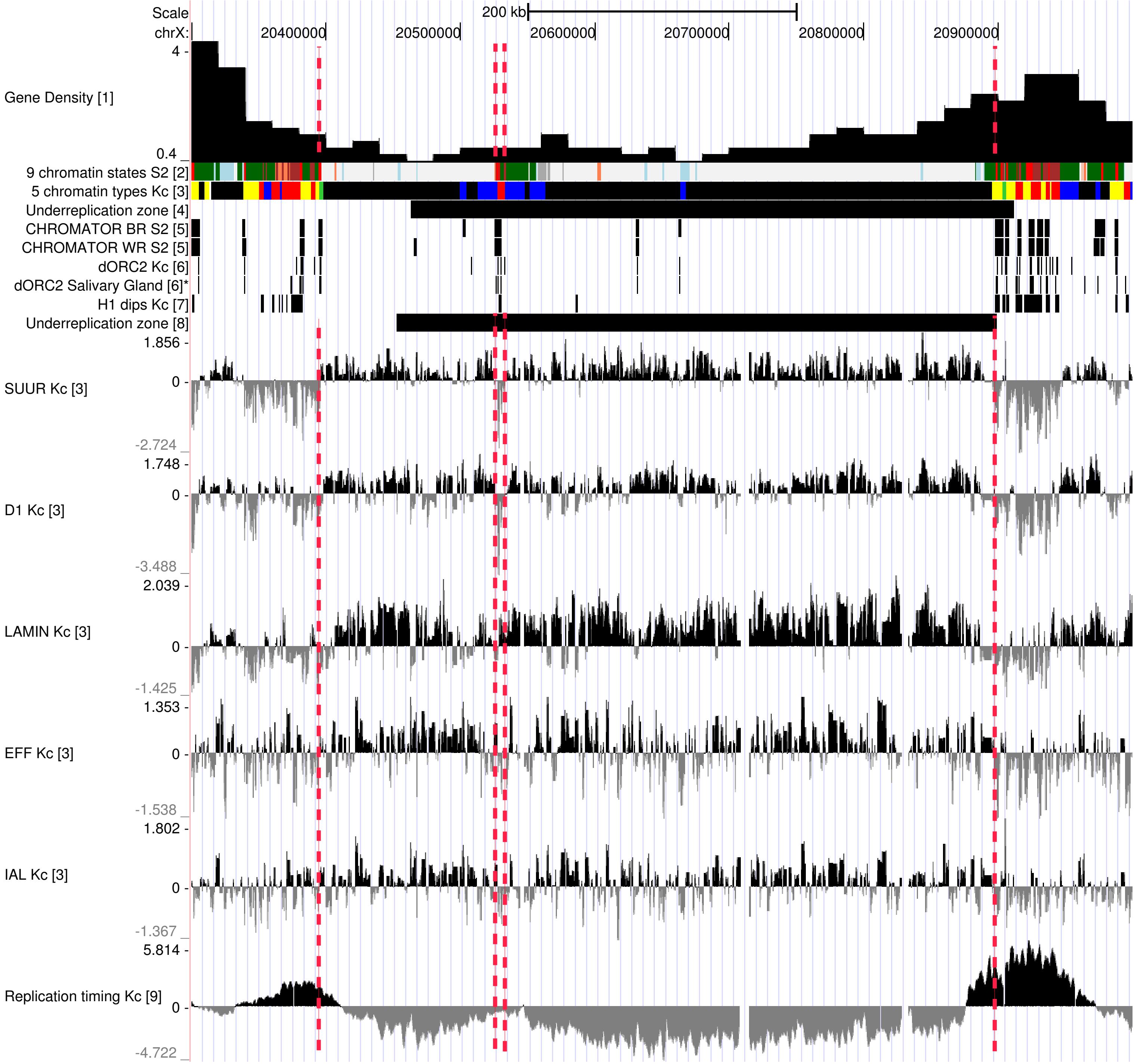

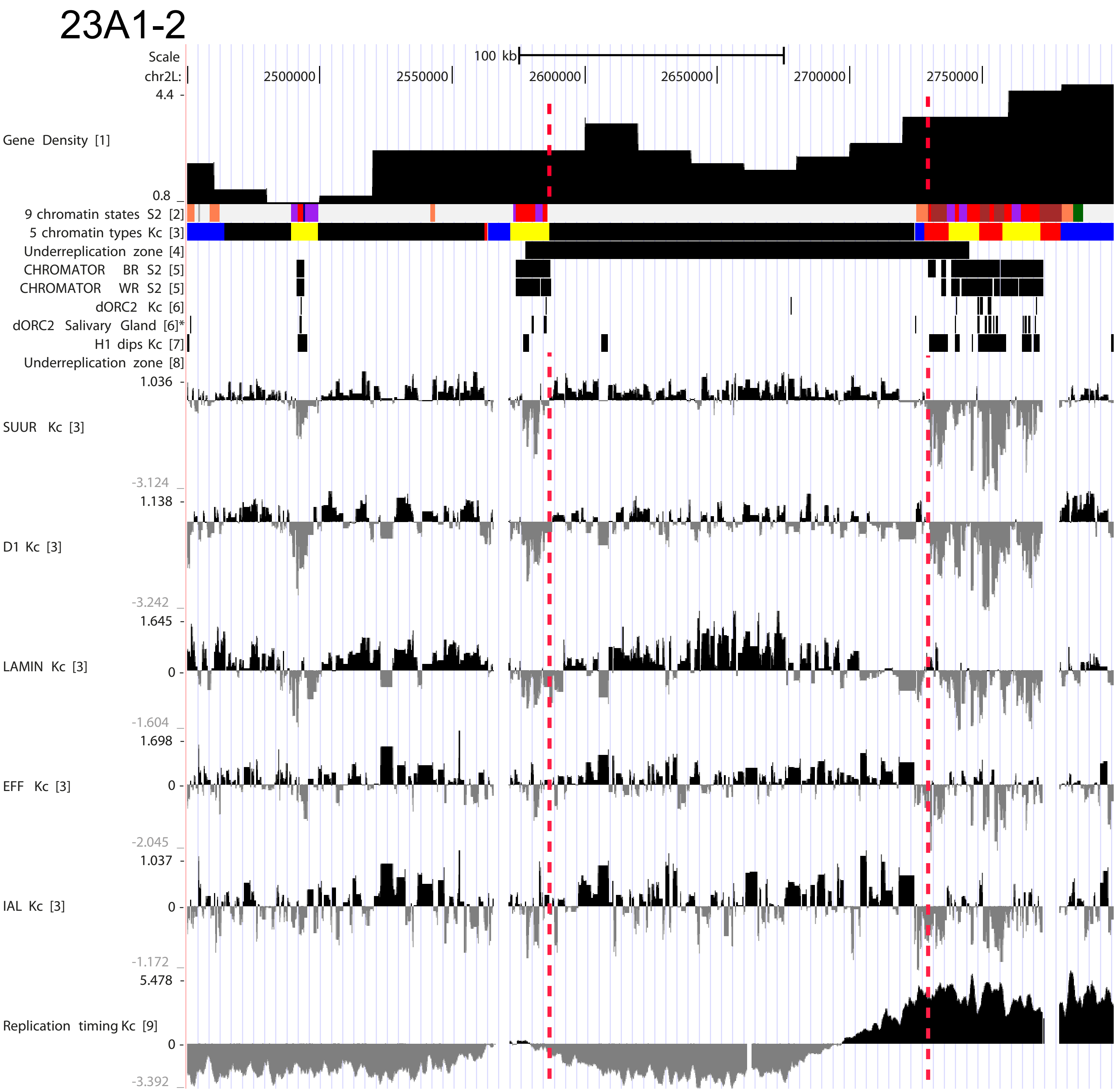

23A1-2
Scale
100 kb
chr2L:
2500000
2550000
2600000
2650000
2700000
2750000
4.4
Gene Density [1]
0.8
9 chromatin states S2 [2]
5 chromatin types Kc [3]
Underreplication zone [4]
CHROMATOR BR S2 [5]
CHROMATOR WR S2 [5]
dORC2 Kc [6]
dORC2 Salivary Gland [6]*
H1 dips Kc [7]
Underreplication zone [8]
1.036
SUUR Kc [3]
-3.124
1.138
D1 Kc [3]
-3.242
1.645
LAMIN Kc [3]
0
-1.604
1.698
EFF Kc [3]
0
-2.045
1.037
IAL Kc [3]
0
-1.172
5.478
Replication timing Kc [9]
0
-3.392

25A1-4

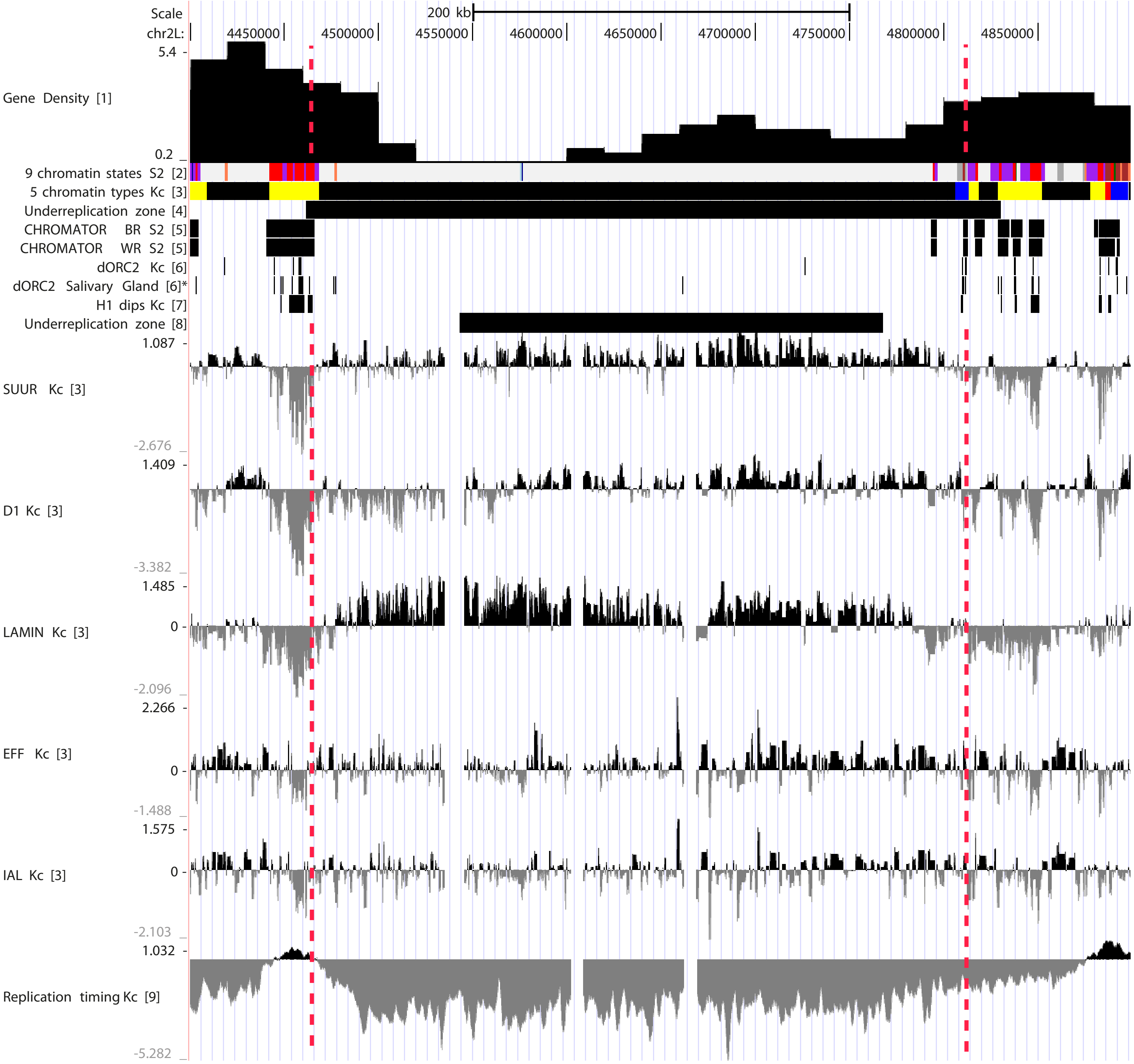

Scale
200 kb
chr2L:
4450000
4500000
4550000
4600000
4650000
4700000
4750000
4800000
4850000
5.4
Gene Density [1]
0.2
9 chromatin states S2 [2]
5 chromatin types Kc [3]
Underreplication zone [4]
CHROMATOR BR S2 [5]
CHROMATOR WR S2 [5]
dORC2 Kc [6]
dORC2 Salivary Gland [6]*
H1 dips Kc [7]
Underreplication zone [8]
1.087
SUUR Kc [3]
-2.676
1.409
D1 Kc [3]
-3.382
1.485
LAMIN Kc [3]
0
-2.096
2.266
EFF Kc [3]
0
-1.488
1.575
IAL Kc [3]
0
-2.103
1.032
Replication timing Kc [9]
-5.282

26C1-2

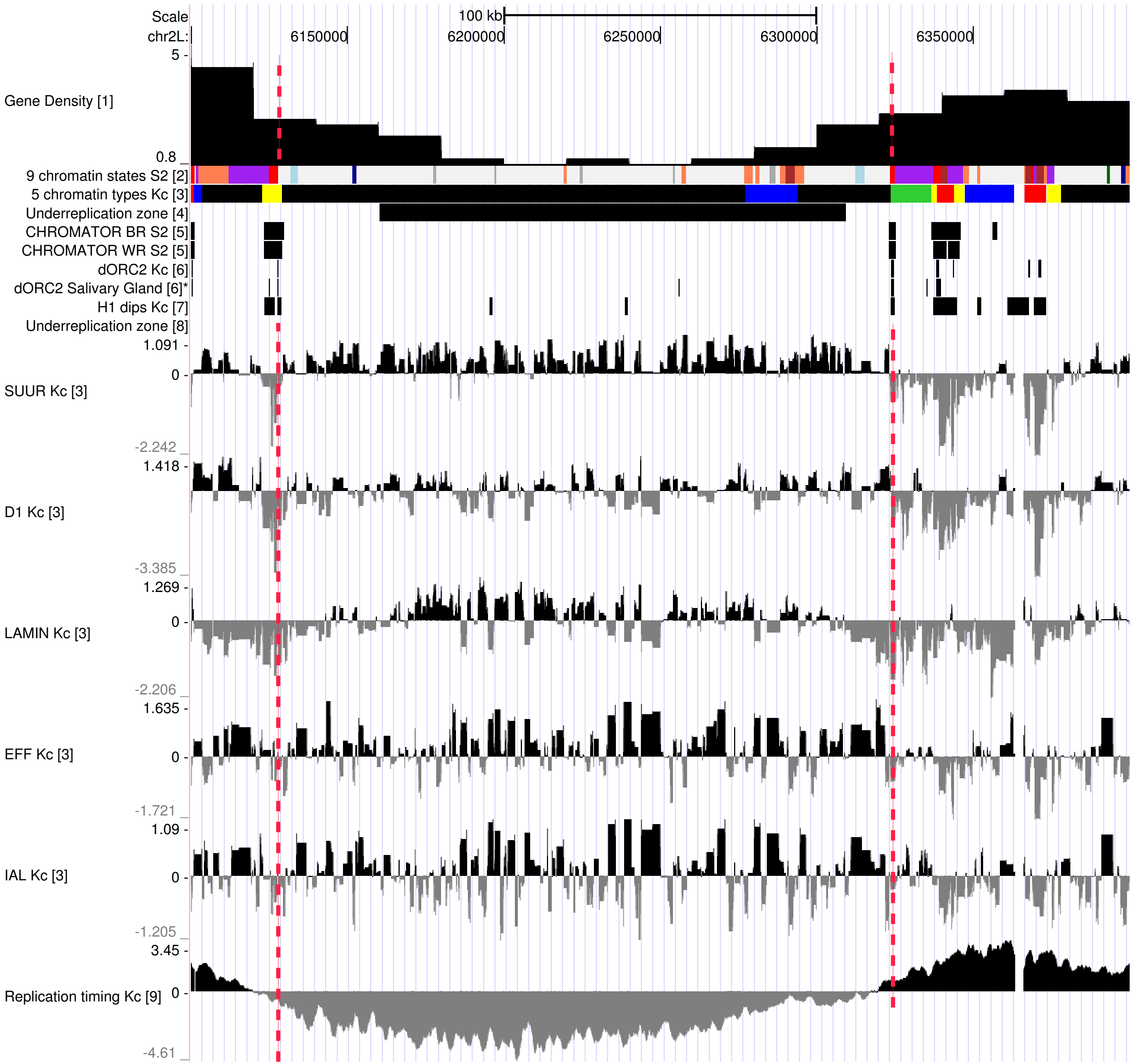
Scale
chr2L:
100 kb
6150000
6200000
6250000
6300000
6350000
Gene Density [1]
5
0.8
9 chromatin states S2 [2]
5 chromatin types Kc [3]
Underreplication zone [4]
CHROMATOR BR S2 [5]
CHROMATOR WR S2 [5]
dORC2 Kc [6]
dORC2 Salivary Gland [6]*
H1 dips Kc [7]
Underreplication zone [8]
SUUR Kc [3]
1.091
0
-2.242
D1 Kc [3]
1.418
-3.385
LAMIN Kc [3]
1.269
0
-2.206
EFF Kc [3]
1.635
0
-1.721
IAL Kc [3]
1.09
0
-1.205
Replication timing Kc [9]
3.45
0
-4.61

32A1-2

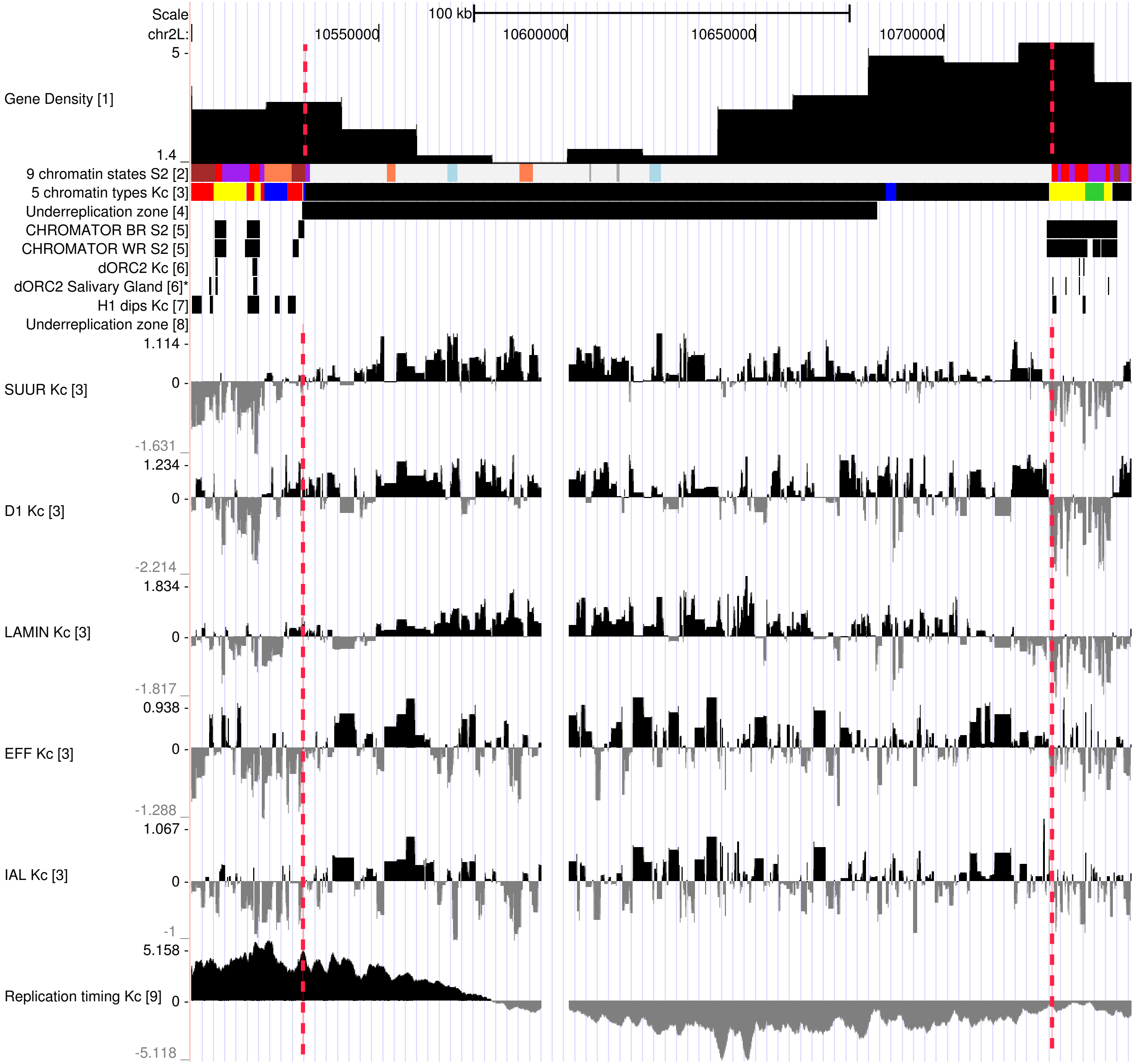
Scale
100 kb
chr2L:
10550000
10600000
10650000
10700000
5
Gene Density [1]
1.4
9 chromatin states S2 [2]
5 chromatin types Kc [3]
Underreplication zone [4]
CHROMATOR BR S2 [5]
CHROMATOR WR S2 [5]
dORC2 Kc [6]
dORC2 Salivary Gland [6]*
H1 dips Kc [7]
Underreplication zone [8]
1.114
SUUR Kc [3]
0
-1.631
1.234
D1 Kc [3]
0
-2.214
1.834
LAMIN Kc [3]
0
-1.817
0.938
EFF Kc [3]
0
-1.288
1.067
IAL Kc [3]
0
-1
5.158
Replication timing Kc [9]
0
-5.118

33A1-2

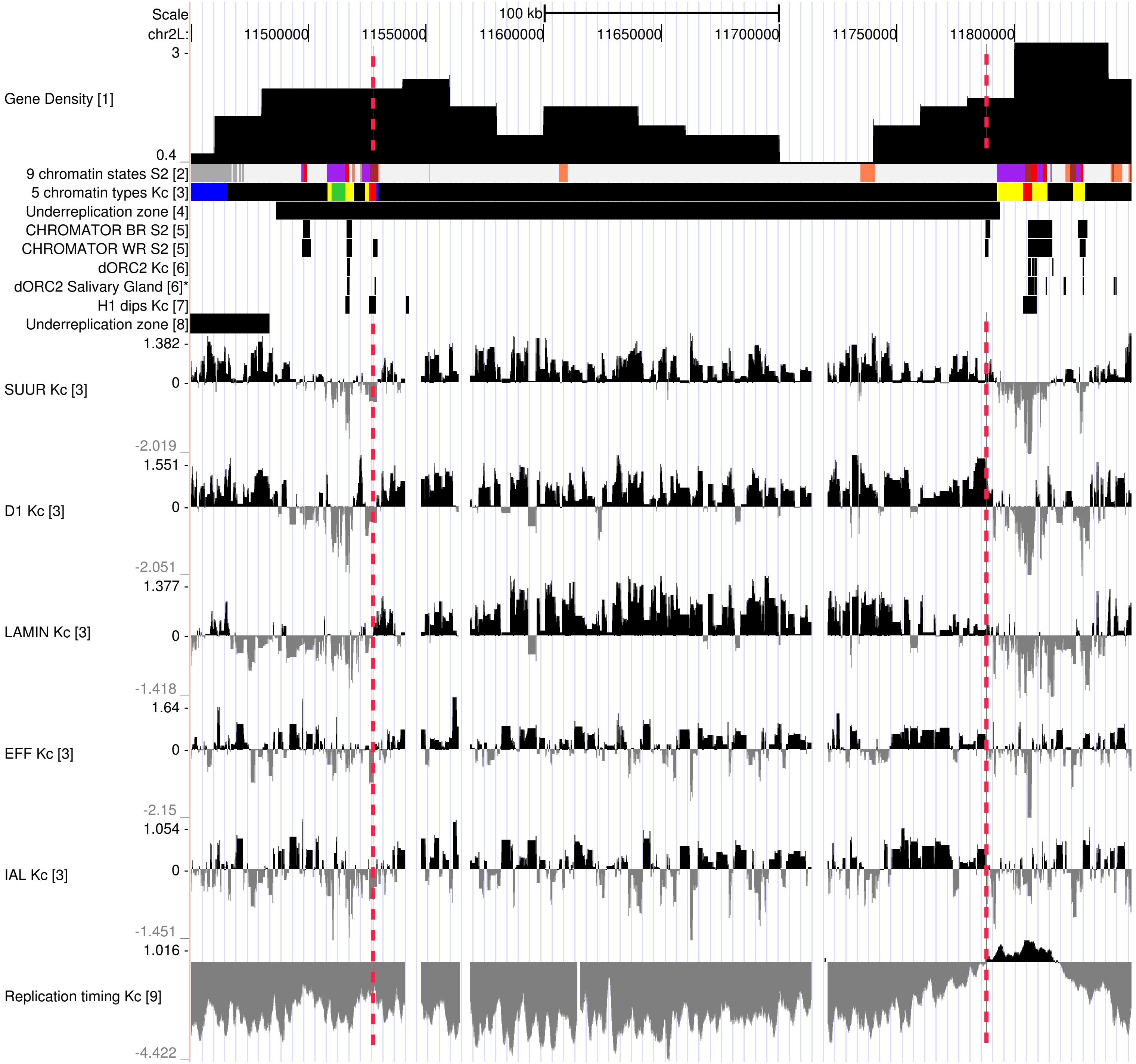
Scale
100 kb
chr2L:
11500000
11550000
11600000
11650000
11700000
11750000
11800000
3 -
Gene Density [1]
0.4 _
9 chromatin states S2 [2]
5 chromatin types Kc [3]
Underreplication zone [4]
CHROMATOR BR S2 [5]
CHROMATOR WR S2 [5]
dORC2 Kc [6]
dORC2 Salivary Gland [6]*
H1 dips Kc [7]
Underreplication zone [8]
1.382 -
0 -
SUUR Kc [3]
-2.019 _
1.551 -
0 -
D1 Kc [3]
-2.051 _
1.377 -
0 -
LAMIN Kc [3]
-1.418 _
1.64 -
0 -
EFF Kc [3]
-2.15 _
1.054 -
0 -
IAL Kc [3]
-1.451 _
1.016 -
Replication timing Kc [9]
-4.422 _

34A1-2

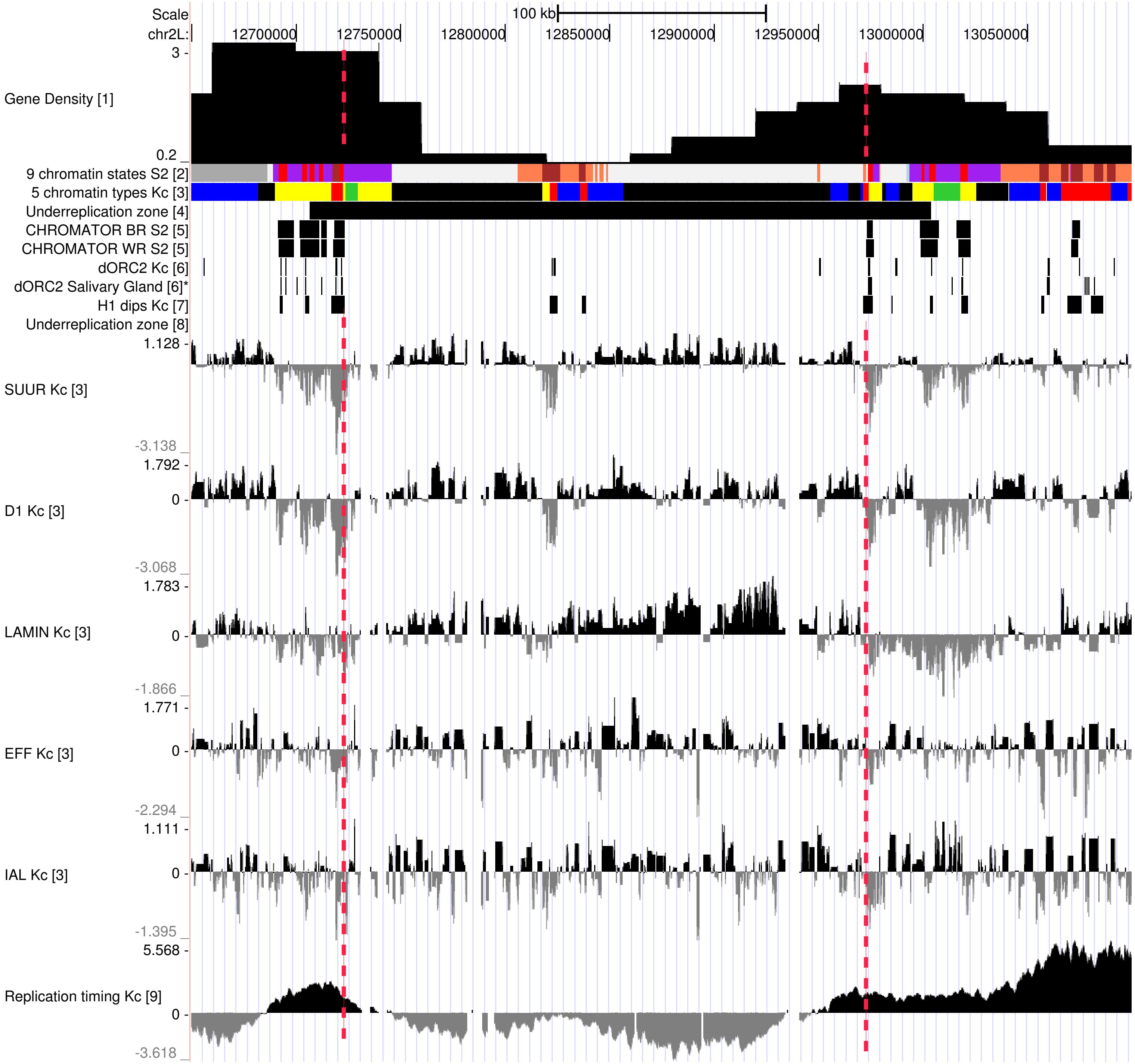

Scale
100 kb
chr2L:
12700000
12750000
12800000
12850000
12900000
12950000
13000000
13050000
3 -
Gene Density [1]
0.2 _
9 chromatin states S2 [2]
5 chromatin types Kc [3]
Underreplication zone [4]
CHROMATOR BR S2 [5]
CHROMATOR WR S2 [5]
dORC2 Kc [6]
dORC2 Salivary Gland [6]*
H1 dips Kc [7]
Underreplication zone [8]
1.128 -
SUUR Kc [3]
-3.138 _
1.792 -
0 -
D1 Kc [3]
-3.068 _
1.783 -
LAMIN Kc [3]
0 -
-1.866 _
1.771 -
EFF Kc [3]
0 -
-2.294 _
1.111 -
IAL Kc [3]
0 -
-1.395 _
5.568 -
Replication timing Kc [9]
0 -
-3.618 _

35B1-2

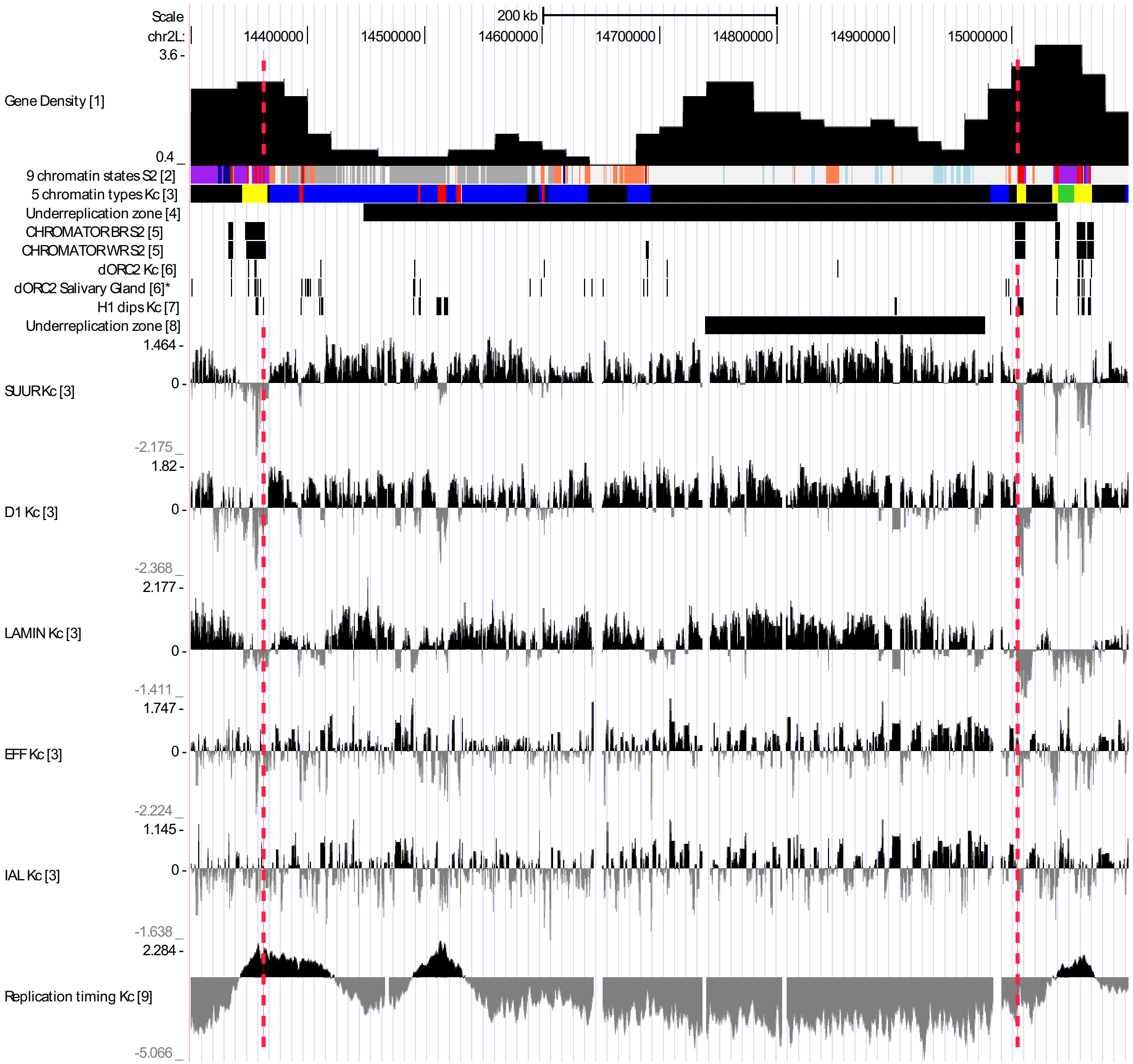
Scale
200 kb
chr2L:
14400000
14500000
14600000
14700000
14800000
14900000
15000000
3.6 -
Gene Density [1]
0.4 _
9 chromatin states S2 [2]
5 chromatin types Kc [3]
Underreplication zone [4]
CHROMATOR BR S2 [5]
CHROMATOR WR S2 [5]
dORC2 Kc [6]
dORC2 Salivary Gland [6]*
H1 dips Kc [7]
Underreplication zone [8]
1.464 -
SUUR Kc [3]
0 -
-2.175 _
1.82 -
D1 Kc [3]
0 -
-2.368 _
2.177 -
LAMIN Kc [3]
0 -
-1.411 _
1.747 -
EFF Kc [3]
0 -
-2.224 _
1.145 -
IAL Kc [3]
0 -
-1.638 _
2.284 -
Replication timing Kc [9]
-5.066 _

35D1-2 and 35D3-4

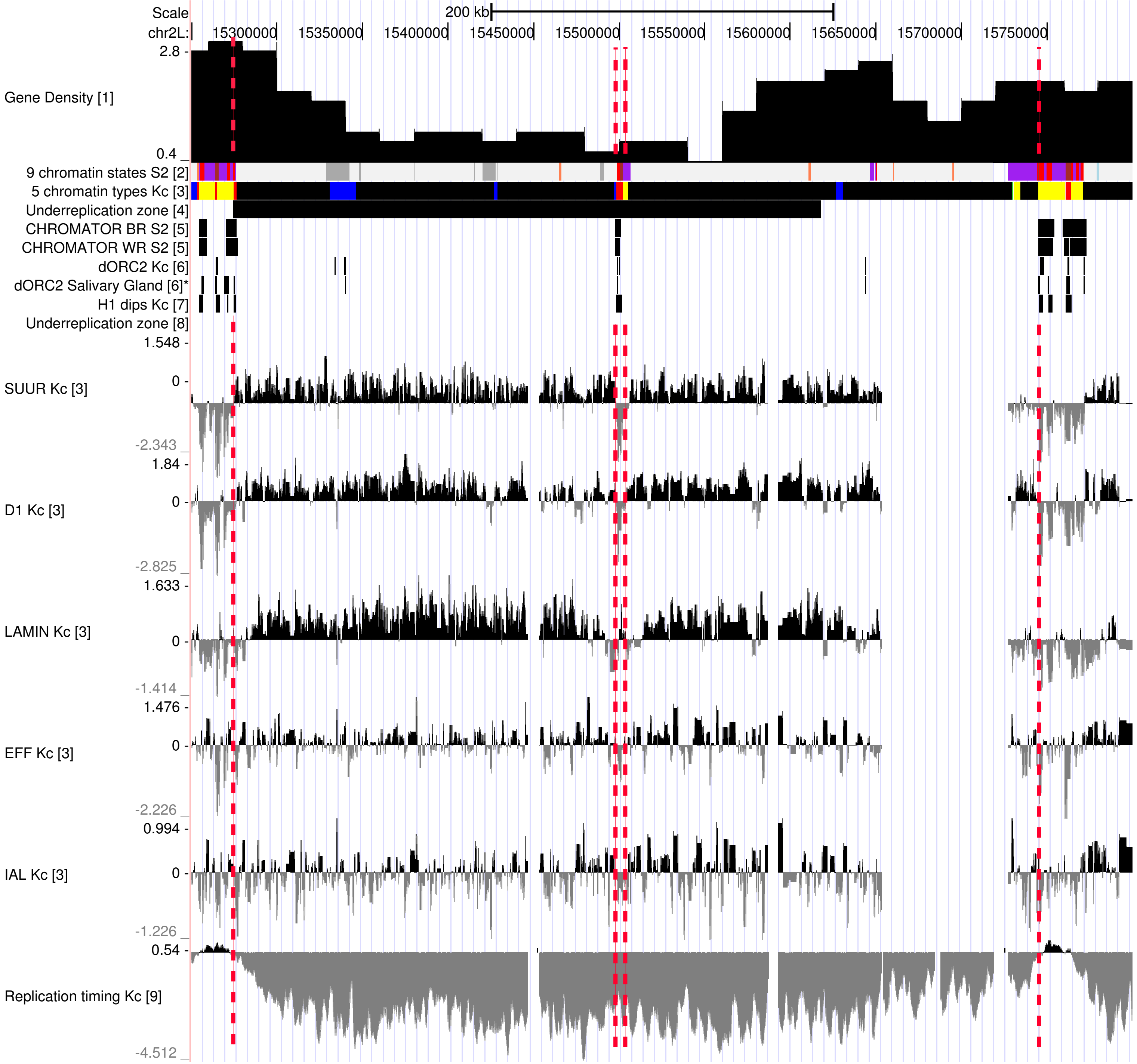

35E1-2

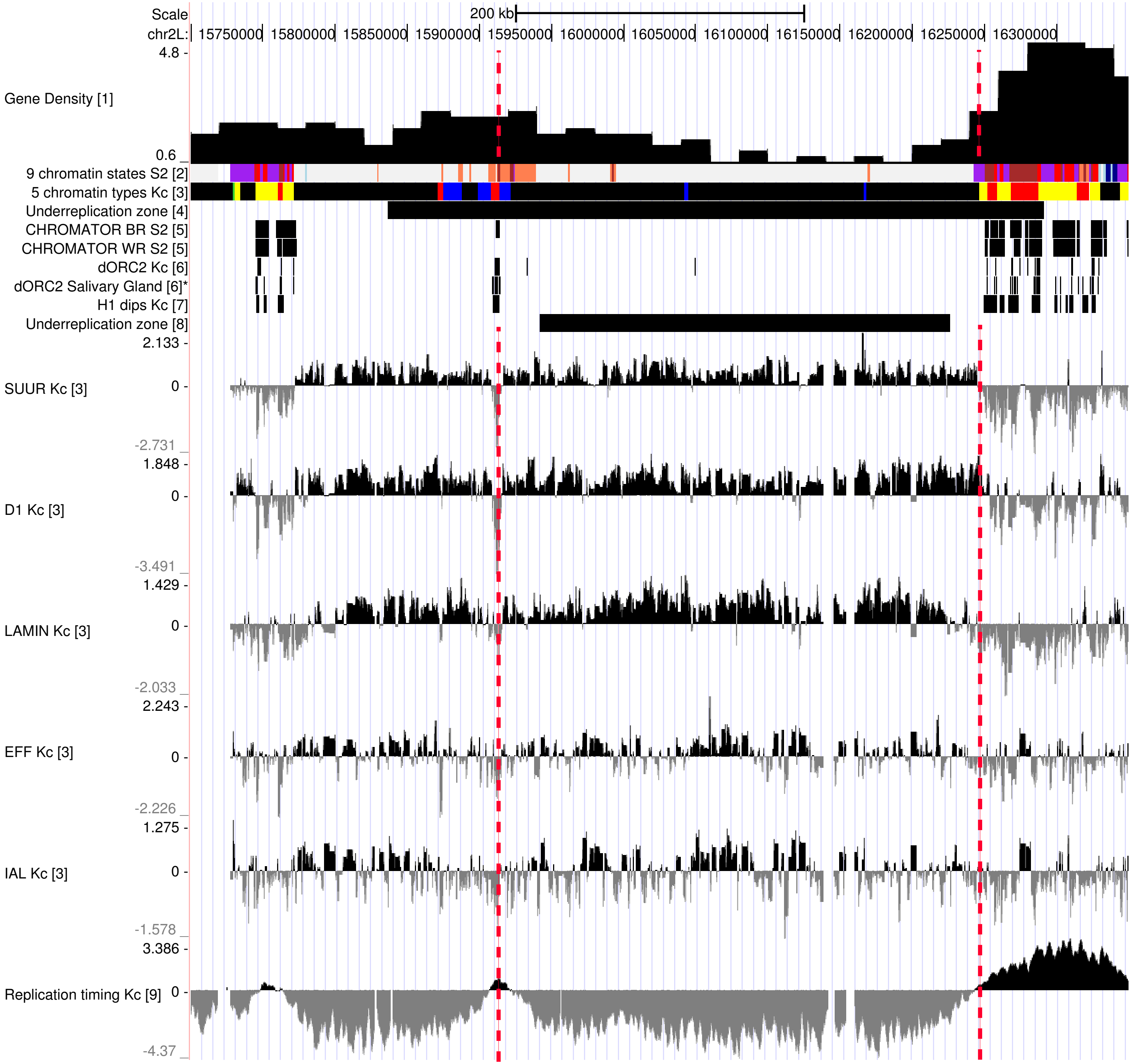
Scale
200 kb
chr2L: 15750000 15800000 15850000 15900000 15950000 16000000 16050000 16100000 16150000 16200000 16250000 16300000
4.8
Gene Density [1]
0.6
9 chromatin states S2 [2]
5 chromatin types Kc [3]
Underreplication zone [4]
CHROMATOR BR S2 [5]
CHROMATOR WR S2 [5]
dORC2 Kc [6]
dORC2 Salivary Gland [6]*
H1 dips Kc [7]
Underreplication zone [8]
2.133
SUUR Kc [3]
0
-2.731
1.848
0
D1 Kc [3]
-3.491
1.429
LAMIN Kc [3]
0
-2.033
2.243
EFF Kc [3]
0
-2.226
1.275
IAL Kc [3]
0
-1.578
3.386
Replication timing Kc [9]
0
-4.37

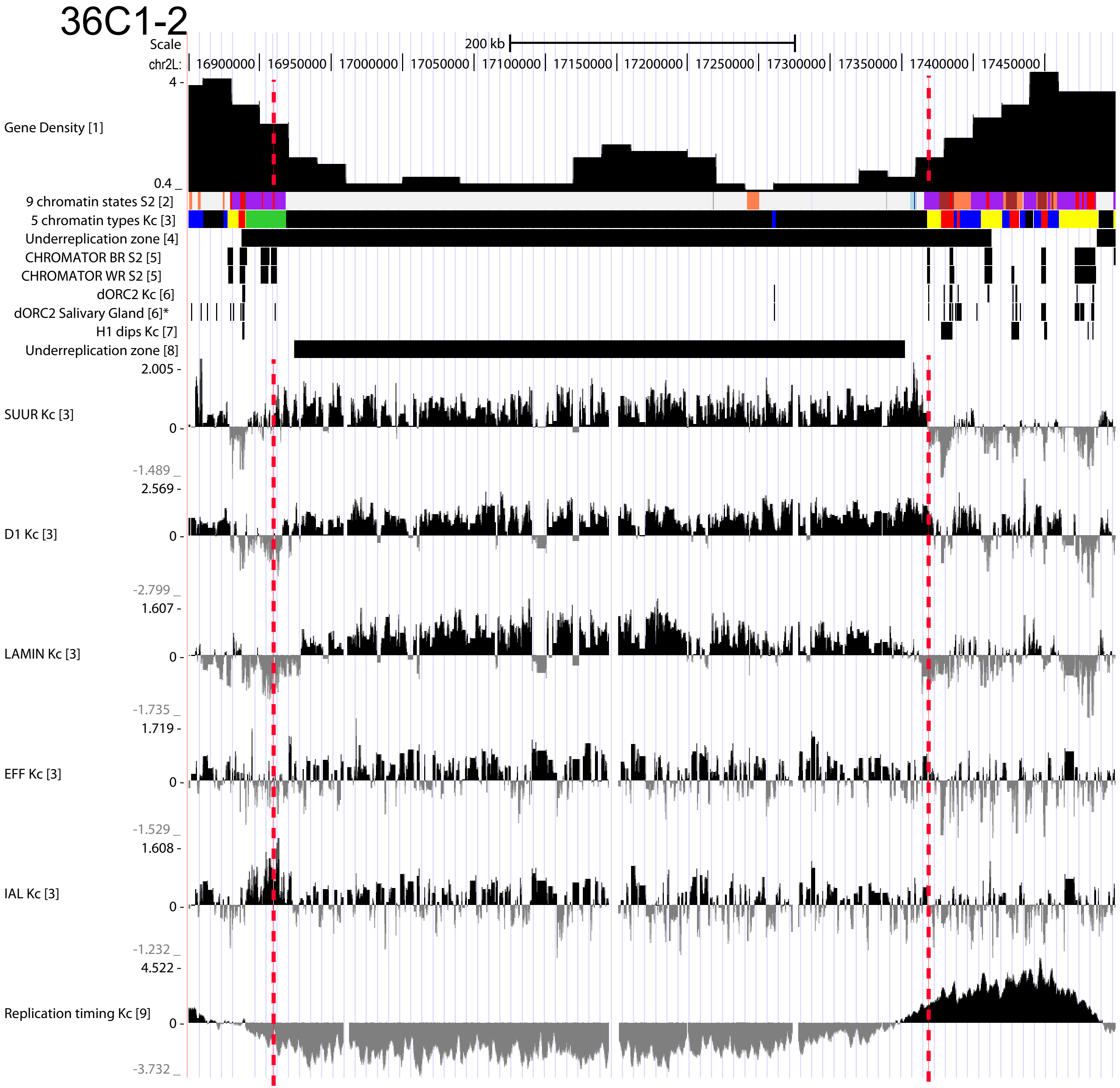
36C1-2
Scale
200 kb
chr2L:
16900000
16950000
17000000
17050000
17100000
17150000
17200000
17250000
17300000
17350000
17400000
17450000
Gene Density [1]
4 -
0.4 _
9 chromatin states S2 [2]
5 chromatin types Kc [3]
Underreplication zone [4]
CHROMATOR BR S2 [5]
CHROMATOR WR S2 [5]
dORC2 Kc [6]
dORC2 Salivary Gland [6]*
H1 dips Kc [7]
Underreplication zone [8]
SUUR Kc [3]
2.005 -
0 -
-1.489 _
D1 Kc [3]
2.569 -
0 -
-2.799 _
LAMIN Kc [3]
1.607 -
0 -
-1.735 _
EFF Kc [3]
1.719 -
0 -
-1.529 _
IAL Kc [3]
1.608 -
0 -
-1.232 _
Replication timing Kc [9]
4.522 -
0 -
-3.732 _

36D1-4

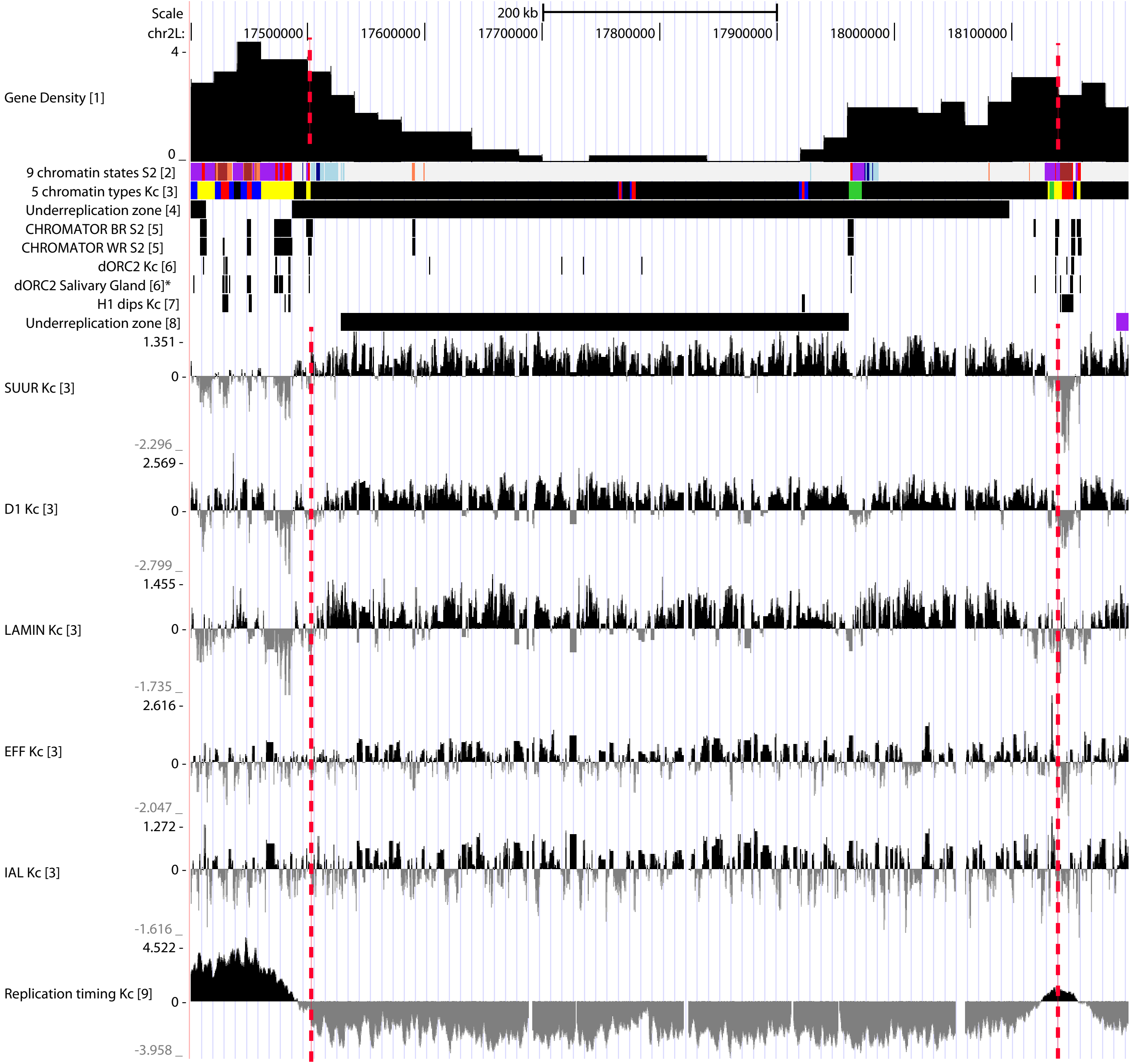
Scale
200 kb
chr2L:
17500000
17600000
17700000
17800000
17900000
18000000
18100000
4 -
Gene Density [1]
0 _
9 chromatin states S2 [2]
5 chromatin types Kc [3]
Underreplication zone [4]
CHROMATOR BR S2 [5]
CHROMATOR WR S2 [5]
dORC2 Kc [6]
dORC2 Salivary Gland [6]*
H1 dips Kc [7]
Underreplication zone [8]
1.351 -
0 -
SUUR Kc [3]
-2.296 _
2.569 -
D1 Kc [3]
0 -
-2.799 _
1.455 -
LAMIN Kc [3]
0 -
-1.735 _
2.616 -
EFF Kc [3]
0 -
-2.047 _
1.272 -
IAL Kc [3]
0 -
-1.616 _
4.522 -
Replication timing Kc [9]
0 -
-3.958 _

47A1-2

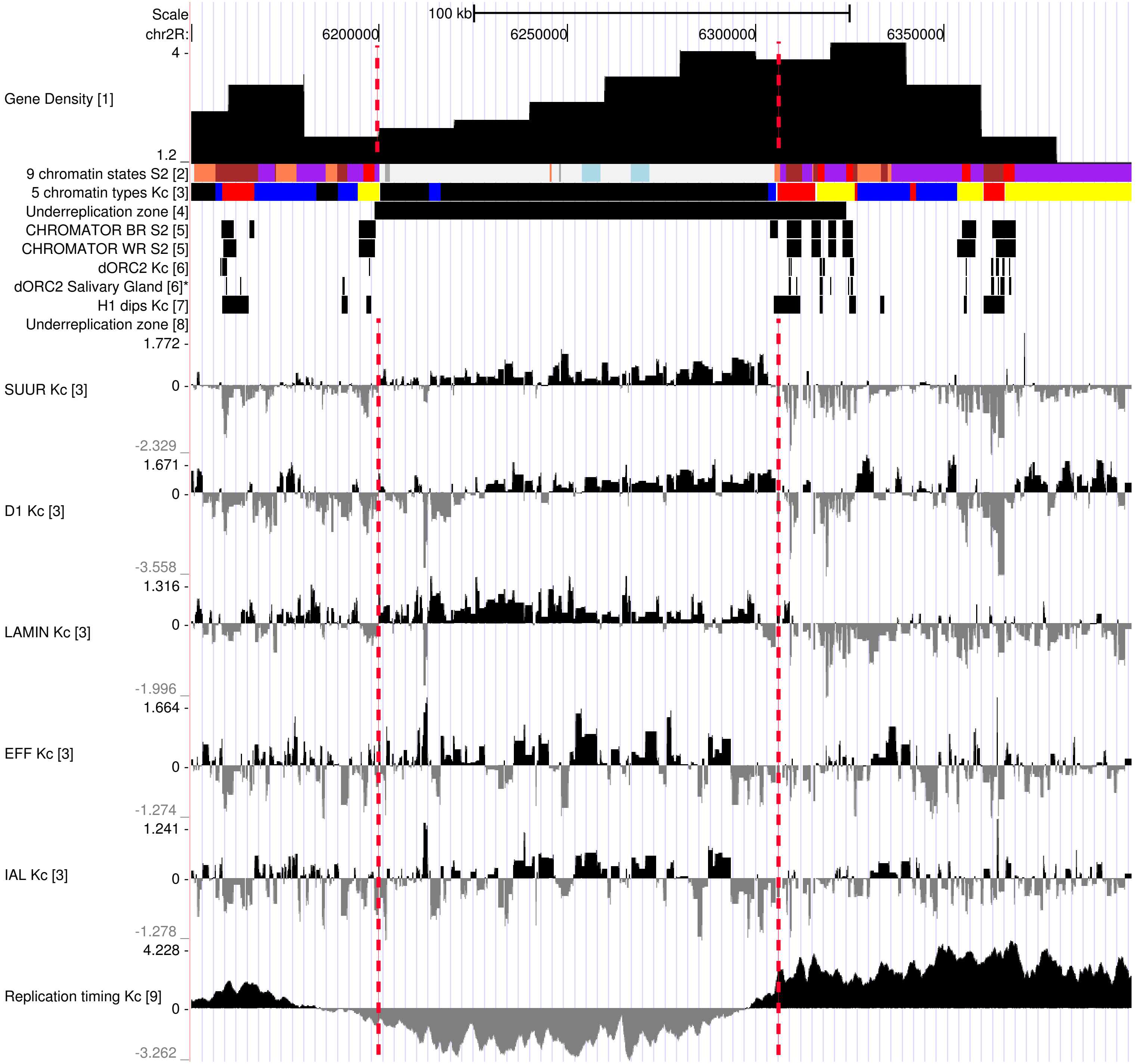

Scale
100 kb
chr2R:
6200000
6250000
6300000
6350000
4 -
Gene Density [1]
1.2 _
9 chromatin states S2 [2]
5 chromatin types Kc [3]
Underreplication zone [4]
CHROMATOR BR S2 [5]
CHROMATOR WR S2 [5]
dORC2 Kc [6]
dORC2 Salivary Gland [6]*
H1 dips Kc [7]
Underreplication zone [8]
1.772 -
SUUR Kc [3]
0 -
-2.329 _
1.671 -
0 -
D1 Kc [3]
-3.558 _
1.316 -
0 -
LAMIN Kc [3]
-1.996 _
1.664 -
EFF Kc [3]
0 -
-1.274 _
1.241 -
IAL Kc [3]
0 -
-1.278 _
4.228 -
Replication timing Kc [9]
0 -
-3.262 _

50C1-4

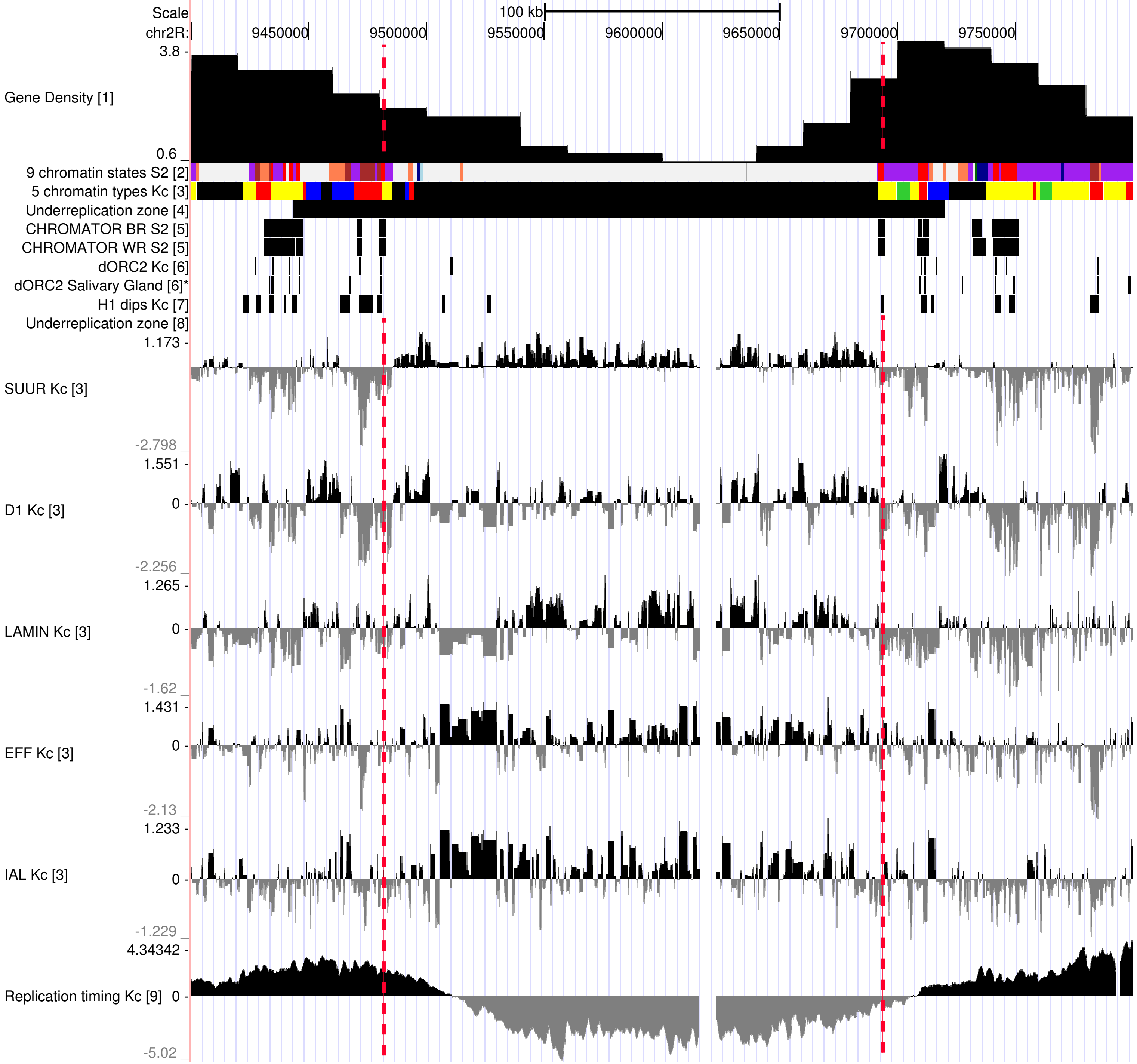
Scale
100 kb
chr2R:
9450000
9500000
9550000
9600000
9650000
9700000
9750000
3.8
Gene Density [1]
0.6
9 chromatin states S2 [2]
5 chromatin types Kc [3]
Underreplication zone [4]
CHROMATOR BR S2 [5]
CHROMATOR WR S2 [5]
dORC2 Kc [6]
dORC2 Salivary Gland [6]*
H1 dips Kc [7]
Underreplication zone [8]
1.173
SUUR Kc [3]
-2.798
1.551
D1 Kc [3]
0
-2.256
1.265
LAMIN Kc [3]
0
-1.62
1.431
EFF Kc [3]
0
-2.13
1.233
IAL Kc [3]
0
-1.229
4.34342
Replication timing Kc [9]
0
-5.02

53C1-2

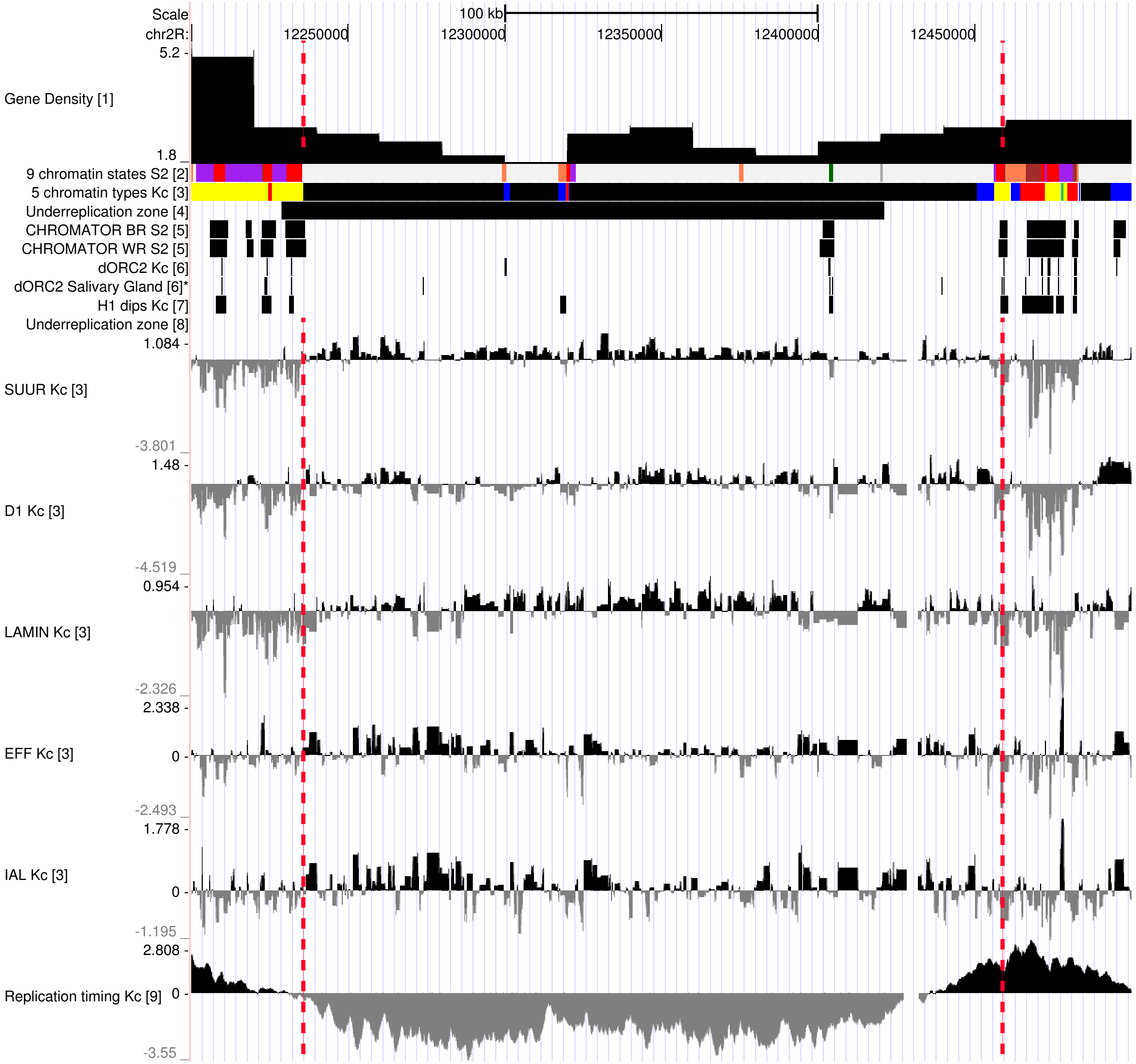

Scale
100 kb
chr2R:
12250000
12300000
12350000
12400000
12450000
5.2 -
Gene Density [1]
1.8 _
9 chromatin states S2 [2]
5 chromatin types Kc [3]
Underreplication zone [4]
CHROMATOR BR S2 [5]
CHROMATOR WR S2 [5]
dORC2 Kc [6]
dORC2 Salivary Gland [6]*
H1 dips Kc [7]
Underreplication zone [8]
1.084 -
SUUR Kc [3]
-3.801 _
1.48 -
D1 Kc [3]
-4.519 _
0.954 -
LAMIN Kc [3]
-2.326 _
2.338 -
EFF Kc [3]
0 -
-2.493 _
1.778 -
IAL Kc [3]
0 -
-1.195 _
2.808 -
Replication timing Kc [9]
0 -
-3.55 _

56A1-2 and 56B1-2

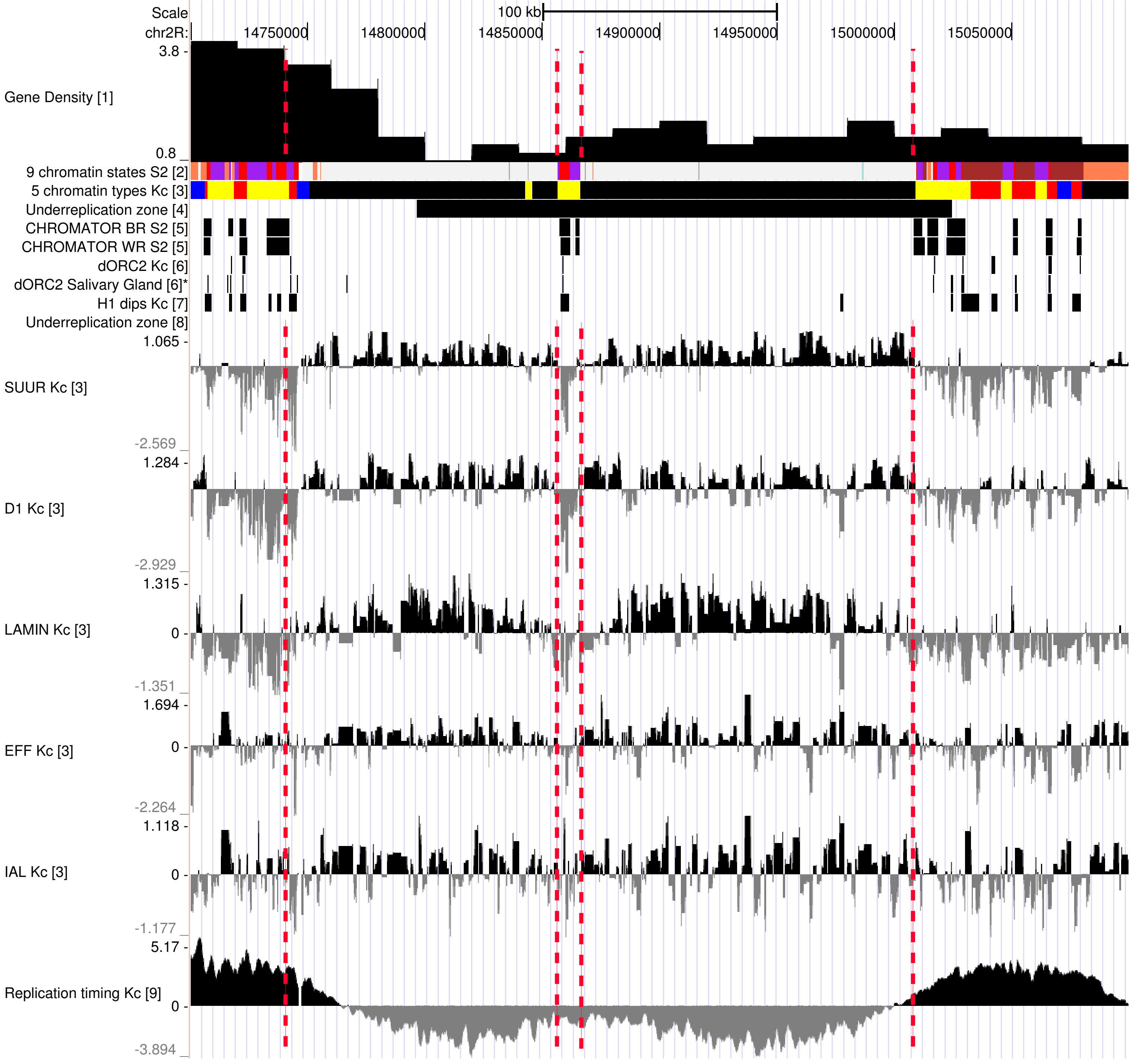

57A1-4

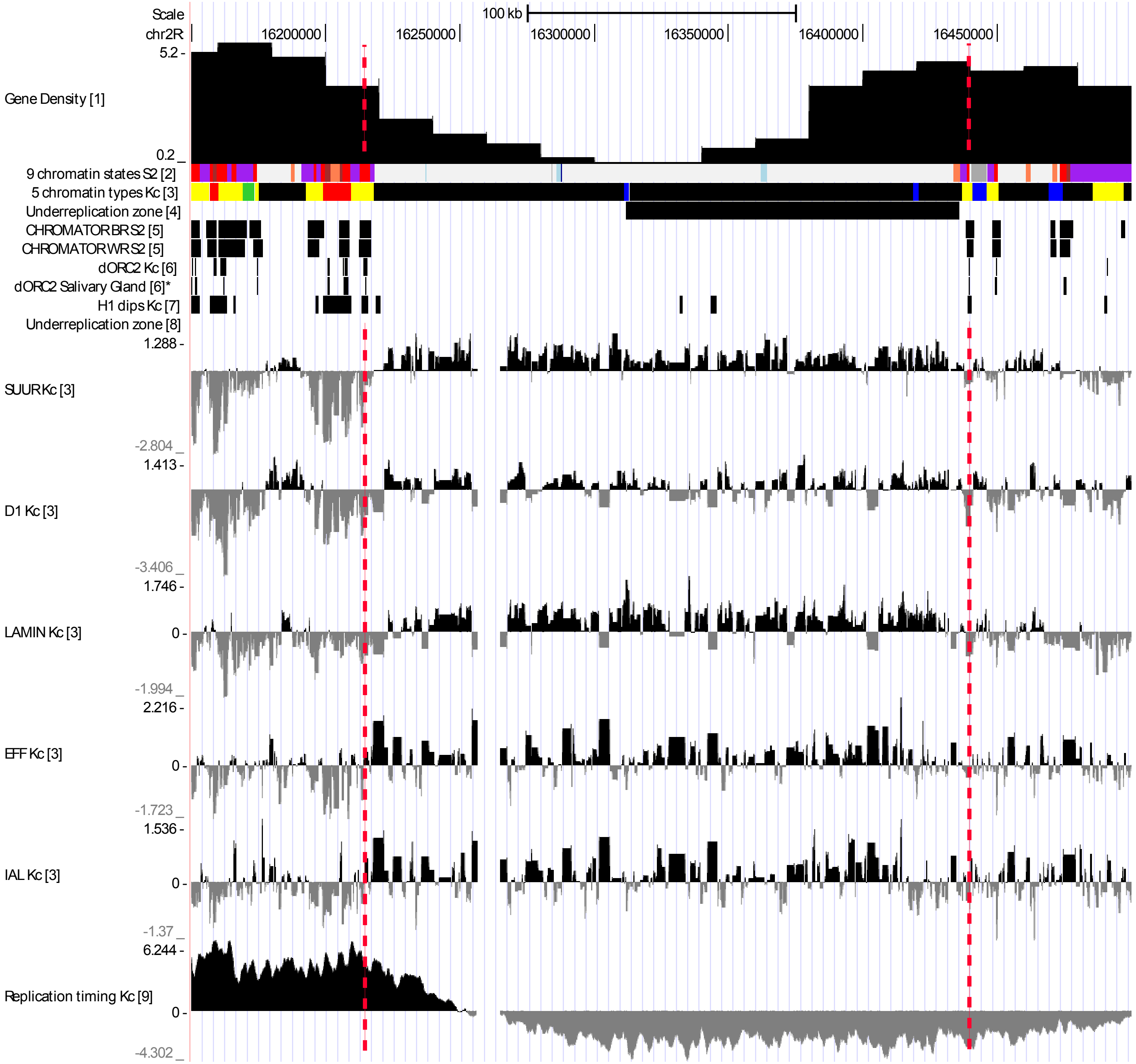

Scale
100 kb
chr2R
16200000
16250000
16300000
16350000
16400000
16450000
5.2 -
Gene Density [1]
0.2 _
9 chromatin states S2 [2]
5 chromatin types Kc [3]
Underreplication zone [4]
CHROMATOR BR S2 [5]
CHROMATOR WR S2 [5]
dORC2 Kc [6]
dORC2 Salivary Gland [6]*
H1 dips Kc [7]
Underreplication zone [8]
1.288 -
SUUR Kc [3]
-2.804 _
1.413 -
D1 Kc [3]
-3.406 _
1.746 -
LAMIN Kc [3]
0 -
-1.994 _
2.216 -
EFF Kc [3]
0 -
-1.723 _
1.536 -
IAL Kc [3]
0 -
-1.37 _
6.244 -
Replication timing Kc [9]
0 -
-4.302 _

58A3-4 and 58B1-2

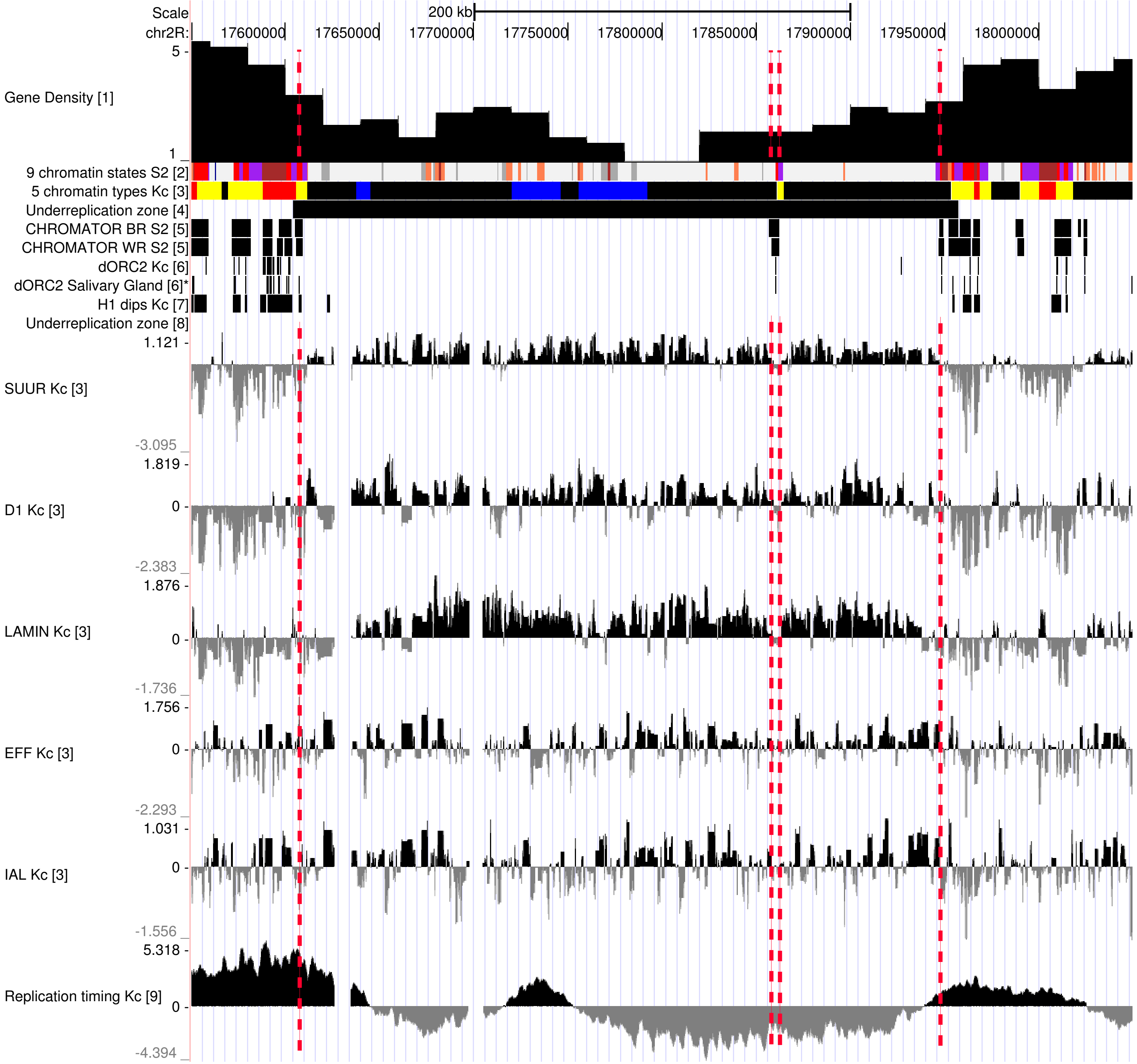

59D1-4

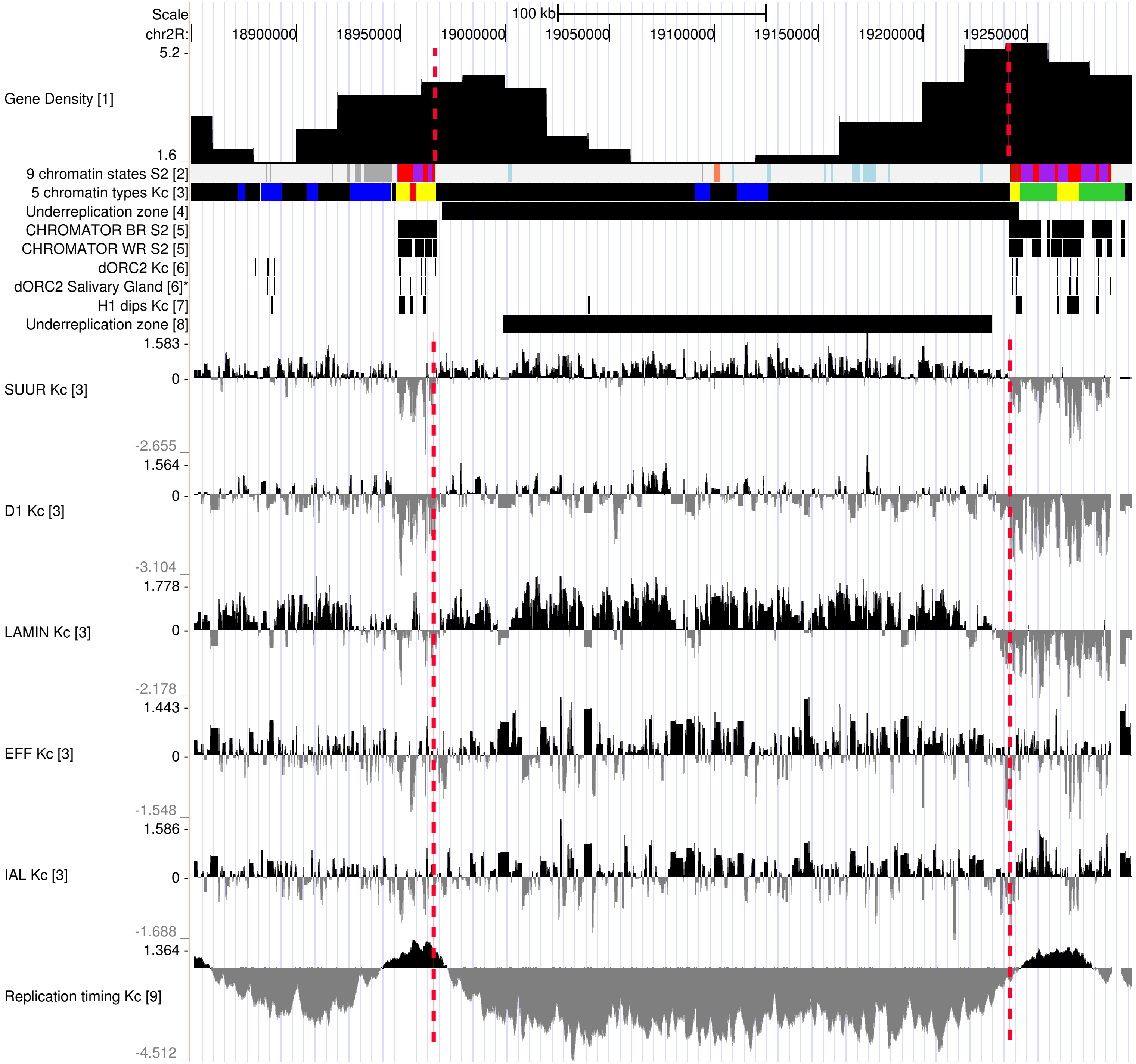

Scale
100 kb
chr2R:
18900000
18950000
19000000
19050000
19100000
19150000
19200000
19250000
5.2 -
Gene Density [1]
1.6 _
9 chromatin states S2 [2]
5 chromatin types Kc [3]
Underreplication zone [4]
CHROMATOR BR S2 [5]
CHROMATOR WR S2 [5]
dORC2 Kc [6]
dORC2 Salivary Gland [6]*
H1 dips Kc [7]
Underreplication zone [8]
1.583 -
0 -
SUUR Kc [3]
-2.655 _
1.564 -
0 -
D1 Kc [3]
-3.104 _
1.778 -
0 -
LAMIN Kc [3]
-2.178 _
1.443 -
0 -
EFF Kc [3]
-1.548 _
1.586 -
0 -
IAL Kc [3]
-1.688 _
1.364 -
Replication timing Kc [9]
-4.512 _

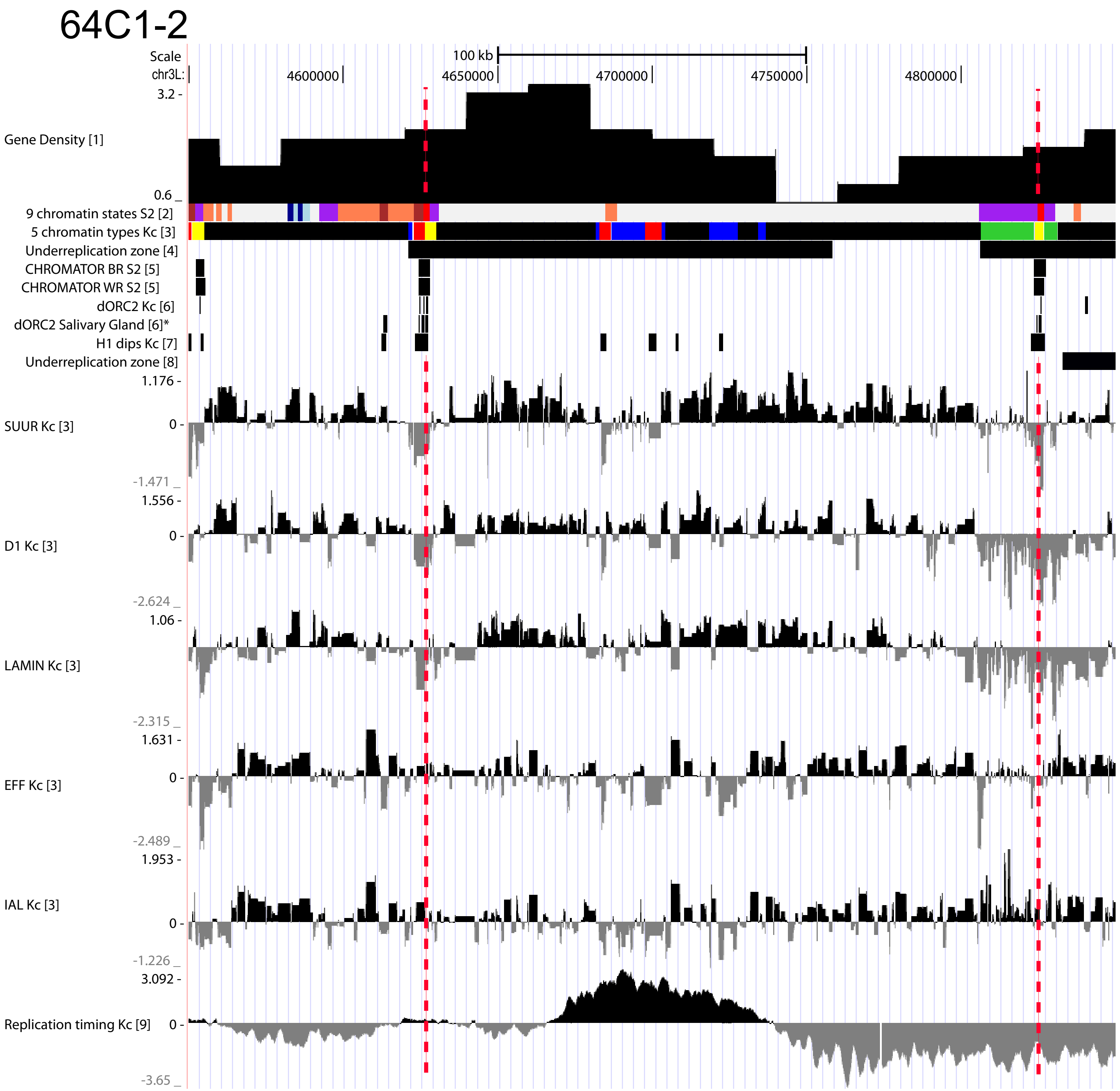
64C1-2
Scale
100 kb
chr3L:
4600000
4650000
4700000
4750000
4800000
Gene Density [1]
3.2 -
0.6 _
9 chromatin states S2 [2]
5 chromatin types Kc [3]
Underreplication zone [4]
CHROMATOR BR S2 [5]
CHROMATOR WR S2 [5]
dORC2 Kc [6]
dORC2 Salivary Gland [6]*
H1 dips Kc [7]
Underreplication zone [8]
SUUR Kc [3]
1.176 -
0 -
-1.471 _
D1 Kc [3]
1.556 -
0 -
-2.624 _
LAMIN Kc [3]
1.06 -
-2.315 _
EFF Kc [3]
1.631 -
0 -
-2.489 _
IAL Kc [3]
1.953 -
0 -
-1.226 _
Replication timing Kc [9]
3.092 -
0 -
-3.65 _

64C3-4

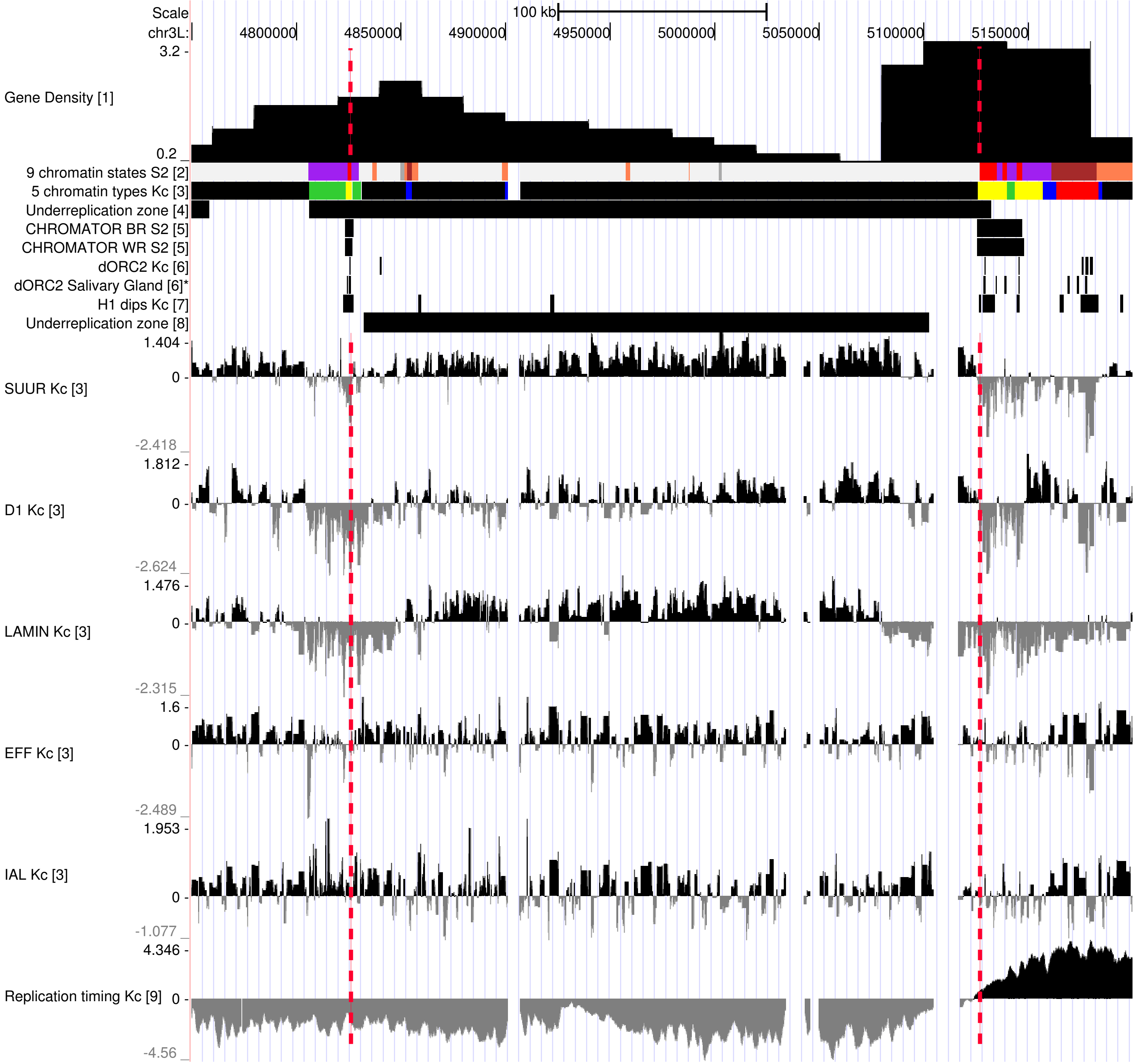
Scale
100 kb
chr3L:
4800000
4850000
4900000
4950000
5000000
5050000
5100000
5150000
3.2
Gene Density [1]
0.2
9 chromatin states S2 [2]
5 chromatin types Kc [3]
Underreplication zone [4]
CHROMATOR BR S2 [5]
CHROMATOR WR S2 [5]
dORC2 Kc [6]
dORC2 Salivary Gland [6]*
H1 dips Kc [7]
Underreplication zone [8]
1.404
0
SUUR Kc [3]
-2.418
1.812
0
D1 Kc [3]
-2.624
1.476
0
LAMIN Kc [3]
-2.315
1.6
0
EFF Kc [3]
-2.489
1.953
IAL Kc [3]
0
-1.077
4.346
Replication timing Kc [9]
0
-4.56

64D1-2

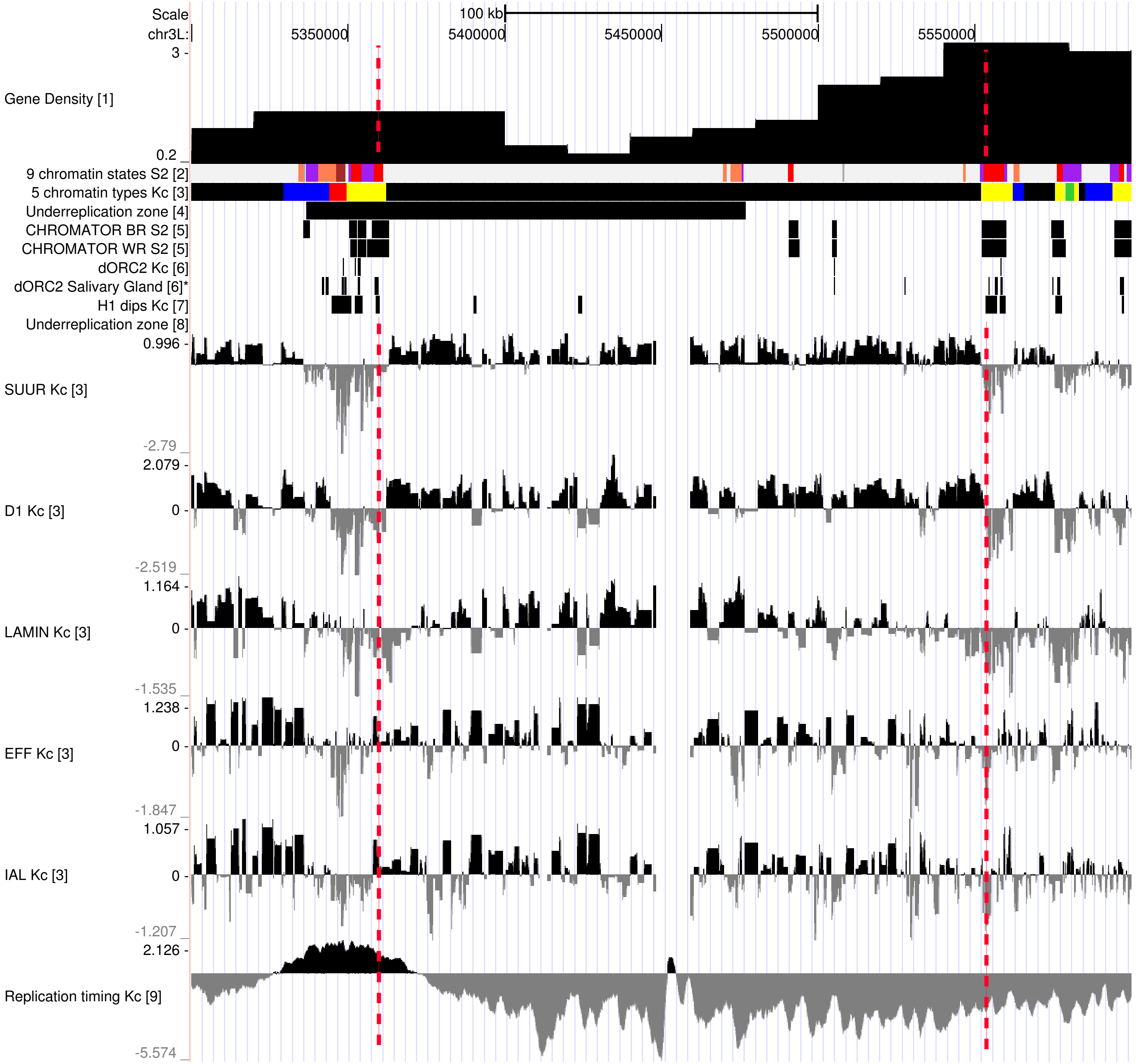

Scale
100 kb
chr3L:
5350000
5400000
5450000
5500000
5550000
3 -
Gene Density [1]
0.2 _
9 chromatin states S2 [2]
5 chromatin types Kc [3]
Underreplication zone [4]
CHROMATOR BR S2 [5]
CHROMATOR WR S2 [5]
dORC2 Kc [6]
dORC2 Salivary Gland [6]*
H1 dips Kc [7]
Underreplication zone [8]
0.996 -
SUUR Kc [3]
-2.79 _
2.079 -
D1 Kc [3]
0 -
-2.519 _
1.164 -
LAMIN Kc [3]
0 -
-1.535 _
1.238 -
EFF Kc [3]
0 -
-1.847 _
1.057 -
IAL Kc [3]
0 -
-1.207 _
2.126 -
Replication timing Kc [9]
-5.574 _

67D9-12

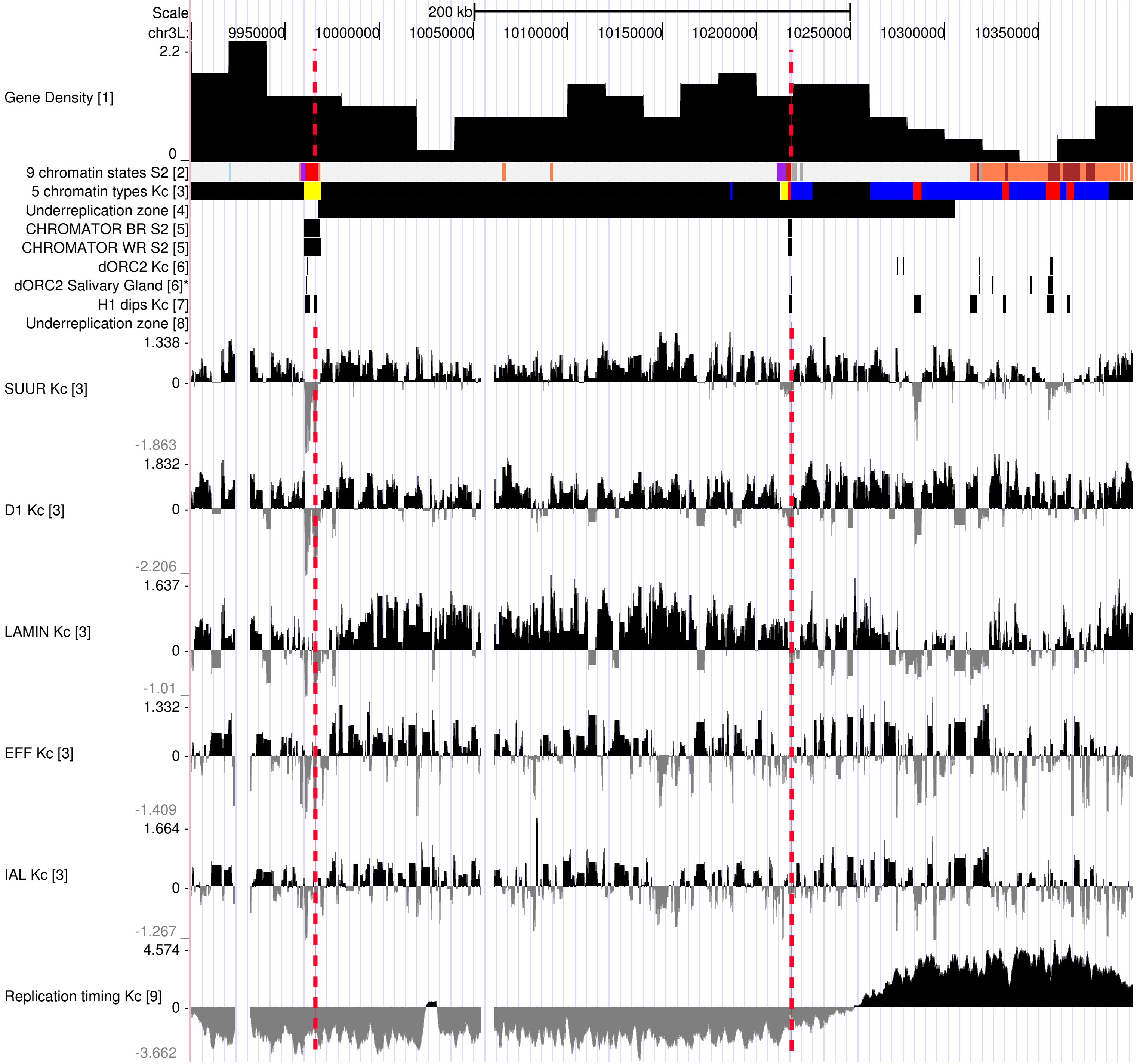
Scale
200 kb
chr3L:
9950000
10000000
10050000
10100000
10150000
10200000
10250000
10300000
10350000
2.2
Gene Density [1]
0
9 chromatin states S2 [2]
5 chromatin types Kc [3]
Underreplication zone [4]
CHROMATOR BR S2 [5]
CHROMATOR WR S2 [5]
dORC2 Kc [6]
dORC2 Salivary Gland [6]*
H1 dips Kc [7]
Underreplication zone [8]
1.338
SUUR Kc [3]
0
-1.863
1.832
D1 Kc [3]
0
-2.206
1.637
LAMIN Kc [3]
0
-1.01
1.332
EFF Kc [3]
0
-1.409
1.664
IAL Kc [3]
0
-1.267
4.574
Replication timing Kc [9]
0
-3.662

70A1-2 and 70A4-5

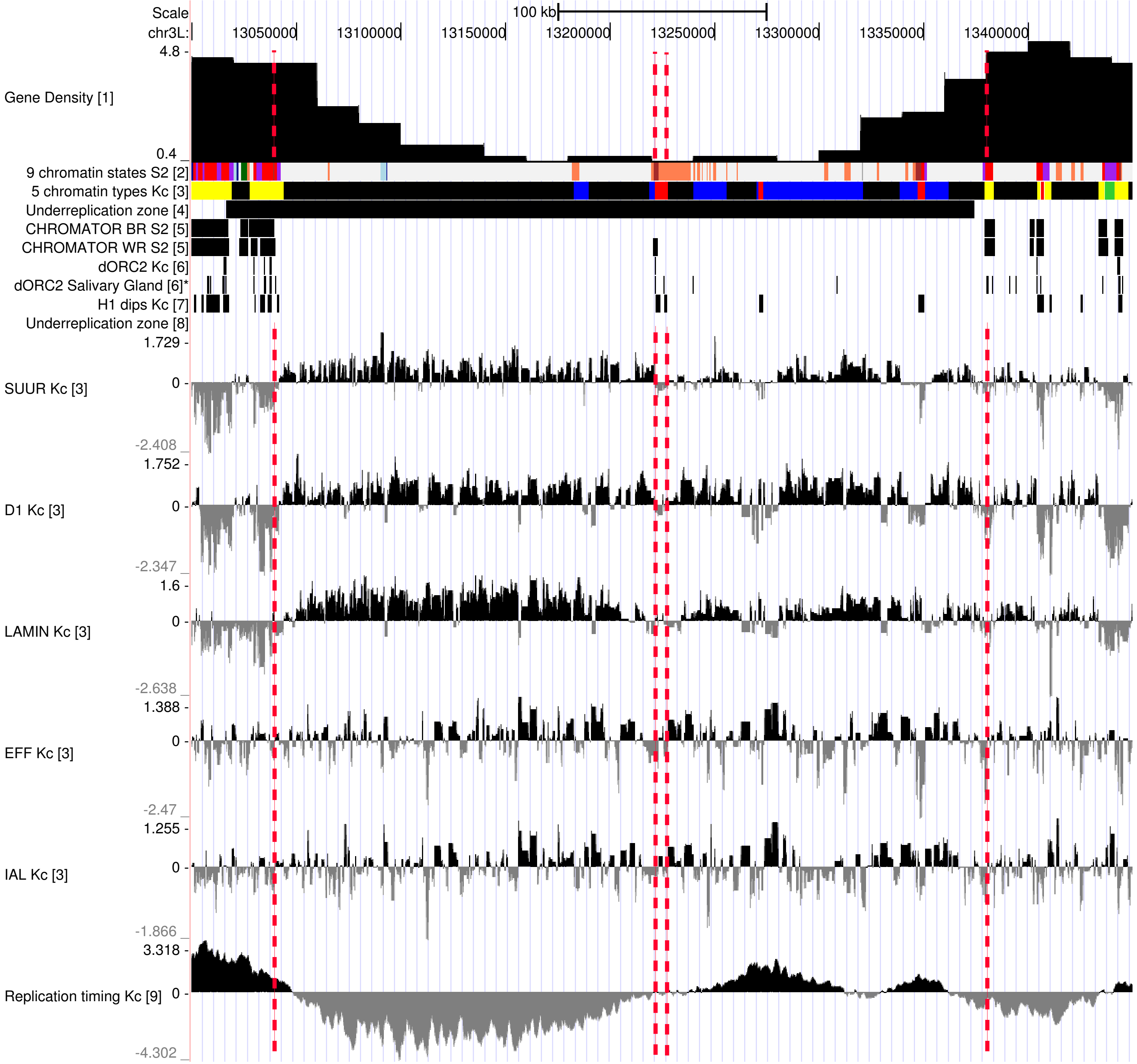

70C1-2

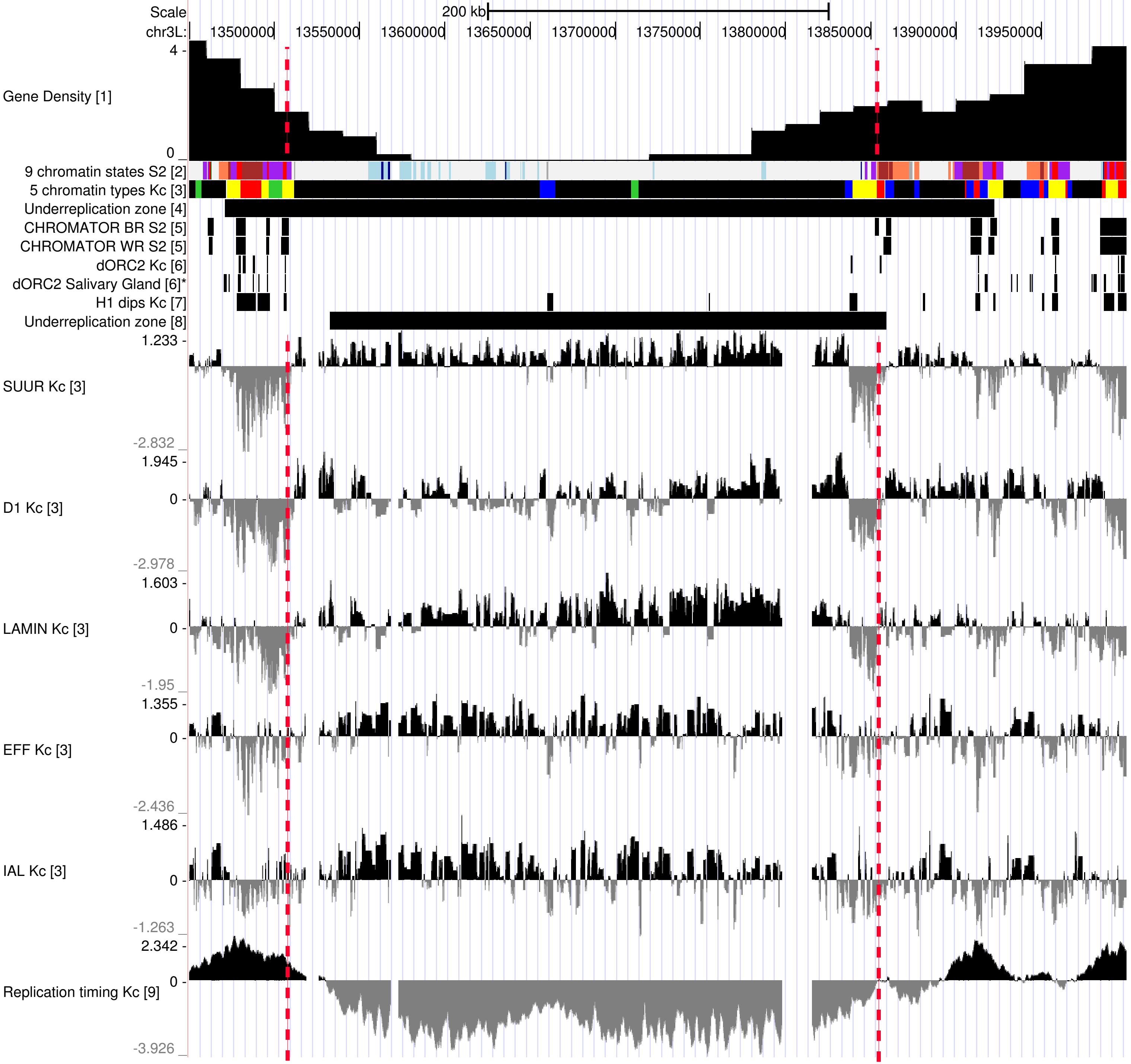
Scale
200 kb
chr3L:
13500000
13550000
13600000
13650000
13700000
13750000
13800000
13850000
13900000
13950000
4 -
Gene Density [1]
0 _
9 chromatin states S2 [2]
5 chromatin types Kc [3]
Underreplication zone [4]
CHROMATOR BR S2 [5]
CHROMATOR WR S2 [5]
dORC2 Kc [6]
dORC2 Salivary Gland [6]*
H1 dips Kc [7]
Underreplication zone [8]
1.233 -
SUUR Kc [3]
-2.832 _
1.945 -
0 -
D1 Kc [3]
-2.978 _
1.603 -
0 -
LAMIN Kc [3]
-1.95 _
1.355 -
0 -
EFF Kc [3]
-2.436 _
1.486 -
0 -
IAL Kc [3]
-1.263 _
2.342 -
0 -
Replication timing Kc [9]
-3.926 _

71C1-2

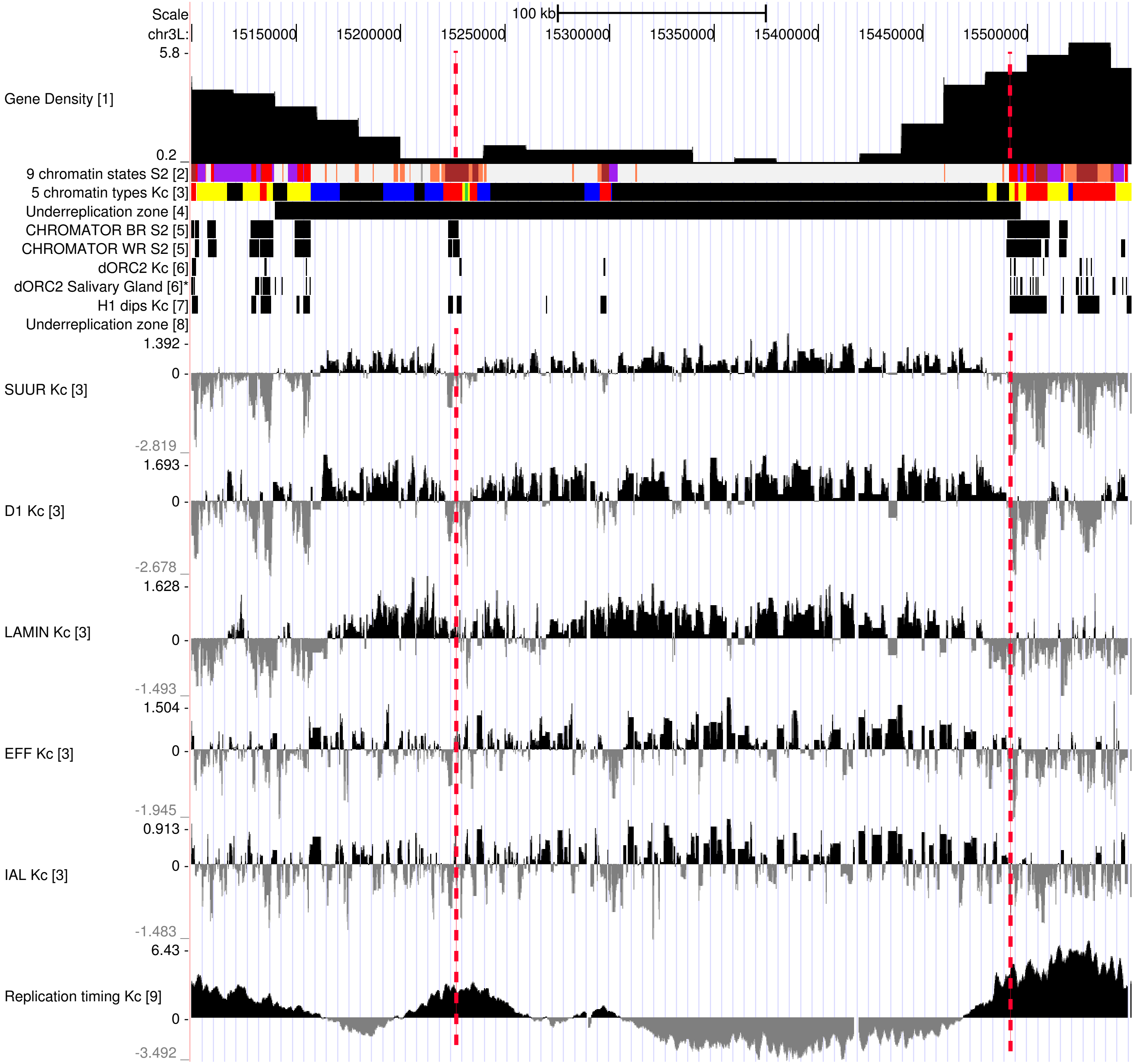

Scale
100 kb
chr3L:
15150000
15200000
15250000
15300000
15350000
15400000
15450000
15500000
5.8
Gene Density [1]
0.2
9 chromatin states S2 [2]
5 chromatin types Kc [3]
Underreplication zone [4]
CHROMATOR BR S2 [5]
CHROMATOR WR S2 [5]
dORC2 Kc [6]
dORC2 Salivary Gland [6]*
H1 dips Kc [7]
Underreplication zone [8]
1.392
0
SUUR Kc [3]
-2.819
1.693
0
D1 Kc [3]
-2.678
1.628
0
LAMIN Kc [3]
-1.493
1.504
0
EFF Kc [3]
-1.945
0.913
0
IAL Kc [3]
-1.483
6.43
Replication timing Kc [9]
0
-3.492

75C1-2

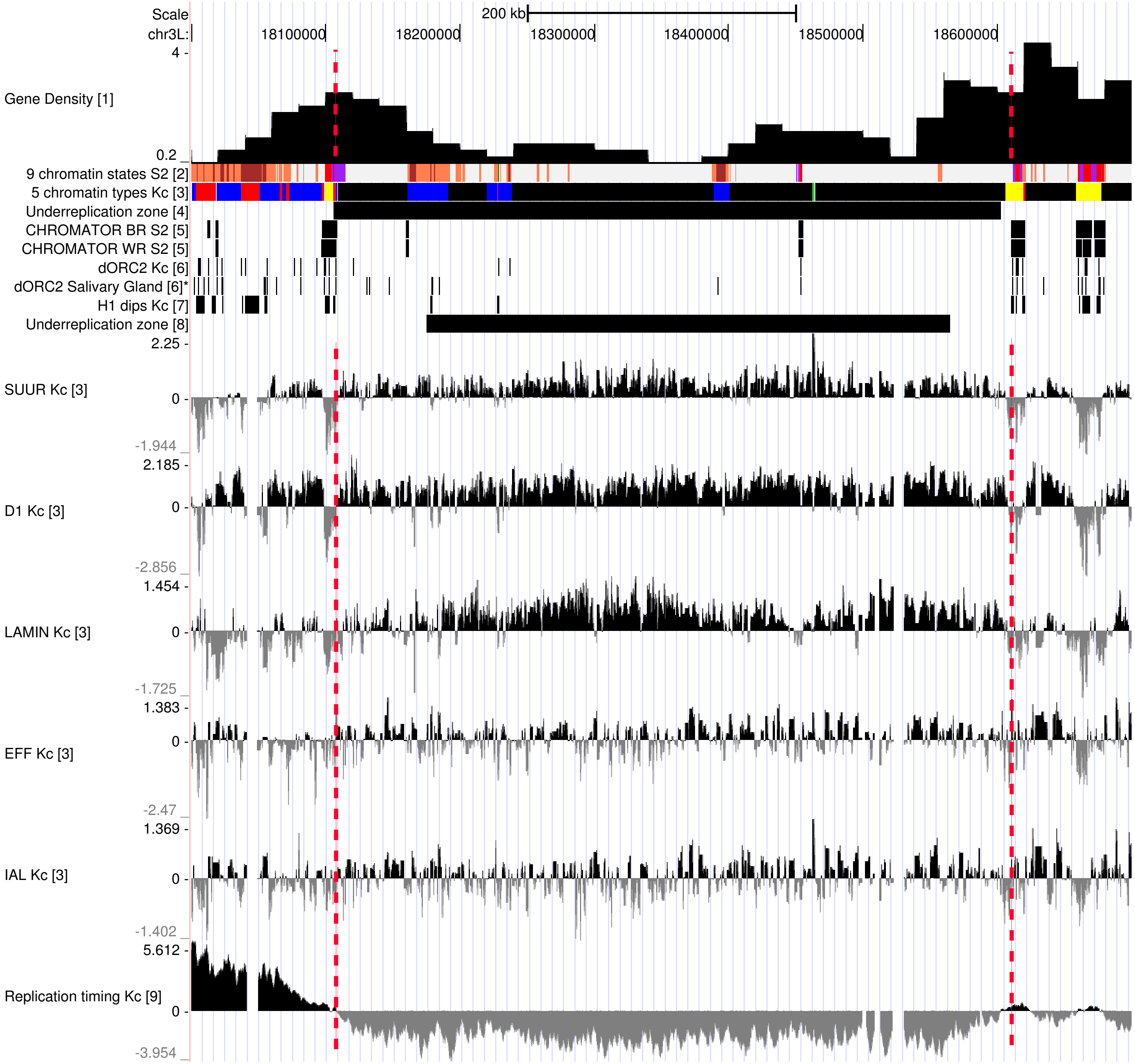

Scale
200 kb
chr3L:
18100000
18200000
18300000
18400000
18500000
18600000
4 -
Gene Density [1]
0.2 _
9 chromatin states S2 [2]
5 chromatin types Kc [3]
Underreplication zone [4]
CHROMATOR BR S2 [5]
CHROMATOR WR S2 [5]
dORC2 Kc [6]
dORC2 Salivary Gland [6]*
H1 dips Kc [7]
Underreplication zone [8]
2.25 -
SUUR Kc [3]
0 -
-1.944 _
2.185 -
D1 Kc [3]
0 -
-2.856 _
1.454 -
LAMIN Kc [3]
0 -
-1.725 _
1.383 -
EFF Kc [3]
0 -
-2.47 _
1.369 -
IAL Kc [3]
0 -
-1.402 _
5.612 -
Replication timing Kc [9]
0 -
-3.954 _

77E1-4

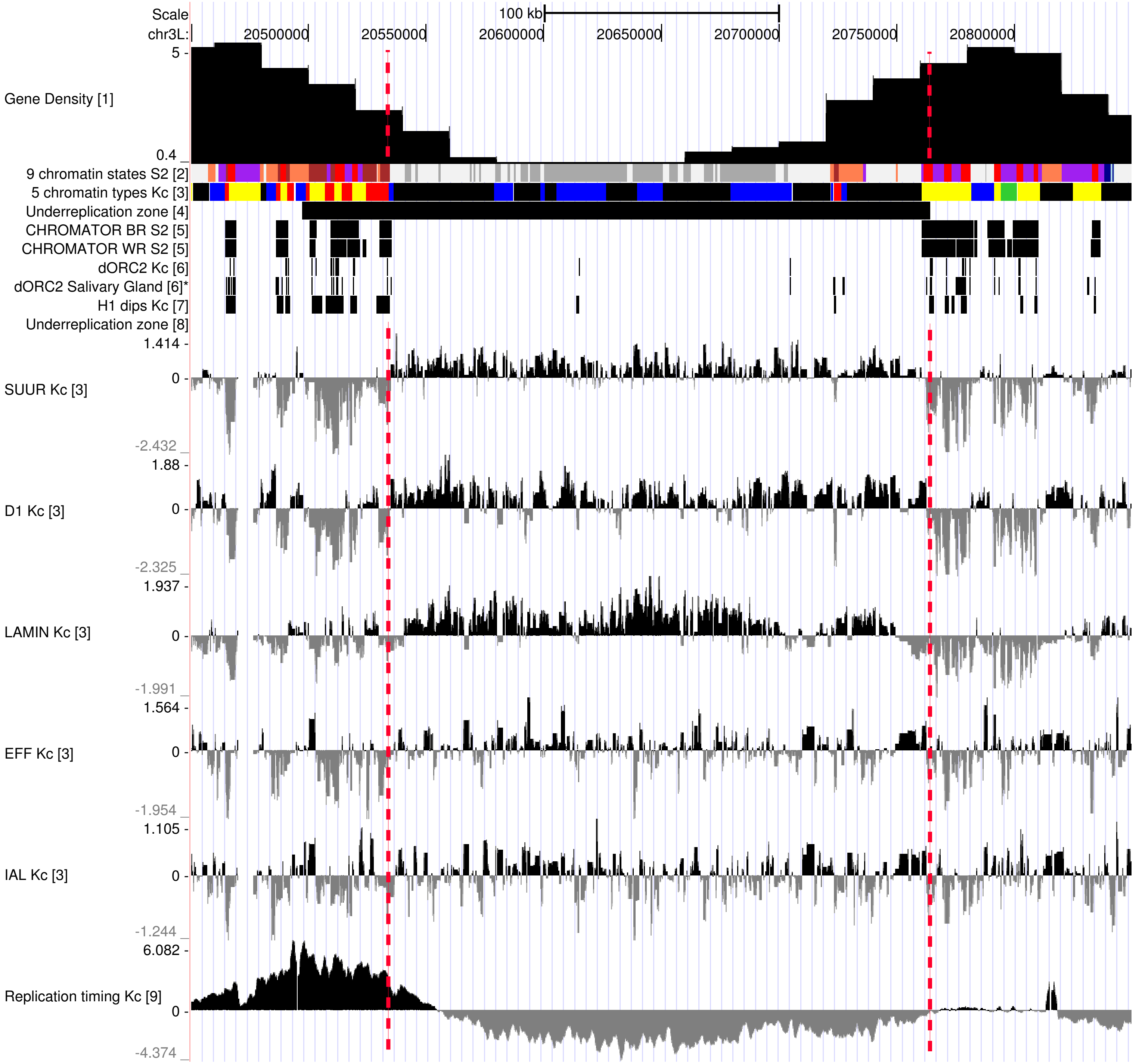

Scale
100 kb
chr3L:
20500000
20550000
20600000
20650000
20700000
20750000
20800000
5 -
Gene Density [1]
0.4 _
9 chromatin states S2 [2]
5 chromatin types Kc [3]
Underreplication zone [4]
CHROMATOR BR S2 [5]
CHROMATOR WR S2 [5]
dORC2 Kc [6]
dORC2 Salivary Gland [6]*
H1 dips Kc [7]
Underreplication zone [8]
1.414 -
SUUR Kc [3]
0 -
-2.432 _
1.88 -
D1 Kc [3]
0 -
-2.325 _
1.937 -
LAMIN Kc [3]
0 -
-1.991 _
1.564 -
EFF Kc [3]
0 -
-1.954 _
1.105 -
IAL Kc [3]
0 -
-1.244 _
6.082 -
Replication timing Kc [9]
0 -
-4.374 _

79E1-4

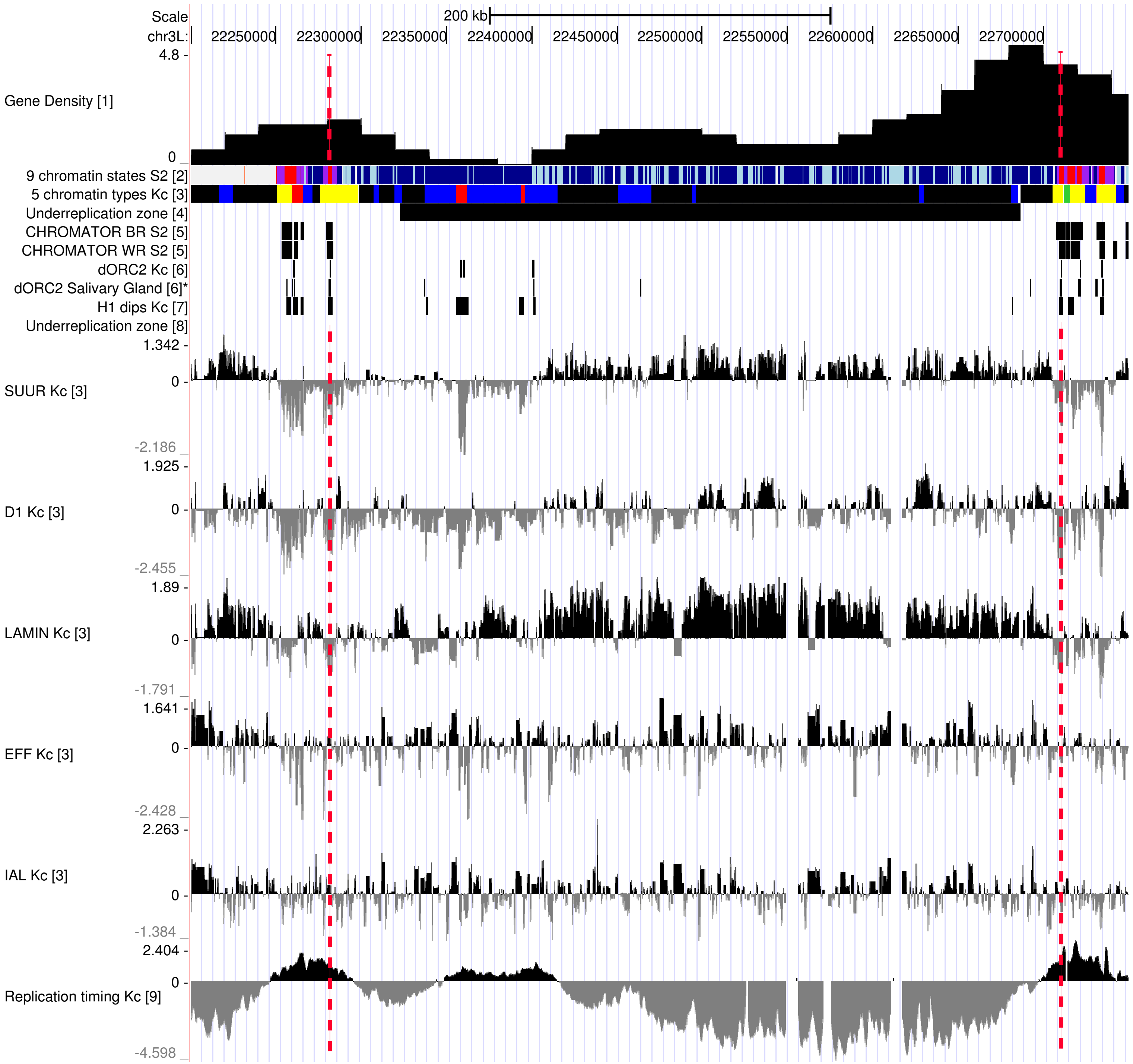

Scale
200 kb
chr3L:
22250000
22300000
22350000
22400000
22450000
22500000
22550000
22600000
22650000
22700000
4.8 -
Gene Density [1]
0 _
9 chromatin states S2 [2]
5 chromatin types Kc [3]
Underreplication zone [4]
CHROMATOR BR S2 [5]
CHROMATOR WR S2 [5]
dORC2 Kc [6]
dORC2 Salivary Gland [6]*
H1 dips Kc [7]
Underreplication zone [8]
1.342 -
0 -
SUUR Kc [3]
-2.186 _
1.925 -
0 -
D1 Kc [3]
-2.455 _
1.89 -
0 -
LAMIN Kc [3]
-1.791 _
1.641 -
0 -
EFF Kc [3]
-2.428 _
2.263 -
IAL Kc [3]
0 -
-1.384 _
2.404 -
0 -
Replication timing Kc [9]
-4.598 _

83E1-2

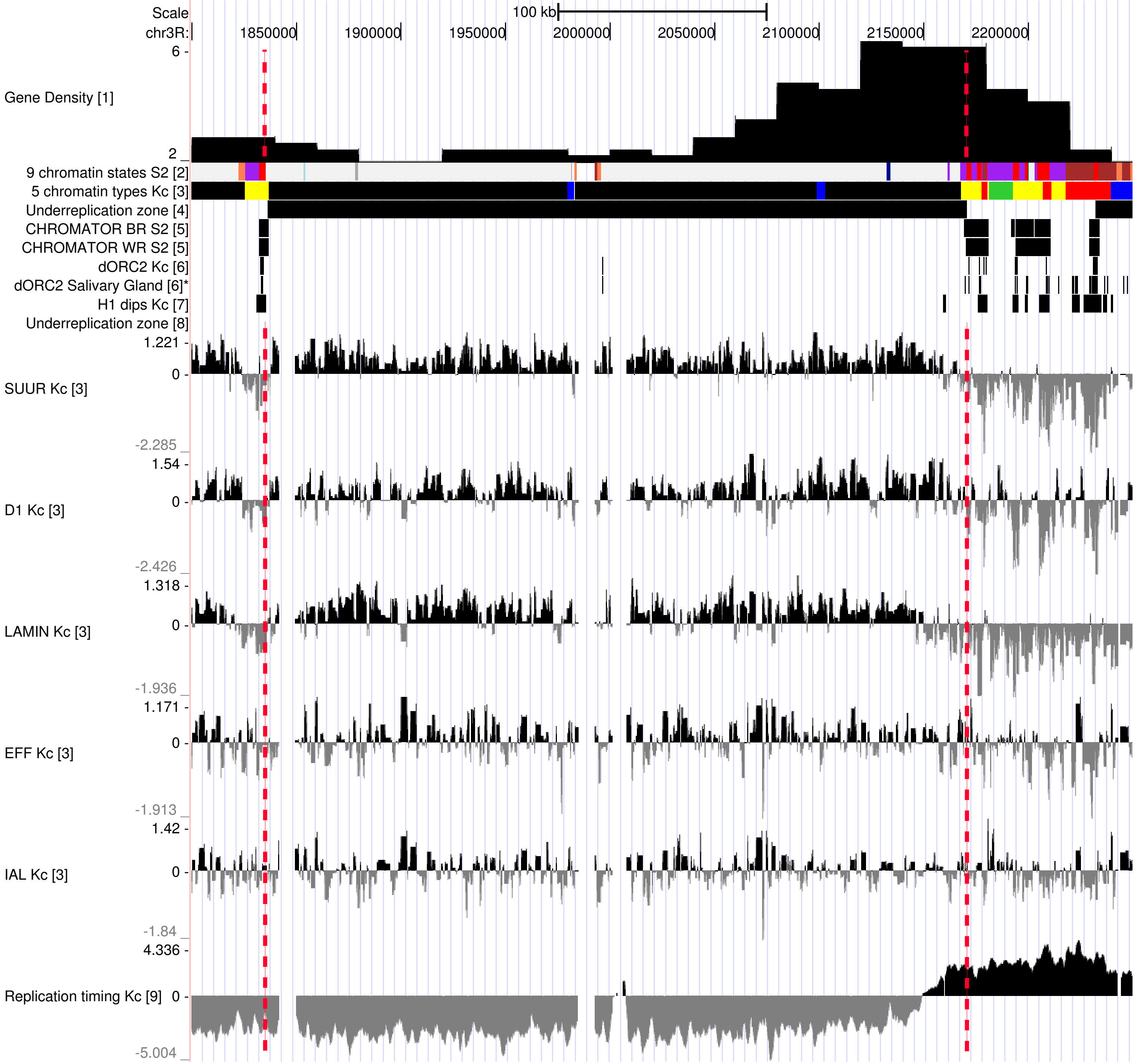

Scale
100 kb
chr3R:
1850000
1900000
1950000
2000000
2050000
2100000
2150000
2200000
Gene Density [1]
9 chromatin states S2 [2]
5 chromatin types Kc [3]
Underreplication zone [4]
CHROMATOR BR S2 [5]
CHROMATOR WR S2 [5]
dORC2 Kc [6]
dORC2 Salivary Gland [6]*
H1 dips Kc [7]
Underreplication zone [8]
SUUR Kc [3]
D1 Kc [3]
LAMIN Kc [3]
EFF Kc [3]
IAL Kc [3]
Replication timing Kc [9]
6
2
1.221
0
-2.285
1.54
-2.426
1.318
-1.936
1.171
-1.913
1.42
-1.84
4.336
-5.004

84A1-2

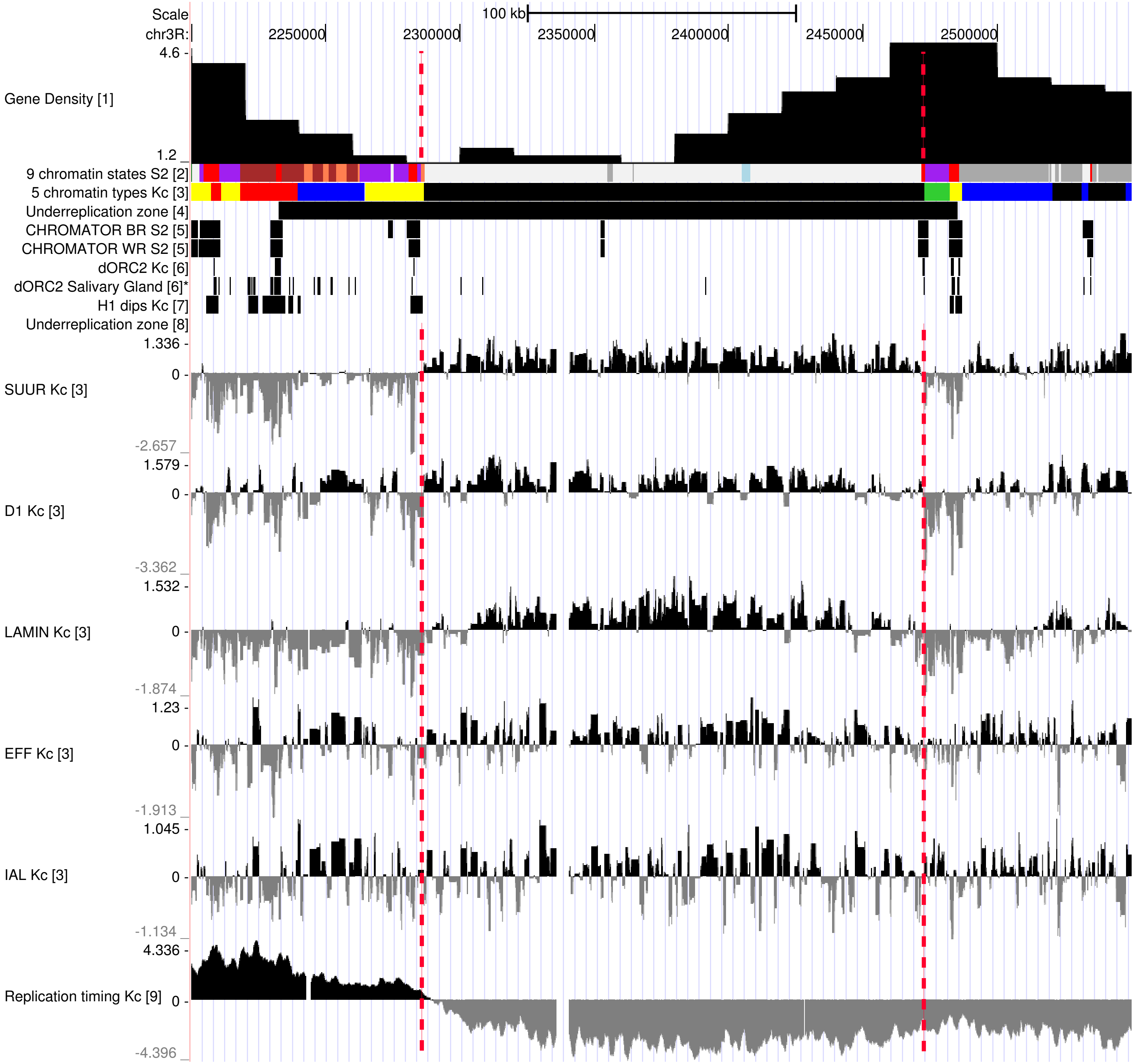
Scale
100 kb
chr3R:
2250000
2300000
2350000
2400000
2450000
2500000
4.6 -
Gene Density [1]
1.2 _
9 chromatin states S2 [2]
5 chromatin types Kc [3]
Underreplication zone [4]
CHROMATOR BR S2 [5]
CHROMATOR WR S2 [5]
dORC2 Kc [6]
dORC2 Salivary Gland [6]*
H1 dips Kc [7]
Underreplication zone [8]
1.336 -
0 -
SUUR Kc [3]
-2.657 _
1.579 -
0 -
D1 Kc [3]
-3.362 _
1.532 -
0 -
LAMIN Kc [3]
-1.874 _
1.23 -
0 -
EFF Kc [3]
-1.913 _
1.045 -
0 -
IAL Kc [3]
-1.134 _
4.336 -
Replication timing Kc [9]
0 -
-4.396 _

84D3-4 and 84D9-10

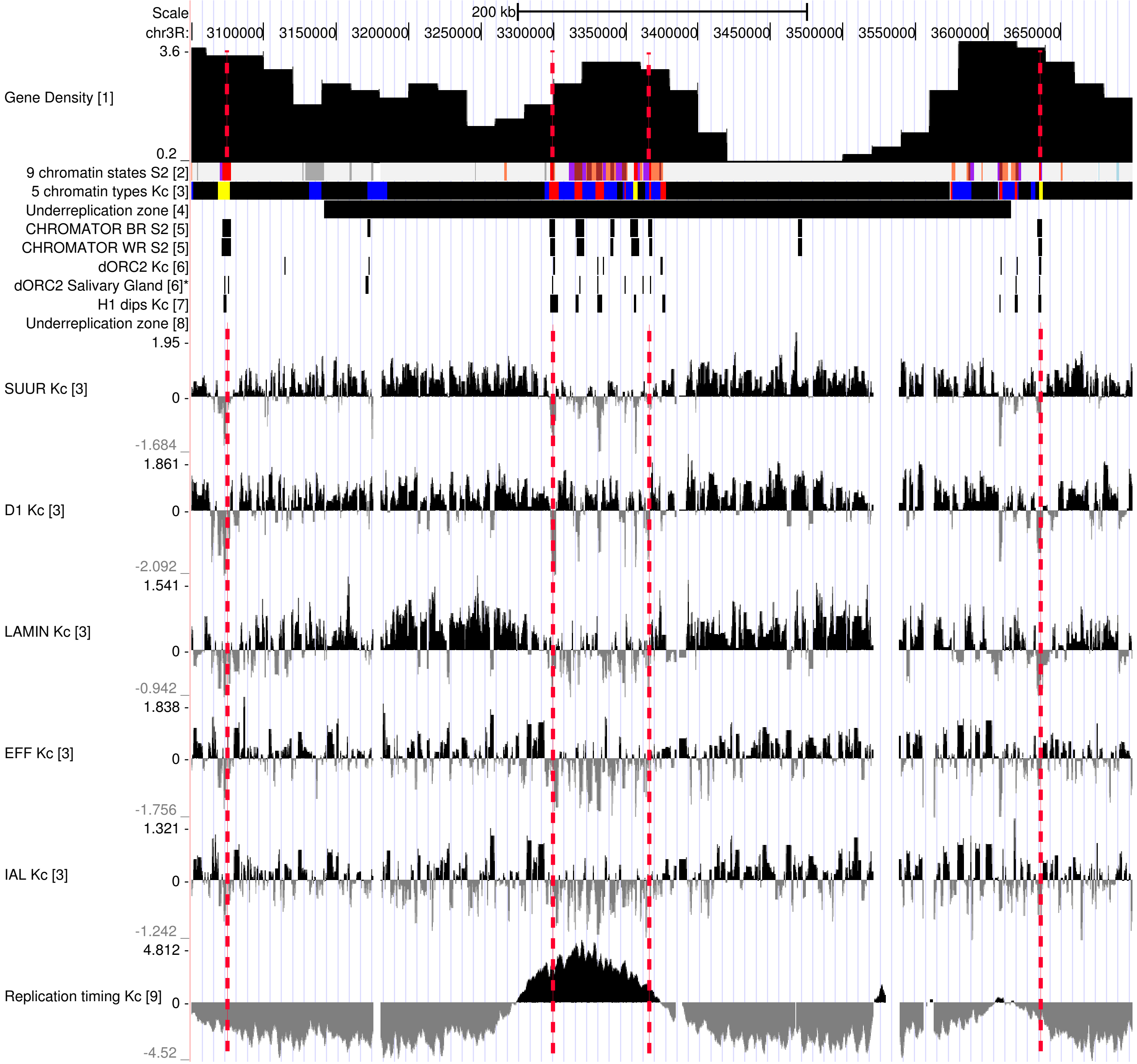

86D1-2

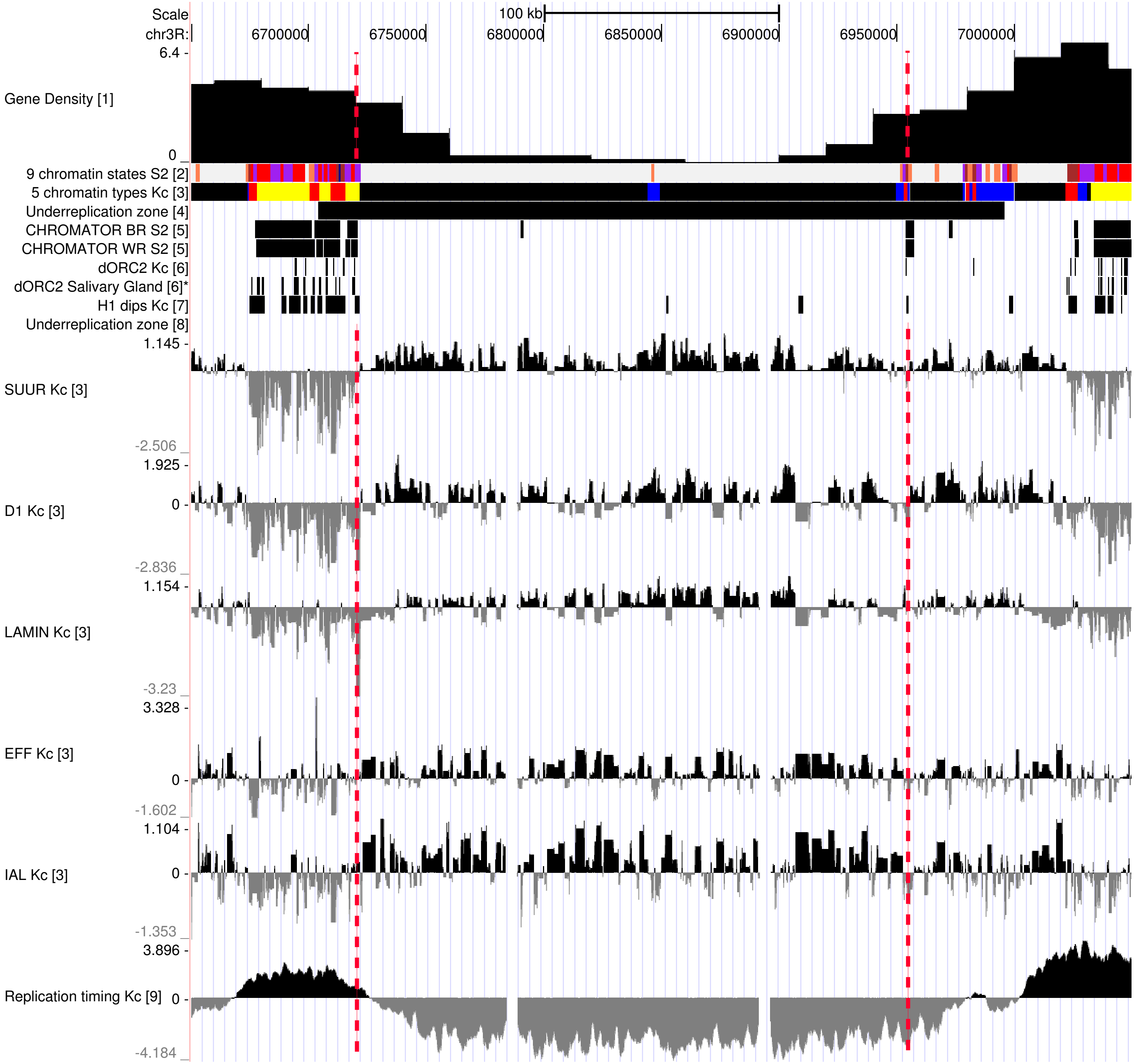

Scale
100 kb
chr3R:
6700000
6750000
6800000
6850000
6900000
6950000
7000000
6.4 -
Gene Density [1]
0 _
9 chromatin states S2 [2]
5 chromatin types Kc [3]
Underreplication zone [4]
CHROMATOR BR S2 [5]
CHROMATOR WR S2 [5]
dORC2 Kc [6]
dORC2 Salivary Gland [6]*
H1 dips Kc [7]
Underreplication zone [8]
1.145 -
SUUR Kc [3]
-2.506 _
1.925 -
D1 Kc [3]
0 -
-2.836 _
1.154 -
LAMIN Kc [3]
-3.23 _
3.328 -
EFF Kc [3]
0 -
-1.602 _
1.104 -
IAL Kc [3]
0 -
-1.353 _
3.896 -
Replication timing Kc [9]
0 -
-4.184 _

87B1-2 and 87B4-5

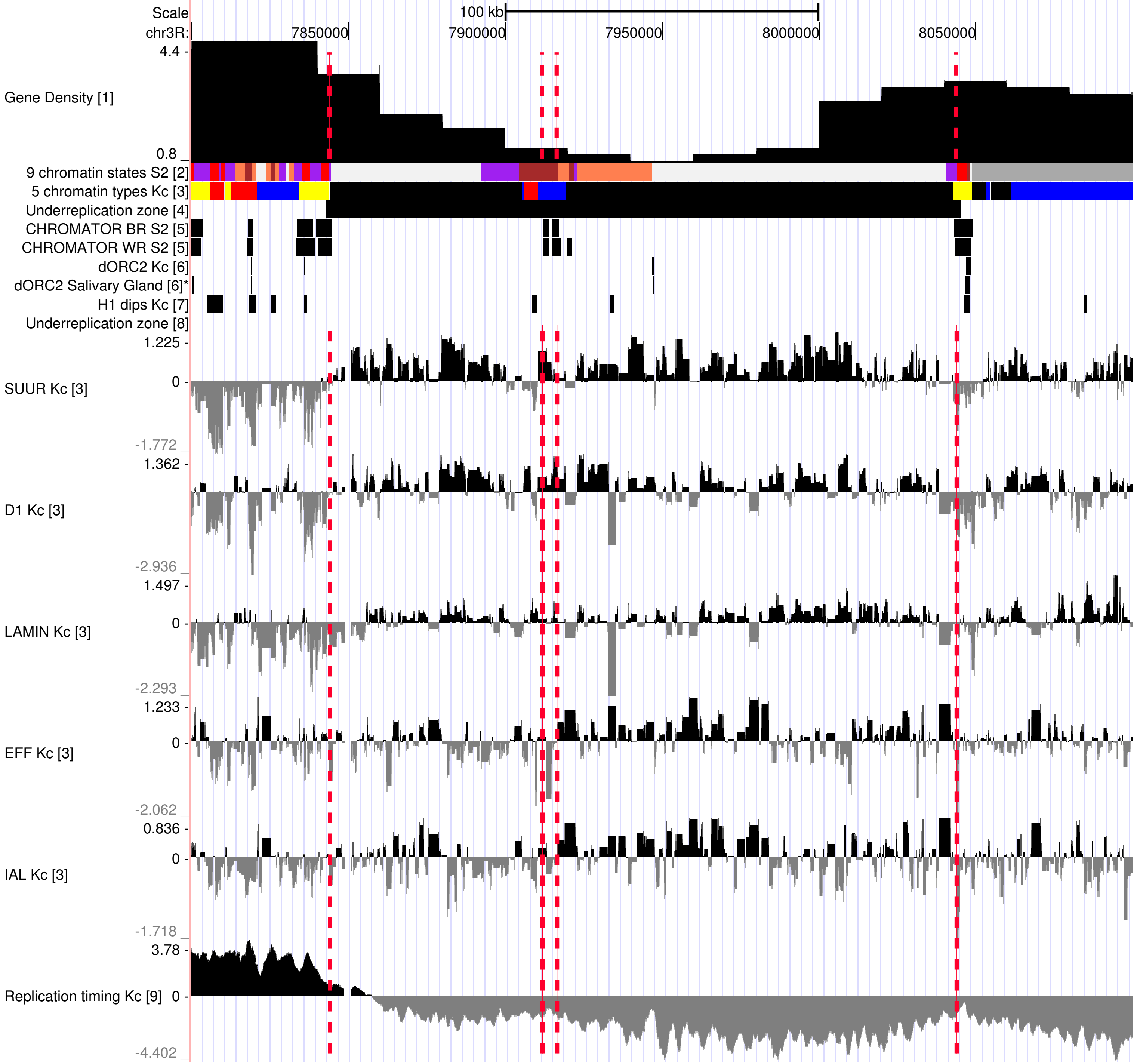

87D1-2

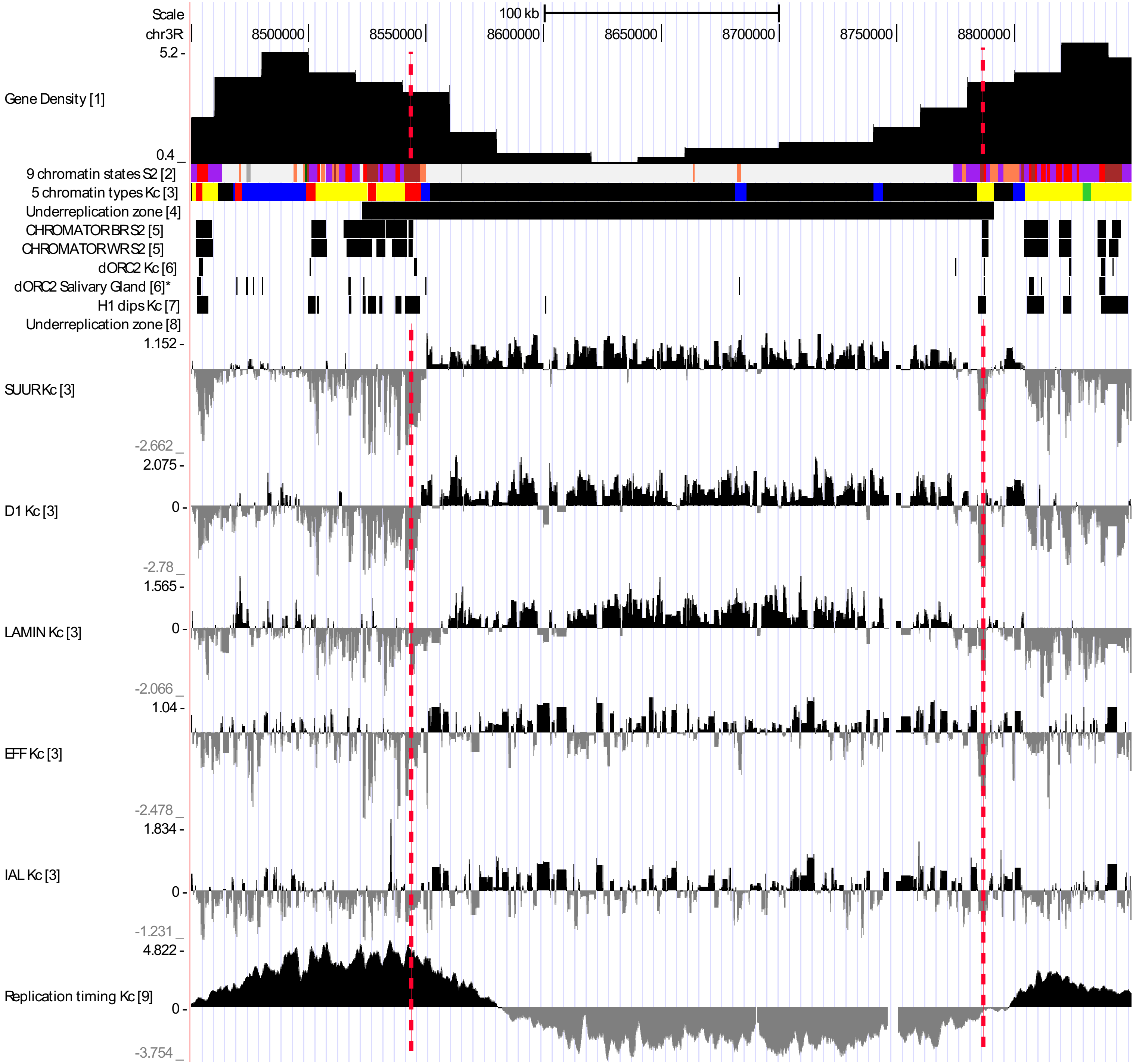

Scale
chr3R
100 kb
8500000
8550000
8600000
8650000
8700000
8750000
8800000
5.2 -
Gene Density [1]
0.4 _
9 chromatin states S2 [2]
5 chromatin types Kc [3]
Underreplication zone [4]
CHROMATOR BR S2 [5]
CHROMATOR WR S2 [5]
dORC2 Kc [6]
dORC2 Salivary Gland [6]*
H1 dips Kc [7]
Underreplication zone [8]
1.152 -
SUUR Kc [3]
-2.662 _
2.075 -
D1 Kc [3]
0 -
-2.78 _
1.565 -
LAMIN Kc [3]
0 -
-2.066 _
1.04 -
EFF Kc [3]
-2.478 _
1.834 -
IAL Kc [3]
0 -
-1.231 _
4.822 -
Replication timing Kc [9]
0 -
-3.754 _

89A1-2 and 89A8-9

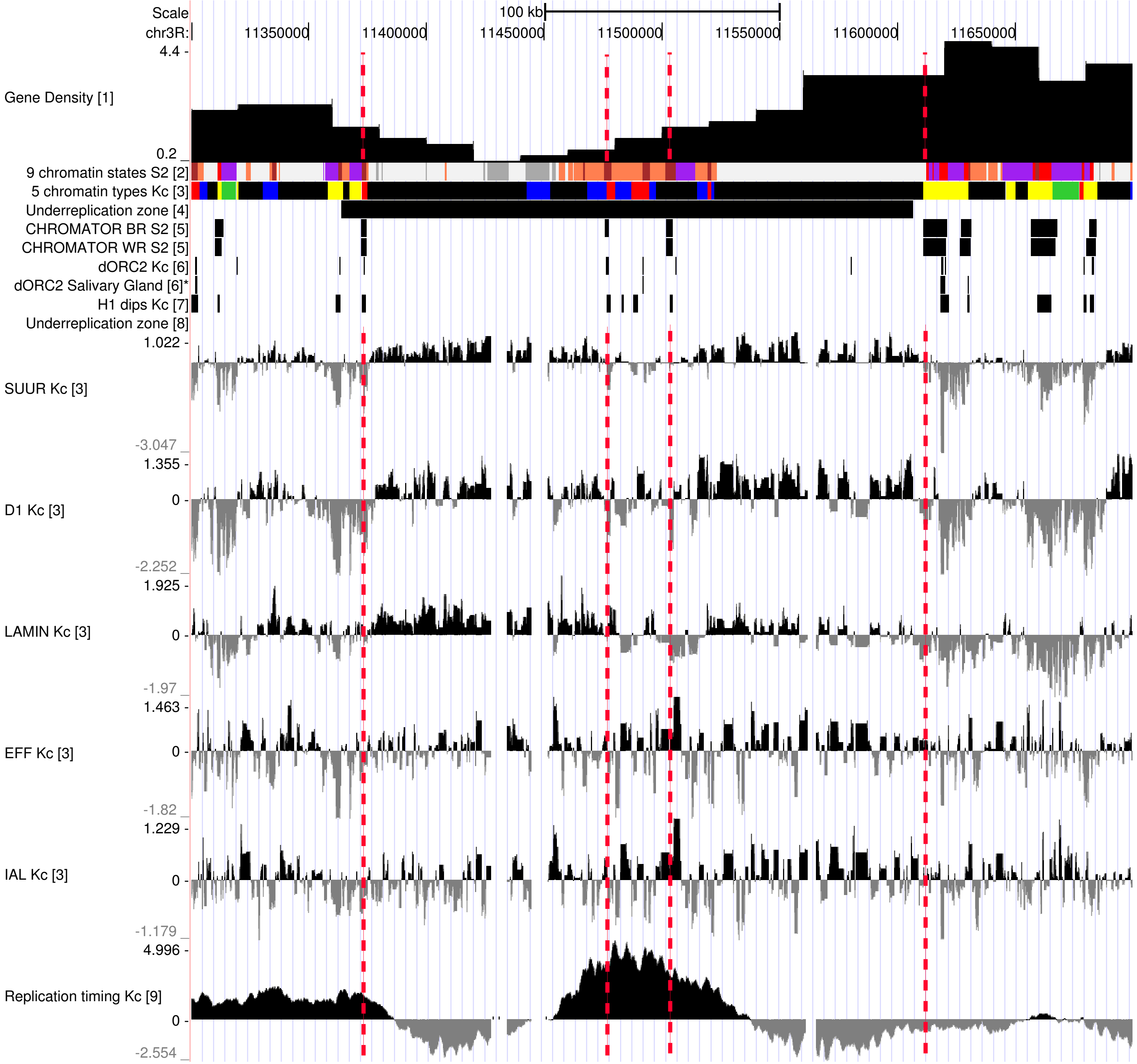

89E1-4

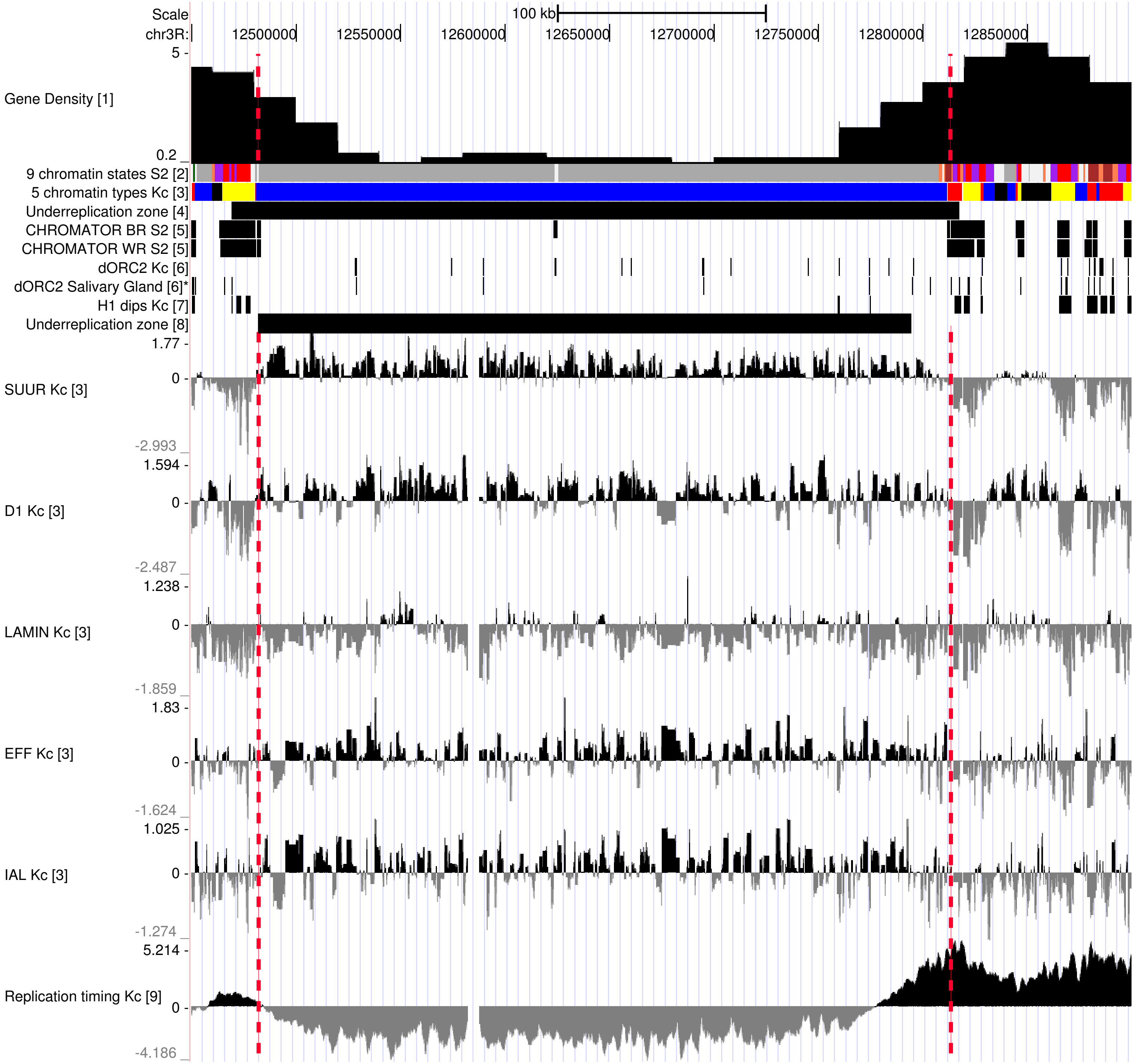
Scale
100 kb
chr3R:
12500000
12550000
12600000
12650000
12700000
12750000
12800000
12850000
5
0.2
Gene Density [1]
9 chromatin states S2 [2]
5 chromatin types Kc [3]
Underreplication zone [4]
CHROMATOR BR S2 [5]
CHROMATOR WR S2 [5]
dORC2 Kc [6]
dORC2 Salivary Gland [6]*
H1 dips Kc [7]
Underreplication zone [8]
1.77
0
-2.993
SUUR Kc [3]
1.594
0
-2.487
D1 Kc [3]
1.238
0
-1.859
LAMIN Kc [3]
1.83
0
-1.624
EFF Kc [3]
1.025
0
-1.274
IAL Kc [3]
5.214
0
-4.186
Replication timing Kc [9]

92D1-4 and 92E1-2

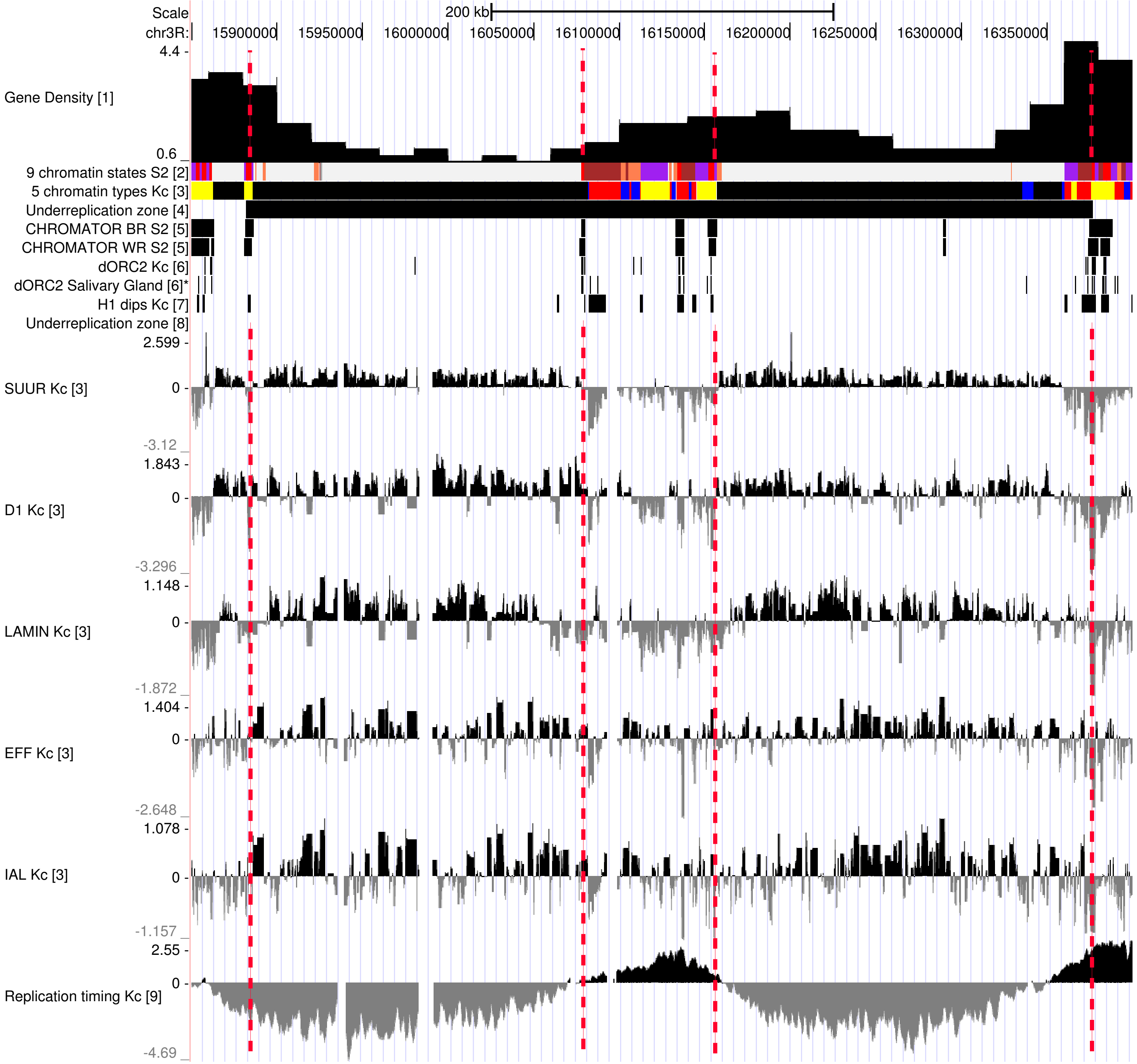

94A1-4

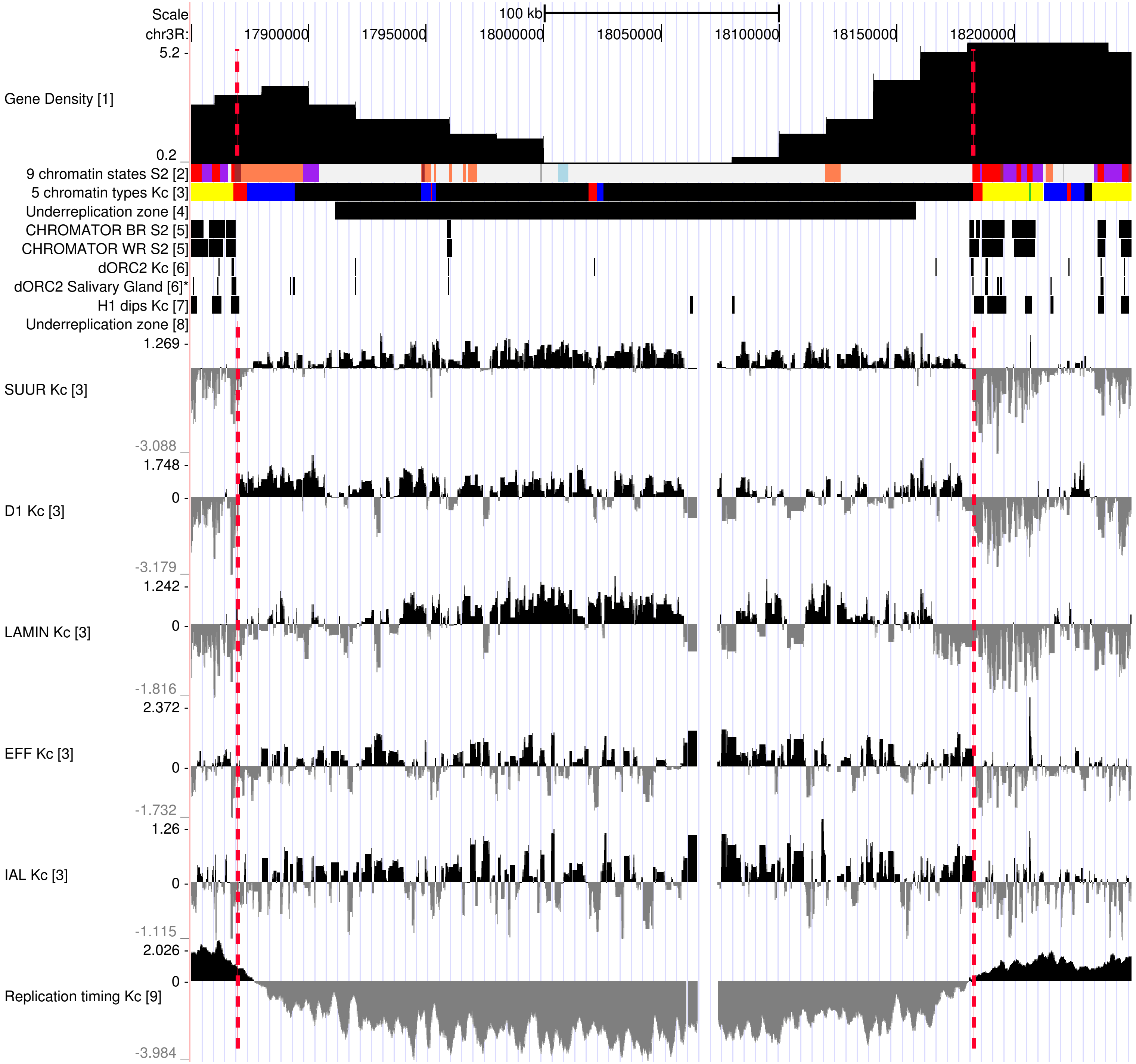

Scale
100 kb
chr3R:
17900000
17950000
18000000
18050000
18100000
18150000
18200000
5.2 -
Gene Density [1]
0.2 _
9 chromatin states S2 [2]
5 chromatin types Kc [3]
Underreplication zone [4]
CHROMATOR BR S2 [5]
CHROMATOR WR S2 [5]
dORC2 Kc [6]
dORC2 Salivary Gland [6]*
H1 dips Kc [7]
Underreplication zone [8]
1.269 -
SUUR Kc [3]
-3.088 _
1.748 -
0 -
D1 Kc [3]
-3.179 _
1.242 -
0 -
LAMIN Kc [3]
-1.816 _
2.372 -
EFF Kc [3]
0 -
-1.732 _
1.26 -
IAL Kc [3]
0 -
-1.115 _
2.026 -
0 -
Replication timing Kc [9]
-3.984 _

98C1-2

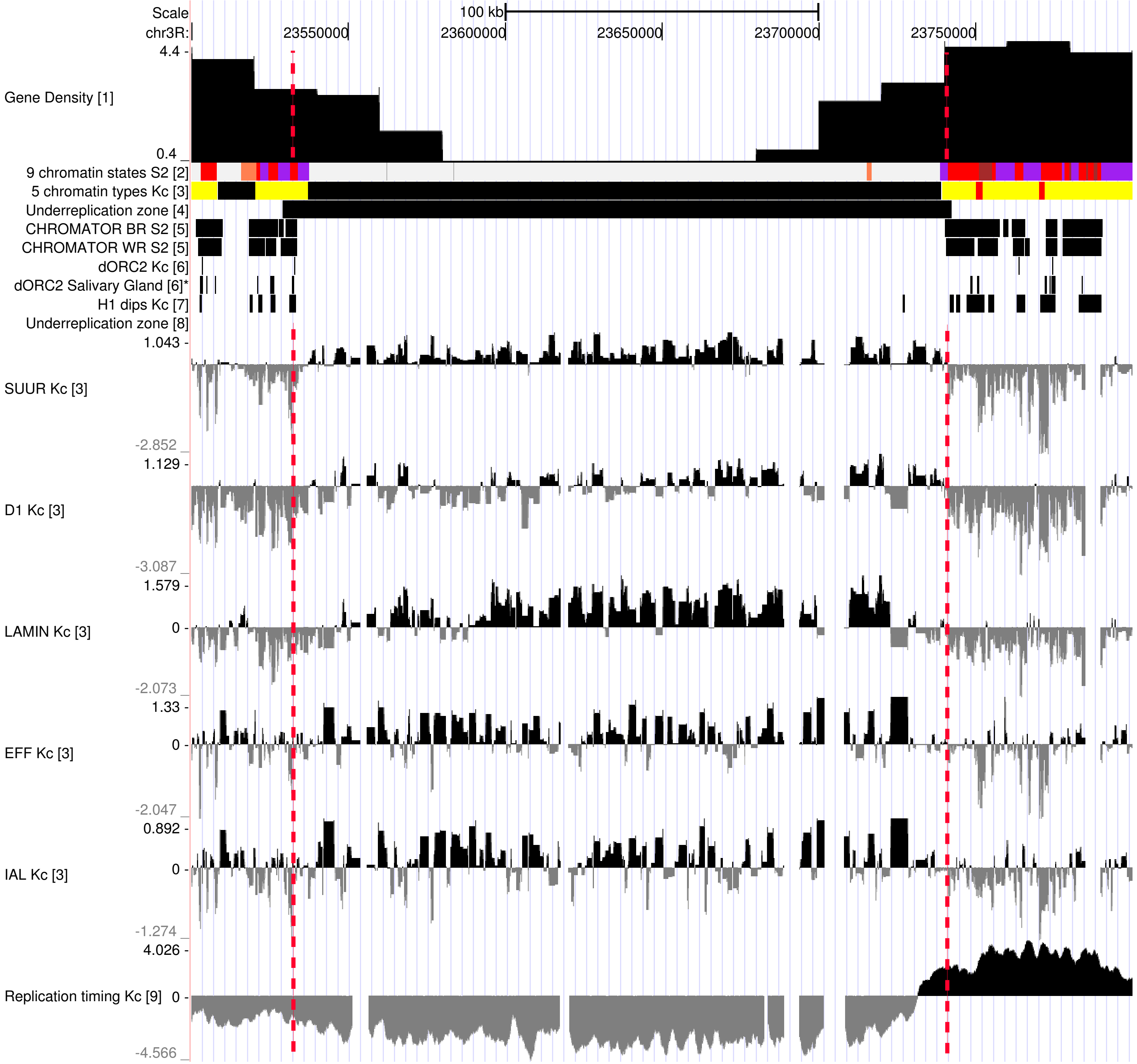
Scale
100 kb
chr3R:
23550000
23600000
23650000
23700000
23750000
4.4 -
Gene Density [1]
0.4 _
9 chromatin states S2 [2]
5 chromatin types Kc [3]
Underreplication zone [4]
CHROMATOR BR S2 [5]
CHROMATOR WR S2 [5]
dORC2 Kc [6]
dORC2 Salivary Gland [6]*
H1 dips Kc [7]
Underreplication zone [8]
1.043 -
SUUR Kc [3]
-2.852 _
1.129 -
D1 Kc [3]
-3.087 _
1.579 -
LAMIN Kc [3]
0
-2.073 _
1.33 -
EFF Kc [3]
0
-2.047 _
0.892 -
IAL Kc [3]
0
-1.274 _
4.026 -
Replication timing Kc [9]
0
-4.566 _

100A1-2

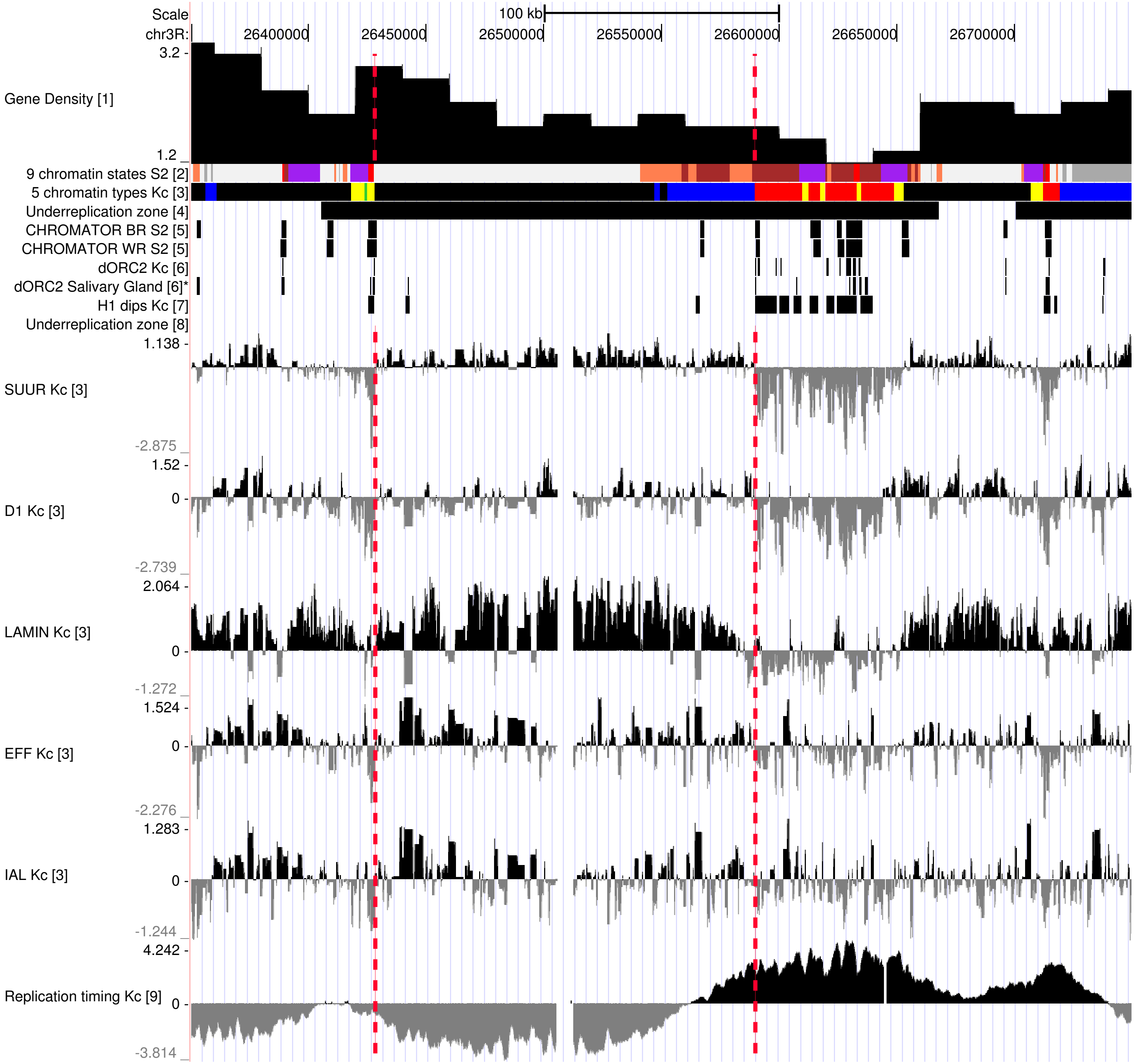

Scale
100 kb
chr3R:
26400000
26450000
26500000
26550000
26600000
26650000
26700000
3.2
1.2
Gene Density [1]
9 chromatin states S2 [2]
5 chromatin types Kc [3]
Underreplication zone [4]
CHROMATOR BR S2 [5]
CHROMATOR WR S2 [5]
dORC2 Kc [6]
dORC2 Salivary Gland [6]*
H1 dips Kc [7]
Underreplication zone [8]
1.138
SUUR Kc [3]
-2.875
1.52
0
D1 Kc [3]
-2.739
2.064
LAMIN Kc [3]
0
-1.272
1.524
0
EFF Kc [3]
-2.276
1.283
IAL Kc [3]
0
-1.244
4.242
Replication timing Kc [9]
0
-3.814

100B1-2

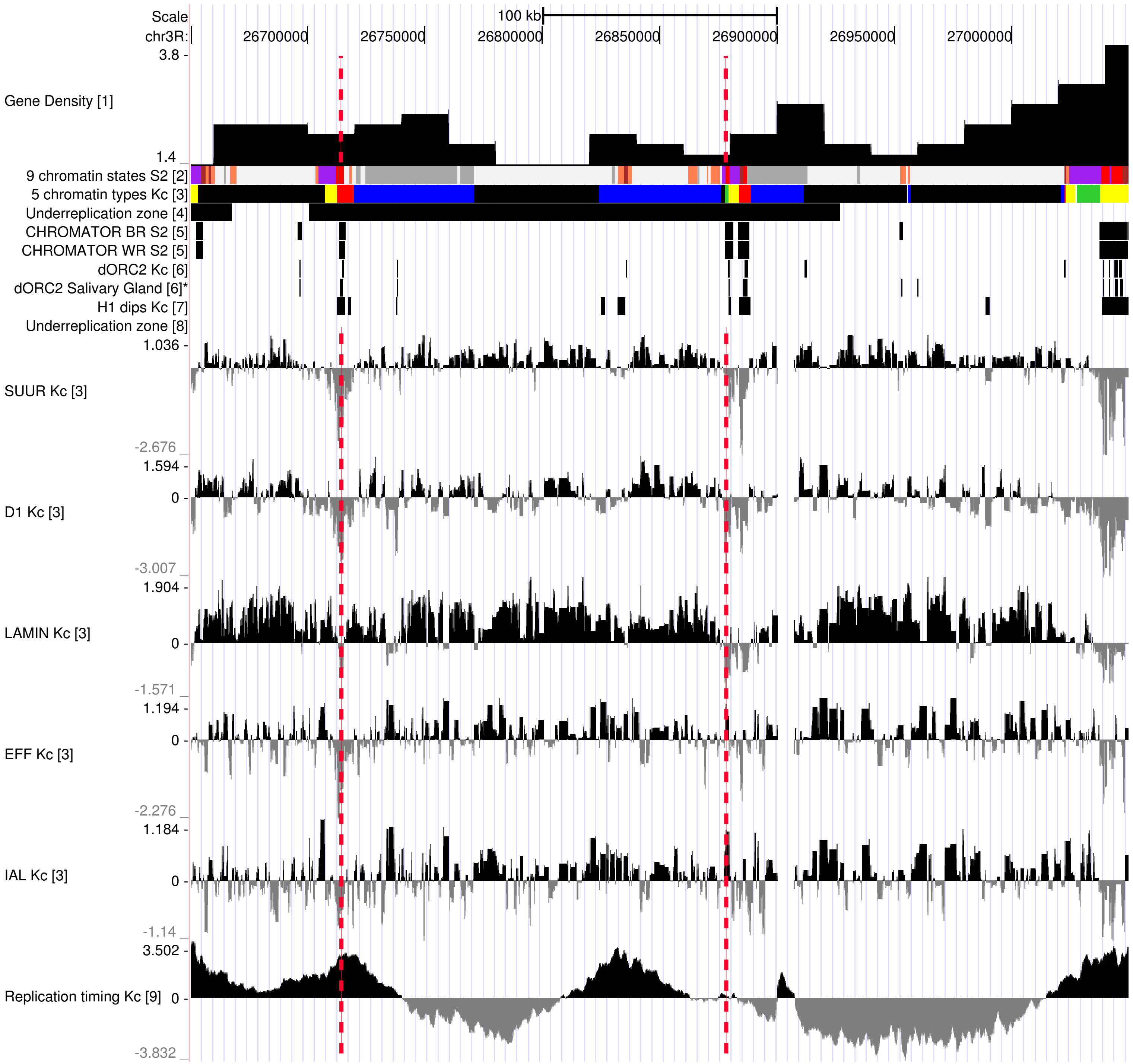

Scale
100 kb
chr3R:
26700000
26750000
26800000
26850000
26900000
26950000
27000000
3.8 -
Gene Density [1]
1.4 _
9 chromatin states S2 [2]
5 chromatin types Kc [3]
Underreplication zone [4]
CHROMATOR BR S2 [5]
CHROMATOR WR S2 [5]
dORC2 Kc [6]
dORC2 Salivary Gland [6]*
H1 dips Kc [7]
Underreplication zone [8]
1.036 -
SUUR Kc [3]
-2.676 _
1.594 -
0 -
D1 Kc [3]
-3.007 _
1.904 -
LAMIN Kc [3]
0 -
-1.571 _
1.194 -
0 -
EFF Kc [3]
-2.276 _
1.184 -
IAL Kc [3]
0 -
-1.14 _
3.502 -
Replication timing Kc [9]
0 -
-3.832 _
